# Elagolix Sodium Salt and Its Synthetic Intermediates: A Spectroscopic, Crystallographic, and Conformational Study

**DOI:** 10.3390/molecules28093861

**Published:** 2023-05-03

**Authors:** Samuele Ciceri, Diego Colombo, Enrico M. A. Fassi, Patrizia Ferraboschi, Giovanni Grazioso, Paride Grisenti, Marco Iannone, Carlo Castellano, Fiorella Meneghetti

**Affiliations:** 1 Department of Pharmaceutical Sciences, University of Milan, Via L. Mangiagalli 25, 20133 Milano, Italy; samuele.ciceri@unimi.it (S.C.); enrico.fassi@unimi.it (E.M.A.F.); giovanni.grazioso@unimi.it (G.G.); 2 Department of Medical Biotechnology and Translational Medicine, University of Milan, Via C. Saldini 50, 20133 Milano, Italy; diego.colombo@unimi.it (D.C.); patrizia.ferraboschi@unimi.it (P.F.); 3 Chemical-Pharmaceutical Consulting and IP Management, Viale G. da Cermenate 58, 20141 Milano, Italy; grisenti.paride60@gmail.com; 4 Tecnomed Foundation, University of Milano-Bicocca, Via Pergolesi 33, 20900 Monza, Italy; marco.iannone@unimib.it; 5 Department of Chemistry, Università degli Studi di Milano, Via Golgi 19, 20133 Milano, Italy; carlo.castellano@unimi.it

**Keywords:** GnRHR antagonist, endometriosis, uterine fibroids, atropisomerism, crystal structure, conformational analysis, NMR spectroscopy

## Abstract

Elagolix sodium salt is the first marketed orally active non-peptide gonadotropin-releasing hormone receptor antagonist (GnRHR-ant) for the management of hormone dependent diseases, such as endometriosis and uterine fibroids. Despite its presence on the market since 2018, a thorough NMR analysis of this drug, together with its synthetic intermediates, is still lacking. Hence, with the aim of filling this literature gap, we here performed a detailed NMR investigation, which allowed the complete assignment of the ^1^H, ^13^C, and ^15^N NMR signals. These data allowed, with the support of the conformational analysis, the determination of the stereochemical profile of the two atropisomers, detectable in solution. Moreover, these latter were also detected by means of cellulose-based chiral HPLC, starting from a sample prepared through an implemented synthetic procedure with respect to the reported ones. Overall, these results contribute to further understanding of the topic of atropisomerism in drug discovery and could be applied in the design of safe and stable analogs, endowed with improved target selectivity.

## 1. Introduction

**Figure 1:**
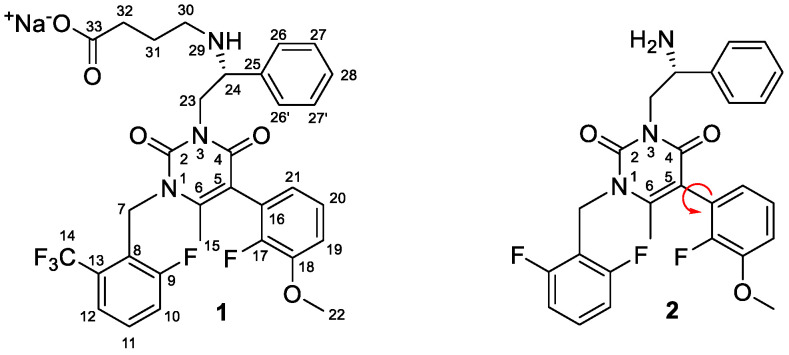
Chemical structures of **1** and its analogue **2**; the atropisomerism in **2** arises from hindrance to rotation about the single bond indicated by the red arrow.

Elagolix sodium salt (CAS number 832720-36-2, **1**, Figure 1) is the first non-peptide orally active gonadotropin-releasing hormone receptor antagonist (GnRHR-ant), approved since 2018 by FDA for the management of moderate-to-severe endometriosis-associated pain [1] and, more recently, against the symptoms related to uterine fibroids [2]. The oral bioavailability of **1** represents a breakthrough with the past, ensuring a rapid, dose-dependent, and reversible suppression of the hypothalamic–pituitary–gonadal axis, not achievable with the peptide analogs marketed before elagolix sodium salt **1** [3,4].

Chemically, compound **1** is an uracil-based derivative substituted at positions 1,3,5, and 6, bearing a stereocenter in (*R*)-configuration. For its close analogue **2**, the literature data [5] reported a decreased rotation of the C-C bond evidenced in red in Figure 1, due to the interaction of the *o*-fluorine of the 5-aryl group with the methyl at the 6-position of the uracil moiety and the electronegative oxygen atom of the carbonyl at the 4-position. Therefore, two atropisomers were detectable under physiological conditions and, with a half-life (t_1/2_) of ~45 min, **2** belongs to the atropisomeric class 2, which includes atropisomers with a rate of interconversion ranging between minutes and a few months, as classified by LaPlante et al. [6]. Atropisomerism arises from the hindered rotation about single bonds; several drugs present this type of isomerism [7,8,9] and can be resolved or not, depending on the extent of the hindered rotation. For example, the atropisomers of the oral uric acid transporter 1 (URAT1) inhibitor lesinurad [10], belonging to class 3, were resolved through a semipreparative enantioselective supercritical fluid chromatography (SFC). On the contrary, the atropoisomers of drugs belonging to class 1, such as sildenafil (Viagra^®^) [11], cannot be resolved. For class 2 [6,12,13], the moderate interconversion rate between atropisomers causes issues in the drug manufacturing and quality control processes, such as a low batch-to-batch reproducibility, and inconsistencies in the safety/efficacy evaluation of the drug. A valuable strategy in avoiding the obtainment of class 2 atropisomers can be the elimination of the source of atropisomerism or the rigidification of the molecular structure. These strategies have been already applied to some elagolix analogues [12,14]. Moreover, the two atropisomers could have different pharmacological behaviors [14]. For **1**, although it shares with **2** the same biaryl system responsible for atropisomerism, data related to atropisomers are absent in the literature. 

The crystal structure of elagolix bound to human GnRHR was recently reported [15], pointing out the shallow non-peptide antagonist binding site and the key regions for ligand recognition: the pyrimidine ring, the benzyl group at position 1 of the uracil moiety, and the phenyl groups at position 5, without mentioning atropisomerism. 

Although the synthesis of **1** was previously described in a 2005 patent [16] and in a 2008 paper [17], the literature requires a comprehensive ^1^H, ^13^C, and ^15^N NMR study of this drug and its synthetic intermediates. Firstly, only the list of ^1^H NMR resonances of **1** and some intermediates was reported [16,17]. Then, in an international patent application [18], the previously non-assigned ^1^H NMR signals of the most advanced intermediate of **1** (the corresponding ethyl ester) were added. In 2018, two international applications, the first regarding novel solid forms of **1** and its ethyl ester [19], and the second concerning a modified synthetic pathway [20], listed the ^1^H NMR data of **1** and its primary intermediates, without their assignments. In 2021, the lists of ^1^H or ^13^C resonances of **1** and some intermediates, without the relative assignments, were published [21,22,23]. More recently [24], the degradation products of **1** were studied; although the ^1^H and ^13^C NMR resonances of **1** and of two oxidation products were completely assigned, the atropisomerism was not mentioned.

Here, in a continuing effort to fully characterize molecules exerting important therapeutic activities [25,26,27], a complete NMR (^1^H, ^13^C, ^15^N) characterization of **1**, and its main synthetic intermediates was carried out, taking in account not only that **1** has a proved pharmacological activity as GNRHR-ant, but also one of its advanced synthetic intermediates [17]. In addition, we obtained single-crystals of the first intermediate endowed with the biaryl system responsible for atropisomerism, suitable for the investigation of its solid-state structure by X-ray diffraction (SC-XRD). Furthermore, a detailed conformational analysis was performed on **1**, to shed some light onto the geometry of low energy conformers. Taken together, these outcomes allowed the determination of the stereochemical profile of the two atropisomers of **1** detectable in solution.

The samples of **1** and its intermediates, required for the analytical characterization, were prepared in order to implement some modifications concerning literature procedures [16,17,28]. 

Overall, the results here obtained could be considered, both from the synthetic and analytical points of view, as a valuable starting point for the design of safe and stable analogs, endowed with improved target selectivity.

## 2. Results and Discussion

### 2.1. Chemistry

The synthesis of **1**, performed following the literature procedure reported in Ref. [17], led to the obtainment of a poor-quality product (86% HPLC purity) and in a low overall yield (8%). These outcomes prompted us to introduce some modifications, in order to improve the quality and yield of **1**, using cheaper and safer reagents. 

**Scheme 1:**
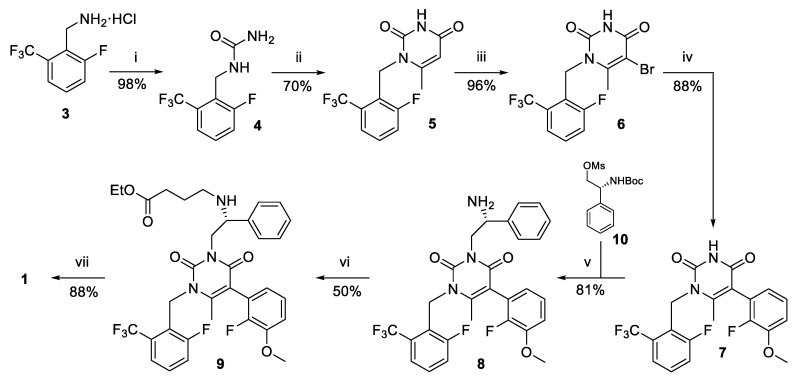
Reagents and conditions: (i) urea, HCl, H_2_O, reflux; (ii) (a) *tert-*butyl acetoacetate, toluene, reflux, Dean-Stark apparatus; (b) p-TsOH**·**H_2_O, reflux; (iii) Br_2_, acetic acid; (iv) 2-fluoro-3-methoxyphenylboronic acid, Pd(OAc)_2_, [(*t*-Bu)_3_PH]BF, KOH, H_2_O, acetone, under argon atmosphere, reflux; (v) (a) **10**, K_2_CO_3_, DMF; (b) MsOH; (vi) (a) ethyl 4-bromobutyrate, DIPEA, DMF, 60 °C; (b) filtration on silica; (vii) (a) NaOH, H_2_O, ethanol; (b) extraction with methyl isobutyl ketone (MIBK); (c) precipitation from heptane.

Starting from the commercially available compound **3** (Scheme 1), the suitable precursor of the uracil moiety **4** was prepared, in 98% yield.

**Scheme 2:**
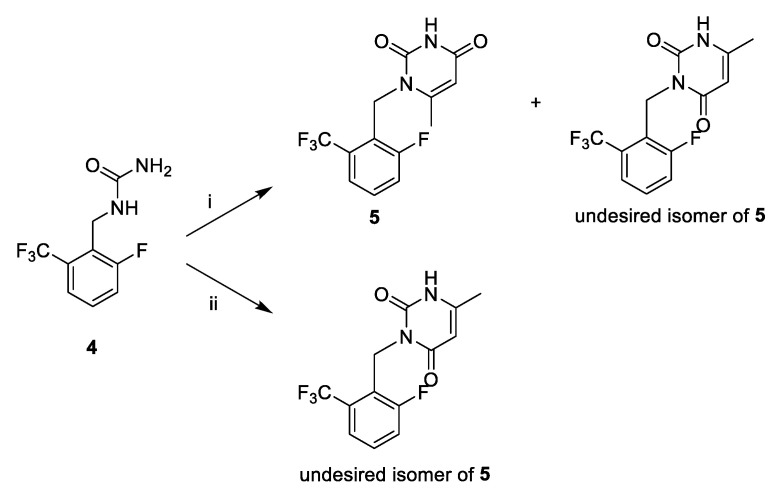
Reaction of intermediate **4** with (i) ethyl aceto-acetate and (ii) diketene-acetone adduct.

To build the 6-methyl uracil ring, instead of hazardous and unstable diketene [16,17], we chose the safer *tert*-butyl acetoacetate, according to a 2009 patent [28], obtaining **5** in 70% yield (Scheme 1). Indeed, unlike *tert*-butyl acetoacetate, diketene readily polymerizes on standing [29], with a high risk of evolution of toxic gases and explosion [30]. Our attempt to use in the synthetic route the cheaper ethyl acetoacetate afforded a mixture of **5** and its isomer (1:0.6 by NMR). In contrast, by using the more stable diketene-acetone adduct, if compared with the diketene, the undesired isomer of **5** was obtained almost quantitatively, contrary to what is reported in Ref. [20]. (Scheme 2, i and ii, respectively). 

**Figure 2:**
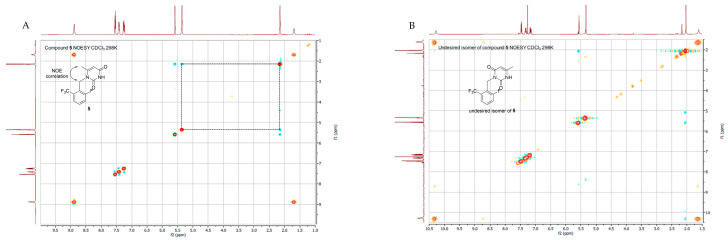
NOESY experiments of compound **5** (**A**) and its isomer (**B**).

The ^1^H two-dimensional nuclear Overhauser effect spectroscopy (NOESY) experiment allowed understanding of the regiochemical outcome of the synthesis of **5,** starting from **4**. The presence of a cross peak between the methyl group at the 6-position of the uracil moiety (2.15 ppm) and the benzyl protons (5.36 ppm) confirmed their proximity (Figure 2A), whereas, for the undesired isomer of **5,** this correlation is not present (Figure 2B). 

**Scheme 3:**
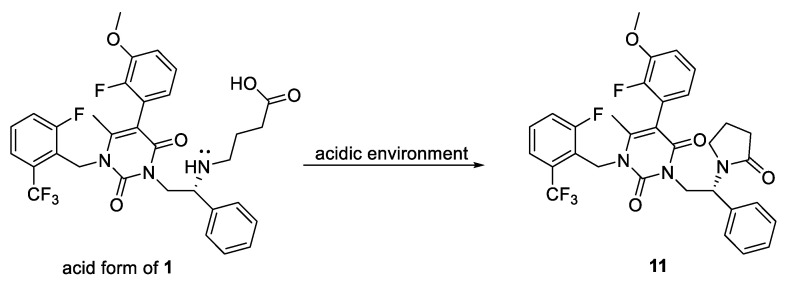
Formation of the main by-product **11**, observed when the literature procedure [11] was applied.

The synthetic steps from **5** to **1** were carried out as described in the 2009 patent [28], but employing intermediate **6** instead of its iodo-analog, to avoid the use of the highly toxic and expensive iodine monochloride. Therefore, we performed a careful optimization of the bromination of **5** to obtain **6**. Indeed, under the experimental conditions reported in the 2008 paper [17] (slow addition of 2.0 eq of bromine to a 1.1 M solution of **5** in acetic acid), we obtained **6** from **5** in a poor yield (43%). On the contrary, the slow addition of a 1.1 M solution of bromine (1.1 eq) to a 0.25 M solution of **5**, both in acetic acid, allowed us to obtain **6** in a higher yield (96%). From intermediate **6**, through a Suzuki cross coupling with 2-fluoro-3-methoxyphenylboronic acid, catalyzed by bis(tri-*tert*-butyl-phosphine)palladium(0), derivative **7** was directly recovered in 88% yield by precipitation from the reaction mixture; pure **8** was obtained in 81% yield through nucleophilic substitution by **7** on (*R*)-2-((*tert*-butoxy-carbonyl)amino)-2-phenylethyl methane-sulfonate **10**, followed by *N*-Boc deprotection with methane-sulfonic acid. Otherwise, reproducing the 2008 synthetic procedure [17], **8** was obtained through a Mitsunobu-like reaction in **6** followed by Suzuki cross coupling catalyzed by tetrakis(triphenylphosphine)palladium(0), but each of these two steps required a careful chromatographic purification to remove the by-products triphenylphosphine oxide, *tert*-butyl hydrazine dicarboxylate, and the palladium catalyst. At the end, the ethyl ester **9**, obtained in 50% yield by N-alkylation of **8** with ethyl 4-bromobutyrate, was hydrolyzed with sodium hydroxide to afford **1**, isolated in 88% yield by extraction with methyl isobutyl ketone, followed by precipitation from n-heptane. The overall yield of this process was 21% and the HPLC purity of **1** was improved to 99.9%. In comparison with the salt formation described in the original synthesis [17], in which the isolated elagolix acid form was converted into **1** employing an ion exchange resin, the direct extraction of the sodium salt from the alkaline hydrolytic reaction mixture [28] was chosen to avoid the formation of the lactam impurity **11** (Scheme 3). We isolated and characterized **11** when we followed the literature procedure [11] (Supplementary Materials, Figures S43 and S44). 

### 2.2. NMR Spectroscopy

A detailed NMR study was carried out on **1** and its precursors **5**–**9** (Scheme 1). ^1^H NMR spectra of **1** were recorded in deuterated methanol (CD_3_OD), for a better comparison with the signals already reported in the literature [17], while the spectra of intermediates **5** and **7**–**9** were recorded in deuterated chloroform (CDCl_3_) at 298 K. A mixture of CDCl_3_/CD_3_OD 9:1 was used for **6**, due to its poor solubility in CDCl_3_. Table 1, Table 2 and Table 3 summarizes the unambiguous assignments of all ^1^H, ^13^C and ^15^N signals, established by combining the information gathered from 1D NMR spectra and 2D homo-correlated (COSY and NOESY) and hetero-correlated (^1^H–^13^C HSQC, ^1^H–^13^C HMBC, and ^1^H-^15^N HMBC) NMR spectra. Atoms were numbered as reported in Figure 1. Most of the proton assignments were accomplished using general knowledge of chemical shift dispersion, with the aid of the proton–proton coupling pattern (^1^H NMR spectra), gs-COSY, and NOESY experiments. In ambiguous cases, gs-HSQC and gs-HMBC spectra were used as a definitive and unequivocal tool to make specific assignments, especially for quaternary carbons. For the attribution of ^13^C NMR resonances, in some cases, the analysis of the *^n^J*_13C-19F_ [31] turned out to be very useful, although this coupling increased the complexity of the spectrum.

**Figure 3:**
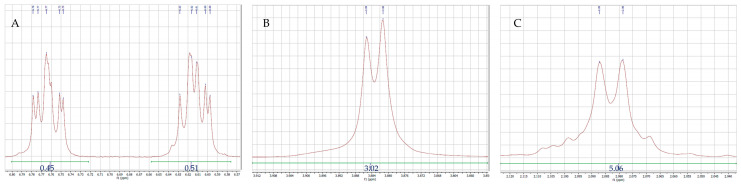
(**A**) H-21, (**B**) H-22, and (**C**) H-15 signals of **1**. The integration values are reported in blue. The H-15 signal is overlapped with H-32 signals.

In particular, for compounds **5**–**7**, the resonances of all protons were assigned using general knowledge of chemical shift dispersion, with the aid of the proton–proton coupling pattern and the COSY experiment for the aromatic protons. In the case of H-10, an additional coupling constant is recognizable (*J*_1H-19F_ = 12.3 Hz) due to the presence of fluorine at the 9-position. The correct structural features of **5** were verified by a NOESY experiment. Indeed, as shown in Scheme 2 two different isomers can be obtained under these reaction conditions. The presence of a cross peak between the three H-15 (2.15 ppm) and the two H-7 (5.36 ppm) in the NOESY spectrum of **5** (Figure 2A), absent in the spectrum of its isomer (Figure 2B), confirmed its correct chemical structure. Starting from compound **8**, obtained from **7** by introduction of the (*R*)-phenylamino ethyl moiety at the 3-position of the uracil core, for all the following intermediates, two distinct resonances for the H-21 proton (6.85 and 6.78 ppm, 6.83 and 6.76 ppm, and 6.76 and 6.61 ppm for **8**, **9**, and **1**, respectively), in a ratio 1:1.07, 1:1.04, and 1:1.13 (Figure 3A), were observed. The same phenomenon was detected, though less conspicuous (distance between the two signals ≤ 0.01 ppm), for H-15 and H-22 signals of **9** and **1** (Figure 3B,C). The presence of a (*R*)-stereocenter in compounds **8**, **9**, and **1**, together with the atropisomerism, explained the two signals observed in their spectra [5] (Figure 3). Furthermore, the ratio of the two signal sets, being different from 1.00, indicated that one of the two atropisomers was favored.

The absence of any mention of atropisomerism in Ref. [24] could be explained by the choice of DMSO-d_6_ as NMR solvent. Indeed, the ^1^H-NMR spectrum registered in this solvent is less indicative of highlighting the simultaneous existence of two diastereoisomers of **1**, due to the presence of both the (*R*)-stereocenter and atropisomerism. From the comparison between the ^1^H NMR spectrum registered in CD_3_OD and that in DMSO-d_6_, it is evident that the most significant signal shown in the CD_3_OD spectrum (H-15 protons) is only partially resolved in DMSO-d6 (see Table 3 of reference [24] and the enclosed spectra in the Supplementary Materials Figure S41 of the present article). As a consequence, the signals due to the H-21, well resolved in both spectra, were not correctly assigned and integrated [24]. 

NOESY experiments performed on **1** dissolved in CD_3_OD revealed the proximity, within 5.0 Å, of H-15 (2.085 and 2.076 ppm) to H-21 (6.76 and 6.61 ppm) and H-7 (5.43 ppm), of H-19 (7.10 ppm) to H-22 (3.883 and 3.880 ppm), and of H-30 (2.40 ppm) to H-23 (4.26–4.07 ppm), H-24 (4.11 ppm), and H-26/H-26’ (7.29 ppm), in both atropisomers (Supplementary Materials Figure S40). 

The assignments of carbon atoms of CH, CH_2_ and CH_3_ groups were confirmed by the gs-HSQC experiment. The quaternary carbon atoms were unambiguously assigned using the information obtained from the ^1^H–^13^C gs-HMBC experiment (the observed couplings for **1** are listed in Table 4) and from ^n^*J*_13C-19F_ [31]. Due to atropisomerism, the ^13^C NMR spectra of compounds **8**, **9**, and **1** showed, for some carbon atoms, two singlets very close to each other (Table 2). In this case also, the ^13^C signals indicated a relative ratio between atropisomers other than 1.00.

^1^H-^15^N HMBC experiments were performed to further support the proposed structures of the studied compounds. Resonances of N-1, and N-3 were assigned for all compounds, and also of N-29 for **8**, **9** and **1**. The value of the chemical shift of N-1 remained almost unchanged from intermediate **5** to **1**. On the contrary, the value of the chemical shift of N-3 for **5**, **6**, and **7** changed from 154.3, 153.4, and 151.9 ppm to 160.1, 161.3, and for **8**, **9**, and **1** to 160.5 ppm as a result of the N-alkylation of this nitrogen [32]. The same behavior was observed for N-29, whose chemical shift value changed from 31.2 ppm for **8** (primary amine) to 43.6 and 46.3 for **9** and **1** (secondary amine), respectively. For all compounds, the ^1^H–^15^N gs-HMBC spectra showed cross peaks of H-15 and H-7 with N-1, while, in the case of N-3 and N-29 of **8**, **9**, and **1**, the cross peaks H-24/N-3, H-23/N-29, H-24/N-29 and H-31/N-29 were observed.

### 2.3. HPLC Analyses

**Figure 4:**
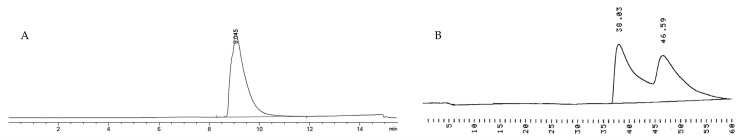
HPLC chromatogram of **1** performed on (**A**) RP-C18 and (**B**) chiral stationary phase.

The chromatogram of the HPLC analysis performed on **1** using a non-chiral reverse stationary phase (RP-C18) showed a broad peak, indicating the presence of two very similar but not distinguishable molecules (Figure 4A, see Section 3.2.8 and Section 3.4 for HPLC conditions). On the contrary, the HPLC analysis on a chiral stationary phase of cellulose tris(3,5-dimethylphenylcarbamate) (Figure 4B, see Section 3.2.8 and Section 3.4 for HPLC conditions) clearly showed the presence of two distinct peaks, corresponding to the two atropisomers (diastereoisomers) of **1**.

**Figure 5:**
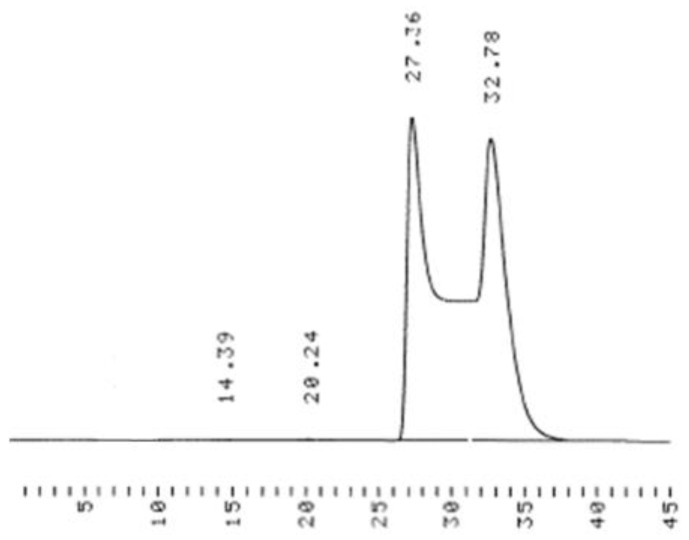
Chiral HPLC chromatogram of **7**.

Since **7** is the first intermediate carrying the biaryl system involved in the atropisomerism, we decided to analyse **7** by chiral HPLC (see section Section 3.2.4 and Section 3.4 for HPLC conditions). As shown in Figure 5, two peaks are detectable, confirming the existence of two atropisomers (enantiomers) in this compound also.

As reported in some literature examples of gas chromatographic (GC) and HPLC analysis of stereo-labile chiral compounds [33,34,35,36], the observed chromatographic profile, with a plateau between the two chromatographic peaks that does not reach the baseline, indicates the existence of a dynamic interconversion of the two atropisomers within the time scale of the analysis, the so called “on-column interconversion”.

### 2.4. Structure Description

For **7**, the first synthetic intermediate bearing the biaryl system contributes to atropisomerism, so we also decided to analyze its solid-state structure through SC-XRD. Single crystals were obtained by the slow evaporation of a MeOH solution after 1 week. Crystallographic data and refinement details are given in Section 3. 

**Figure 6:**
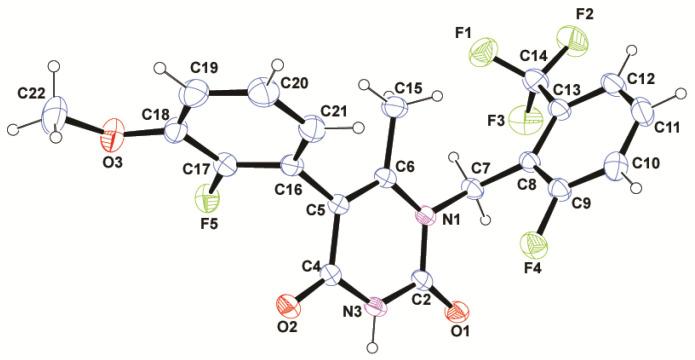
ORTEP [37] diagram of **7** with an arbitrary atom-numbering scheme. Thermal ellipsoids were drawn at the 40% probability level.

Compound **7** crystallized in the orthorhombic achiral space group P bca; its structure is shown in Figure 6 as an ORTEP diagram [37], indicating the arbitrary atom-numbering scheme used in the following discussion.

The overall molecular structure of the racemate was characterized by a dihydro-pyrimidine nucleus bound to a 5-(2-fluoro-3-methoxyphenyl moiety and a (trifluoromethyl)benzyl group. The angles between the best mean plane calculated for the heterocyclic ring and these two aromatic portions were 87.4(1)° and 67.2(1)°, respectively. The dihydro-pyrimidine was nearly planar, with a maximum deviation of 0.021(1) Å. Moreover, the conformation of the molecule was also characterized by the dihedral angles C2-N1-C7-C8 of −105.3(1)°, N1-C7-C8-C13 of −144.7(3)° and C4-C5-C16-C21 of 110.4(3). 

**Figure 7:**
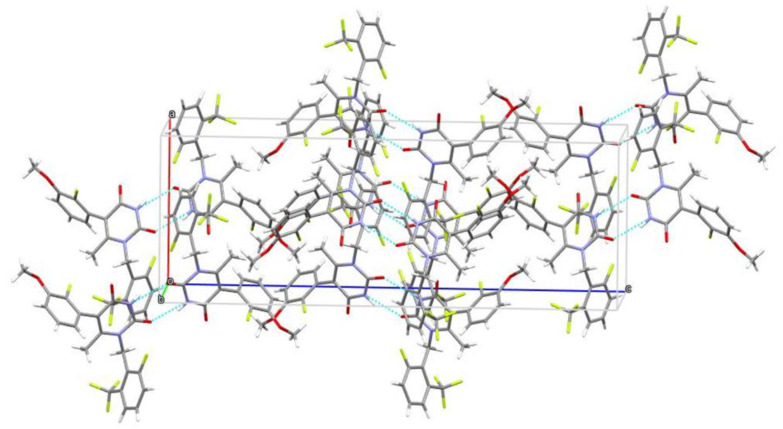
Graphical representation of the crystal packing along a axis, showing the H-bonds as dotted blue lines.

The crystal packing, shown in Figure 7, was consolidated by strong dimeric H-bonds involving N3-H∙∙∙O1^I^ (^I^ at 1 − x, −y, −z); the donor-acceptor (D∙∙∙A) distance is 2.87(1) Å, the hydrogen and the acceptor (D-H∙∙∙A) are at 2.063(2) Å, and the angle is 155.2(5)°. These H-bonds give rise to the formation of molecular chains along the a axis. Parallel π-π stacking interactions between (trifluoromethyl)benzyl moieties were present and further contributed to the crystal packing: the distance between the centroids was 3.20(6) Å, while the angle between the centroid-centroid vector and the plane normal was 3.62(4)°. Loose CH∙∙∙O and CH∙∙∙F contacts contributed to the stabilization of the crystal structure; these non-traditional H-bonds were established between C12-H∙∙∙O2^II^, D∙∙∙A = 3.26(8) Å, D-H∙∙∙A = 2.63(1) Å, angle = 124.3(9)° (^II^ at 1 + x, y, z), C11-H∙∙∙F4^III^, D∙∙∙A = 3.42(8) Å, D-H∙∙∙A = 2.54(1) Å, angle = 163.5(9)° (^III^ at ½ + x, ½ − y, z) and C19-H∙∙∙F5^IV^, D∙∙∙A = 3.37(8) Å, D-H∙∙∙A = 2.48(1) Å, angle = 160.3(9)° (^IV^ at x, ½ + y, ½ − z). The Van der Waals interactions between the aromatic rings of adjacent molecules favored a two-dimensional-sheet structure, in which the molecules in the two layers are inclined as shown in Figure 7.

It can be hypothesized that the crystallization conditions (methanol + 5% water) facilitated the solubilization of the compound and promoted the formation of a dense network of interactions, which ultimately allowed the orderly organization of the molecules to form a crystal.

### 2.5. Conformational Analysis

**Figure 8:**
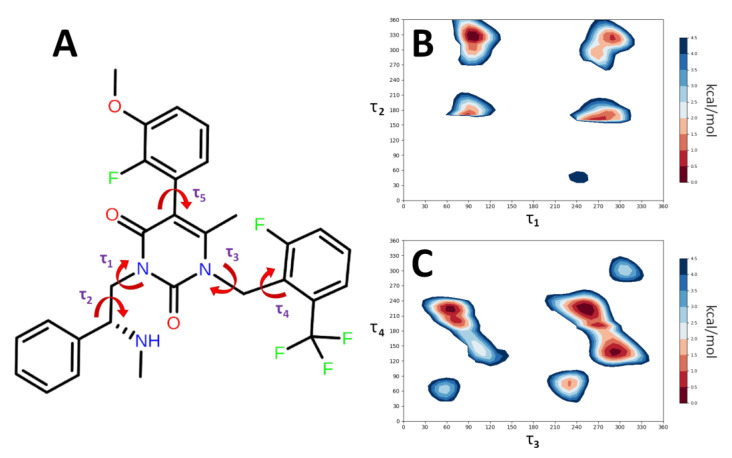
(**A**). Simplified structure of **1** used for conformational analysis studies. The yellow arrows highlight the dihedral angles investigated by means of the conformational analysis. (**B**,**C**) Scan plots of the τ_1_–τ_4_ torsional angles.

With the aim of rationalizing the obtained results, the conformational behavior of **1** was investigated by means of computational tools. The structure of **1** was simplified, as reported in Figure 8A, shortening the lateral chain carrying the carboxylate moiety, to reduce the number of dihedral angles to be scanned. Then, the conformational analysis was performed through a mixed molecular mechanics-DFT/B3LYP-D3/6-31G(**) level approach in the gas phase, and later in water and methanol as solvents (see Section 3 for details). 

The four torsional angles of the simplified structure of **1** were initially scanned by increments of 10 degrees using the MacroModel tool of Maestro (release 2021-2, Schrödinger, LLC, New York, NY, USA), applying the OPLS4 force field. For simplicity, these calculations were conducted in two independent steps to identify the combination of torsion angles leading to low energy conformers. Firstly, τ_1_ and τ_2_ were combined and scanned (Figure 8B), and successively τ_3_ and τ_4_ were considered (Figure 8C). The torsion angles/energy 3D plots shown in Figure 8B,C displayed the low energy conformers’ combinations. Of note, only four τ_1_/τ_2_ combinations were energetically favored, while the τ_3_/τ_4_ combinations led to six different low energy conformers. Then, the τ_1_–τ_4_ torsion angles’ values leading to the lowest energy conformers were systematically combined, obtaining a library of 24 different conformers, which were successively optimized in the gas phase (Table 5). At a later stage, the energy of each optimized structure was recalculated in water (as single point calculation), and finally in methanol, the solvent employed in the NMR experiments (see Supplementary Materials, Table S1). These calculations were performed applying the DFT/B3LYP-D3/6-31G(**) level of theory [38,39], using the Jaguar module of Maestro (release 2021-2, Schrödinger, LLC, New York, NY, USA). Notably, some of them converged into the same energy minima conformer; in particular, **1N** converged to **1T**, while **1P** converged to **1R** (Table 5). 

Finally, the τ_5_ dihedral angle, the one capable of generating the atropisomerism phenomena, was scanned in the lowest energy conformer of the simplified structure of **1** (Figure 8A, conformer **1M**, Table 6). 

**Figure 9:**
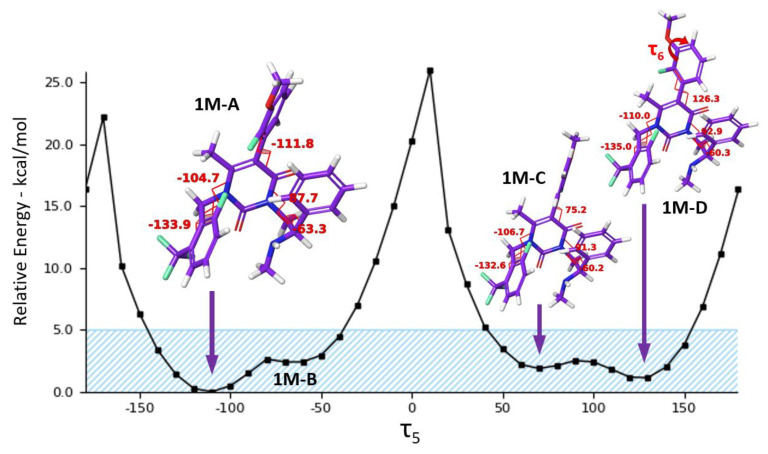
Coordinate scan plot related to the τ_5_ torsional angle variation and low energy conformers, resulting from the data reported in Table 7.

These calculations were performed scanning the τ_5_ torsion angle, by an increment of 10° (from −180° to +180°). We contextually optimized the conformers at DFT/B3LYP-D3/6-31G(**) level of theory, using the CPCM water solvent model (see Section 3 for details). The achieved results (Figure 9, top panel) suggested the presence of four different energy minima conformers. Their geometries were geometrically optimized at the same level of theory, in order to improve the accuracy of these results. The outcomes showed that **1M-B** converged into the minimum energy **1M-A**, while **1M-C** and **1M-D** represented another two relative minimum energy conformers (Table 7). Single point calculations using the CPCM methanol solvent model (which is the solvent employed in the NMR experiments), displayed results similar to those observed using the CPCM water solvent model.

To conclude, **1M-A**, the atropisomer a*R*, was the most abundant (83.6%) at room temperature, while **1M-C** and **1M-D,** corresponding to the opposite atropisomer a*S*, respectively, composed the remaining 16.4%, in both solvent models (water and methanol). Since one atropisomer is more abundant than the other in methanol solution, and this finding was in agreement with the NMR data (see Section 2.2.), the diagnostic H-21 (6.61 ppm), H-22 (3.880 ppm), and H-15 (2.076 ppm) signals can be assigned to the most abundant atropisomer a*R*, while the H-21 (6.76 ppm), H-22 (3.883 ppm), and H-15 (2.085 ppm) signals to the least abundant atropisomer a*S*.

Additionally, since the methoxy group is one of the key elements of the uracil pharmacophore as GnRHR-ant [3,40], its position was investigated in the lowest energy conformers of **1M-A** and **1M-D**. In particular, the dihedral angle highlighted by the red arrow in **1M-D** (Figure 9) was rotated, forming the two conformers **I** and **II**. Here, the H-22 atoms were oriented in the direction of C15 (t_6_ = −66°, distance C_H22_-H19 = 3.6 Å) in **I**, and in opposite direction (t_6_ = +66°, distance C_H22_-H19= 3.6 Å) in **II**. The structures of the latter were different from those of the energy minima **1M-A** and **1M-D**, characterized by t_6_ of 0° and having the C_H22_-H19 distance of 2.56 Å. Energy optimization calculations at DFT/B3LYP-D3/6-31G(**) level of theory [38,39], in the gas phase and in CPCM methanol solvent model [41], suggested that the conformers with the lowest distance between C_H22_ and H19 (t_6_ = 0°) were the preferred ones when the CPCM methanol solvent model was used [41]. This outcome agreed with the presence of a cross peak between the H-19 (7.10 ppm) and H-22 (3.883 and 3.880 ppm) protons of the two atropisomers in the NOESY spectrum of **1** (Supplementary Materials Figure S40) and with the conformation of the solid-state structure determined for intermediate **7**. Conversely, in the gas phase, the population of **1M-D** conformers with distance C_H22_-H19 = 3.6 Å was at 69.5%, while that for the same conformers of **1M-A** was 55.5% (Table 7). 

## 3. Materials and Methods

### 3.1. General

All reagents and solvents were purchased from Sigma-Aldrich (Merck Life Science S.r.L., Milano, Italy). TLC analyses were performed on silica gel 60 F254 plates, precoated with a fluorescent indicator (Merck Life Science S.r.L., Milano, Italy); spots were detected by UV lamp 254 nm, or by a 0.3% w/v ninhydrin solution in n-butanol/acetic acid (100:3) and heating at 110 °C.

Optical rotation values were registered on an Anton Paar instrument (Mod MCP 100; Anton Paar Strasse 10, 8054 Graz, Austria) at 589 nm and 25 °C. 

### 3.2. Synthesis of 1 from 3

#### 3.2.1. 1-(2-fluoro-6-(trifluoromethyl)benzyl) urea (4)

Compound **4** was synthesized, starting from 2-fluoro-6-(trifluoromethyl)benzylamine, as reported in Ref. [17] in 98% yield. 

R_f_ 0.60 (CH_2_Cl_2/_MeOH 9:1)

^1^H NMR (500 MHz, DMSO) δ 7.61-7.56 (m, 3H), 6.15 (t, *J* = 5.3 Hz, 1H), 5.47 (s, 2H), 4.36 (d, *J* = 5.0 Hz, 2H). 

The other physico-chemical properties were in agreement with the reported ones [17,42].

#### 3.2.2. 1-(2-fluoro-6-(trifluoromethyl)benzyl)-6-methylpyrimidine-2,4(1H,3H)-dione (5)

Compound **5** was synthesized, starting from **4**, as in Ref. [28], in 70% yield. The physico-chemical properties were in agreement with those reported [17,28].

R_f_ 0.27 (hexane/ethyl acetate 1:1) 

#### 3.2.3. 5-bromo-1-(2-fluoro-6-(trifluoromethyl)benzyl)-6-methylpyrimidine-2,4(1H,3H)-dione (6)

A solution of bromine (2 mL; 38.5 mmol) in glacial acetic acid (36 mL) was slowly added (in about 20 min) to a solution of **5** (10.58 g; 35 mmol) in glacial acetic acid (140 mL) under vigorous stirring. The reaction progress was monitored by TLC analysis (hexane/ethyl acetate 7:3) until starting material disappearance (2h). The excess of bromine was removed bubbling nitrogen through the reaction mixture until almost complete disappearance of its colour. The reaction mixture was concentrated at reduced pressure (60 °C, 30 mmHg), affording a yellow residue which was suspended in *tert*-butyl methyl ether (25 mL). The suspension was stirred at room temperature for 2 h, filtered by suction and washed with *tert*-butyl methyl ether (5 mL). The solid was dried (60 °C, 2 mmHg, 8 h) affording **6** (12.86 g, 33.7 mmol; 96%). The physico-chemical properties were in agreement with those reported [17].

R_f_ 0.69 (hexane/ethyl acetate 1:1)

#### 3.2.4. 5-(2-fluoro-3-methoxyphenyl)-1-(2-fluoro-6-(trifluoromethyl)benzyl)-6-methylpyrimidine-2,4(1H,3H)-dione (7)

An aqueous solution (10 mL) of KOH (4.64 g; 82.70 mmol) was added to a mixture of **6** (7.7 g; 20.20 mmol) and 2-fluoro-3-methoxy-phenyl boronic acid (4.12 g; 24.24 mmol) in acetone (30 mL) and water (20 mL). The obtained solution was degassed (bubbling argon for about 30 min) and tri-*tert*-butyl-phosphonium tetrafluoroborate (62 mg; 0.21 mmol) was added. After 20 min at 45 °C, Pd(OAc)_2_ (23 mg; 0.10 mmol) was added. The reaction mixture was refluxed under argon atmosphere and stirred while the reaction progress was monitored by TLC analysis (hexane/ethyl acetate 1:1) until disappearance of the starting material (24 h). The reaction mixture was cooled to 55 °C and glacial acetic acid (3.5 mL) was added and further stirred for 30 min. A white precipitate formed. The reaction mixture was cooled to room temperature and stirred at this temperature for 2 h. The white precipitate was filtered by suction and washed with water (15 mL) and then with methanol (30 mL). The solid was dried (60 °C; 2 mmHg; 8 h) affording 7.38 g (86% yield). The physico-chemical properties were in agreement with those reported [28,43].

R_f_ 0.36 (hexane/ethyl acetate 1:1)

RP-HPLC analysis (elution in gradient mode: Mobile phase: A = water with 0.05% trifluoroacetic acid; B = acetonitrile with 0.05% trifluoroacetic acid. Gradient: 95%A/5%B to 5%A/95%B over 50 min, then 5%A/95%B to 1%A/99%B over 0.1 min, then hold 1%A/99% for 0.8 min and back to 95%A/5% over 0.2 min, hold such gradient for 4 min. Flow rate 2.0 mL/min): t_R_ = 25.1 min.

HPLC on chiral stationary phase (elution in isocratic mode: hexane/IPA 1:1. Flow rate 0.5 mL/min): t_R_ (**I** atropisomer) = 27.4 min, t_R_ (**II** atropisomer) = 32.8 min.

#### 3.2.5. (R)-2-[(*tert*-butoxy-carbonyl)amino]-2-phenylethyl methane-sulfonate (10)

Compound **10** was synthesized, starting from (R)-phenyl-glycinol, as in Ref. [44], in 73% yield. The physico-chemical properties were in agreement with those reported [44].

R_f_ 0.80 (CH_2_Cl_2_/MeOH 95:5)

#### 3.2.6. (R)-3-(amino(phenyl)methyl)-5-(2-fluoro-3-methoxyphenyl)-1-(2-fluoro-6-(trifluoromethyl)benzyl)-6-methylpyrimidine 2,4(1H,3H)-dione (8)

Compound **8** was synthesized, starting from compounds **7** and **10**, as in Ref. [28], in 80% yield. The physico-chemical properties were in agreement with those reported [28,45].

[α]_D_^25^ + 15.1 (c 1, CHCl_3_).

R_f_ 0.17 (hexane/ethyl acetate/ Et_3_N 1:1:0.1)

#### 3.2.7. Ethyl (R)-4-(((5-(2-fluoro-3-methoxyphenyl)-3-(2-fluoro-6-(trifluoromethyl)benzyl)-4-methyl-2,6-dioxo-3,6-dihydropyrimidin-1(2H)-yl)(phenyl)methyl)amino)butanoate (9)

Compound **9** was synthesized, starting from **8**, as in Ref. [28], in 50% yield. The physico-chemical properties were in agreement with those reported [28,45].

[α]_D_^25^ − 5.7 (c 1, CHCl_3_).

R_f_ 0.44 (hexane/ethyl acetate/ Et_3_N 1:1:0.1)

#### 3.2.8. Elagolix Sodium Salt (1)

Compound **1** was synthesized, starting from **9**, as reported in Ref. [28] in 88% yield. 

R_f_ 0.74 (CHCl_3_/MeOH/H_2_O 12.5:4:0.5)

[α]_D_^25^ + 12.2 (c 1, MeOH).

RP-HPLC analysis (elution in gradient mode in agreement with Ref. [28]): t_R_ = 9.0 min.

HPLC on chiral stationary phase (elution in isocratic mode: hexane/IPA 6:4 + 0.1% TFA. Flow rate 0.3 mL/min): t_R_ (**I** atropisomer) = 38.0 min, t_R_ (**II** atropisomer) = 46.6 min.

The other physico-chemical properties were in agreement with those reported [17,28].

### 3.3. NMR Spectroscopy

NMR spectra were recorded on a Bruker AVANCE 500 spectrometer (Bruker, Billerica, MA, USA) equipped with a 5 mm broadband inverse (BBI) detection probe with field z-gradient operating at 500.13, 125.76, and 50.69 MHz for ^1^H, ^13^C, and ^15^N, respectively. The spectra were recorded at 298 K for **5**, and **7**–**9** in chloroform-d (CDCl_3_, isotopic enrichment 99.9 atom % D) and, in the case of **1** and **6**, in methanol-d_4_ (CD_3_OD, isotopic enrichment 99.9 atom % D) and chloroform-d/ methanol-d_4_ 9:1 mixture, respectively. Chemical shifts (d) of the ^1^H NMR and ^13^C NMR spectra are reported in ppm using the signal for residual solvent proton resonance as the internal standard (^1^H NMR: CDCl_3_ 7.26, CD_3_OD 3.31 ppm; ^13^C NMR: CDCl_3_ 77.0 (central line), CD_3_OD 49.00 (central line) ppm). For ^15^N nuclei, in the gs-^1^H-^15^N HMBC experiment, nitromethane was used as the external reference (^15^N at 381.7 ppm [32]) whereas, in the gs-^1^H-^15^N HSQC experiment, ammonia (^15^N at 0.0 ppm [32]) was used. The pulse widths were 8.00 μs (90°) for ^1^H, 13.00 μs (90°) for ^13^C, and 27.50 μs (90°) for ^15^N. Data were collected and processed by XWIN-NMR software (version 3.5, Bruker, Billerica, MA, USA) running on a PC with Microsoft Windows 7. The samples (20 mg) were dissolved in the appropriate solvent (0.75 mL) in a 5 mm NMR tube. The acquisition parameters for 1D were as follows: ^1^H spectral width of 5000 Hz and 32 K data points providing a digital resolution of ca. 0.153 Hz per point, relaxation delay 20 s, “zg” pulse sequence of the Bruker library was used; ^13^C spectral width of 29,499 Hz, and 32 K data points providing a digital resolution of ca. 0.900 Hz per point, relaxation delay 2 s, “zgpg” pulse sequence of the Bruker library was used. The experimental error in the measured ^1^H-^1^H coupling constants was ±0.5 Hz. The splitting pattern abbreviations were as follows: s, singlet; d, doublet; t, triplet; q, quartet; m, multiplet; and br, broad signal. Except for NOESY, standard Bruker microprograms using gradient selection (gs) were applied for two-dimensional experiments. Gs-COSY-45 (“cosygpqf” pulse sequence of the Bruker’s library) and phase-sensitive NOESY (“noesyph” pulse sequence of the Bruker’s library) experiments were acquired with 512 t_1_ increments, 2048 t_2_ points, and a spectral width of 10.0 ppm. The NOESY experiments were performed on samples degassed under a flush of argon in a screwcap sample tube. There were no significant differences in the results obtained at different mixing times (0.5–1.5 s). The acquisition data for gs-HSQC (“hsqcetgp” pulse sequence of the Bruker’s library) and gs-HMBC (“hmbcgplpndqf” pulse sequence of the Bruker’s library) experiments were acquired with 512 t_1_ increments, 2048 t_2_ points, and a spectral width of 10.0 ppm for ^1^H and 240 ppm for ^13^C. Delay values were optimized to 140 Hz for ^1^J_13C,1H_ and 8.0 Hz for ^n^J_13C,1H._

The gs-^1^H-^15^N HMBC (“hmbcgpndqf” pulse sequence of the Bruker’s library) and HSQC (“hsqcetgpsi” pulse sequence of the Bruker’s library) experiments, were performed with 256 t_1_ increments, 1024 t_2_ points, and a spectral width of 10.0 ppm for ^1^H and 600 ppm for ^15^N setting an acquisition time of 0.5 s, a relaxation delay of 2 s, a ^1^J_15N,1H_ value of 90.0 Hz, and a ^n^J_15N,1H_ value of 4.5 Hz. This last parameter was set after several attempts between 1 and 11 Hz. The total experimental time for ^1^H-^15^N gs-HMBC analyses was about 12 h.

The NMR spectra are available in the Supplementary Materials section.

### 3.4. HPLC Analyses

The employed HPLC units were:

− Chiral HPLC analysis: a Merck-Hitachi (Hitachi Ltd., Tokyo, Japan), equipped with a UV detector model L-4250, pump system model L-6200 and a chromato-integrator model D-2500. The column employed in the analyses was a Phenomenex Lux-Cellulose 1 (Phenomenex, Torrance, CA, USA). The dimension of the column is 250 mm × 4.6 mm, 3 µm. The elution was in isocratic mode with the indicated eluant and flow. All the samples were measured at λ = 254 nm and 25 °C.
− RP-HPLC analysis: Agilent 1100 system (Agilent Technologies, Waldbronn, Germany) equipped with a Zorbax SB-C18 column (150 mm × 3.0 mm, 3.5 µm) for **1** and with a Supelco Discovery C18 (250 mm × 4.6 mm, 5.0 µm) for **7**.

All the samples were measured at λ = 254 nm and 25 °C.

### 3.5. X-ray Analysis

X-Ray analyses were performed on a Bruker SMART APEX II CCD Single Crystal X-ray Diffractometer (Bruker, Karlsruhe, Germany), equipped with graphite-monochromated Mo-Kα radiation (λ = 0.71073 Å) at 298(2) K. 

X-ray data were acquired in the θ range 2–20° recording four sets of 360 bidimensional CCD frames with the following operative conditions: omega rotation axis, scan width 0.5°, acquisition time 50 s, sample-to-detector distance 50 mm, phi angle fixed at four different values (0°, 90°, 180° and 270°) for the four different sets. Omega-rotation frames were processed using the SAINT software [46] for data reduction (intensity integration, background, Lorentz, and polarization corrections) and for the determination of accurate unit-cell dimensions. Absorption effects were empirically evaluated by the SADABS software [47], applying an absorption correction to the data.

The crystal structure was solved by direct methods and refined on F^2^ by full-matrix least-squares using Bruker’s SHELXL-2018/1 [48]. 

Details are summarized in Table 8. Crystallographic data were deposited to the Cambridge Crystallographic Data Center under accession number CCDC 2248213. The refined structure was inspected using ORTEP-3 (v. 2020.1) [37] and analyzed by Mercury 4.0 (v. 2021.3.0) [49] and PARST [50], within the WinGX suite (v. 2021.3) [50]. Graphical representations were rendered with Mercury.

### 3.6. Conformational Analysis

These studies were performed using the tools available in Maestro (release 2021-2, Schrödinger, LLC, New York, NY, USA). The simplified structure of **1** (Figure 8A) was created by means of the “Build” tool; the potential energy of the conformers, resulting from the different combination of the *τ*_1_–*τ*_4_ values (as depicted in Figure 8A), was calculated with the “Coordinate scan” tool of Macromodel, using the OPLS4 force field and choosing the proper couple of torsional angles to be scanned in the calculations. The plots reported in Figure 8B,C were obtained by the “plot coordinate scan” tool of Macromodel, focusing the displayed energy values from 0 to 5 kcal/mol. Then, all the energy minima identified by Macromodel, or manually constructed rotating the dihedral angles indicated throughout the Results section, were optimized by using Jaguar, at the DFT/B3LYP-D3/6-31G(**) level of theory [38,39]. 

## 4. Conclusions

In this paper we described an efficient synthesis of elagolix sodium salt (**1**), bearing axial chirality, which was thoroughly explored through spectroscopic, analytical and theoretical techniques. The obtained results offered novel clues to recognize and characterize its atropisomers. To optimize the original synthetic route, we applied a new strategy capable of improving the reaction yields and the purity of the intermediates and of the final product **1**, using less hazardous reagents. The optimized synthetic pathway developed for the obtainment of elagolix sodium salt could be also followed in the next future for the synthesis of the selected analogues. We planned to develop a protocol that could be applied for the high-yield synthesis of new non-peptide congeners of **1**, and to fill a literature gap concerning the NMR data of **1** and its synthetic intermediates, which are essential for comprehensively studying the pharmacological activity and interconversion thermodynamics of atropisomers.

We reached our goal, using high field NMR spectroscopy and theoretical calculations to analyze the molecular mobility of the atropisomers of **1**, obtaining results that allowed their stereochemical characterization. Actually, the agreement of the NMR outcomes with the conformations located through the modelling study led to the pursuing of the stereochemical profile of the two atropisomers a*R* and a*S* detectable in solution, useful for deepening comprehension of the drug–target interactions. 

In addition, a new chiral-phase HPLC method for controlling the synthetic steps and determining the chemical and optical purity was developed. Moreover, the solid-state structure of intermediate **7**, crystallized as a racemic compound and investigated by SC-XRD, provided insights into its overall conformation, crystal packing and molecular interactions. The structural analysis of key dihedral angles showed values in agreement with the most abundant modeled conformer **1M-A**. These outcomes contributed to shedding light on the structural determinants involved in the control of the spatial arrangement of the substituents within this molecular framework, useful for future development of derivatives with higher activity. The interest in developing small molecules orally active as GnRHR modulators is still very high because the therapies employing peptide modulators are characterized by several drawbacks (i.e., subcutaneous injection). In conclusion, the synthetic, spectroscopic, and crystallographic results of this investigation provided a valuable starting point for future studies to obtain, with minor structural modifications, separable atropisomers. These results can be extended to other new potentially active candidates to establish the relationship existing between their stereochemical features and the pharmacological properties. As atropisomers allow a predictable relative arrangement of groups in space, with those parameters in hand, we could design new strategies to address the constraints of the controlled stereoselective synthesis of atropisomers, stable at ambient temperature, offering an increasing structural diversity with application in the medicinal chemistry research field. Overall, this work demonstrated that the complete understanding of the atropisomerism phenomena is a very promising source of information for the development of drugs against a variety of diseases. Although there are still many gaps in the knowledge of these systems, the fundamental understanding of atropisomerism enables new, well-reasoned approaches to develop better therapeutic strategies.

## Figures and Tables

**Table 1:** ^1^H NMR chemical shifts (ppm) ^a^ and coupling constants (Hz) ^b^ of compounds **5**–**9** and **1**.

^1^H	5	6	7	8	9	1
1	/	/	/	/	/	/
2	/	/	/	/	/	/
3	8.97 (brs)	Exchanged with CD_3_OD	8.52 (brs)	/	/	/
4	/	/	/	/	/	/
5	5.59 (s)	/	/	/	/	/
6	/	/	/	/	/	/
7	5.36 (s)	5.38 (s)	5.47 (s)	5.50 (m)	5.47 (m)	5.43 (m)
8	/	/	/	/	/	/
9	/	/	/	/	/	/
10	7.25 (dd, *J* = 12.3 and 8.5 Hz)	7.20 (dd, *J*= 11.7 and 8.3 Hz)	7.28 (dd, *J* = 11.8 and 8.3 Hz)	7.26 (m, overlapped with 28)	7.29 (m, overlapped with 28)	7.44 (m)
11	7.42 (m)	7.37 (m)	7.42 (m)	7.41 (m, overlapped with 26 and 26’)	7.41 (m)	7.53 (td, *J* = 8.1, 5.0 Hz)
12	7.54 (d, *J* = 7.9 Hz)	7.48 (d, *J* = 7.8 Hz)	7.54 (d, *J* = 7.9 Hz)	7.55 (d, *J* = 7.9 Hz)	7.54 (d, *J* = 7.9 Hz)	7.62 (d, *J* = 7.9 Hz)
13	/	/	/	/	/	/
14	/	/	/	/	/	/
15	2.15 (s)	2.35 (s)	2.05 (s)	2.07 (s)	2.07 and 2.06 *	2.085 (s) and 2.076 (s) *
16	/	/	/	/	/	/
17	/	/	/	/	/	/
18	/	/	/	/	/	/
19	/	/	6.97 (m)	6.97 (td, *J* = 8.2 and 1.3 Hz)	6.97 (tt, *J* = 8.1 and 1.6 Hz)	7.10 (n.d., overlapped with 20)
20	/	/	7.10 (dd, *J* = 9.0 and 7.9 Hz)	7.11 (tdd, *J* = 8.0, 2.8, and 1.3 Hz)	7.11 (tdd, *J* = 8.0, 4.6, and 1.3 Hz)	7.11 and 7.14 (n.d., overlapped with 19)
21	/	/	6.81 (m)	6.85 (td, *J* = 6.2 and 1.5 Hz) and 6.78 (td, *J* = 6.2 and 1.4 Hz) *	6.83 (ddd, *J* = 7.7, 6.0, and 1.6 Hz) and 6.76 (ddd, *J* = 7.7, 6.0, and 1.6 Hz) *	6.76 (m) and 6.61 (m) *
22	/	/	3.88 (s)	3.89 (s)	3.889 (s) and 3.886 (s) *	3.883 (s) and 3.880 (s) *
23	/	/	/	4.29 (m o ddd, *J* = 16.2, 13.1, 9.8 Hz, H_a_) and 4.11 (m, H_b_)	4.28 (m, H_a_) and 4.04(m, H_b_)	4.26–4.07 (m)
24	/	/	/	4.42 (dd, *J* = 9.8 and 4.5 Hz)	4.12 (m)	4.11 (n.d.)
25	/	/	/	/	/	/
26 and 26′	/	/	/	7.41 (m, overlapped with 11)	7.37 (m)	7.29 (n.d., overlapped with 28)
27 and 27′	/	/	/	7.32 (m)	7.30 (m, overlapped with 10)	7.27 (n.d., overlapped with 26 and 26’)
28	/	/	/	7.27 (m, overlapped with 10)	7.24 (m)	7.22 (m)
29	/	/	/	2.13	1.63 (brs)	/
30	/	/	/	/	2.46 (m, H_a_) and 2.38 (m, H_b_)	2.40 (m)
31	/	/	/	/	1.68 (m)	1.75 (m, H_a_) and 1.68 (m, H_b_)
32	/	/	/	/	2.29 (m)	2.09 (m, overlapped with 15)
33	/	/	/	/	/	/
C*H_2_*CH_3_	/	/	/	/	4.084 (q, *J* = 7.1Hz,) and 4.082 (q, *J* = 7.1Hz) *	/
CH_2_C*H_3_*	/	/	/	/	1.21 (t, *J* = 7.1 Hz)	/

nd = *J*(H,H) were not determined due to the overlapping. ^a^ Assignments from ^1^H–^1^H COSY, HSQC, and HMBC data in CDCl_3_ (**5** and **7**–**9**), CD_3_OD (**1**), and CDCl_3_/CD_3_OD 9:1 mixture (**6**) at 298K. ^b^ Coupling constants were obtained by direct inspection of the spectra. Experimental error in the measured ^1^H-^1^H coupling constants was ±0.5 Hz. * The two signals are due to the presence of two atropisomers.

**Table 2:** ^13^C NMR chemical shifts (ppm) ^a^ and ^13^C-^19^F coupling constants (Hz) ^b^ data of **5**–**9** and **1**.

^13^C	5	6	7	8	9	1
1	/	/	/	/	/	/
2	151.7	150.8	151.0	152.3	152.3 and 152.2 *	153.3 and 153.2 *
3	/	/	/	/	/	/
4	162.4	159.6	161.2	161.8 and 161.6 *	161.7 and 161.6 *	163.5 and 163.3 *
5	102.5	99.2	108.8	108.3 and 108.2 *	108.3 and 108.2 *	109.3 and 109.2 *
6	153.6	151.6	151.9	149.9 and 149.8 *	149.7 and 149.6 *	152.6 and 152.5 *
7	41.1 (m)	42.5 (m)	41.8 (m)	42.7 (brs)	42.7 (brs)	44.1 (brs)
8	121.8 (d, *J* = 11.6 Hz)	121.2 (d, *J* = 11.3 Hz)	121.8 (d, *J* = 11.1 Hz)	122.0 (d, *J*= 11.4 Hz)	122.1 (d, *J* = 11.3 Hz)	123.6 (overlapped with 12 and 16)
9	161.4 (d, *J* = 250.0 Hz)	161.1 (d, *J* = 249.6 Hz)	161.4 (d, *J* = 250.0 Hz)	161.3 (d, *J* = 249.5 Hz)	161.3 (d, *J* = 250.0 Hz)	162.8 (d, *J* =248.0 Hz)
10	120.9 (d, *J* = 24.0 Hz)	120.9 (d, *J* = 24.0 Hz)	121.0 (d, *J* = 24.4 Hz)	120.9 (d, *J* = 24.1 Hz)	120.9 (d, *J* = 24.4 Hz)	122.15 (d, *J* = 24.3 Hz) and 122.19 (d, *J* = 24.3 Hz) *
11	129.5 (d, *J* = 10.0 Hz)	129.6 (d, *J* = 9.7 Hz)	129.5 (d, *J* = 9.8 Hz)	129.3 (d, *J* = 9.8 Hz)	129.3 (d, *J* = 9.6 Hz)	130.9 and 130.8 *
12	122.6 (m)	122.5 (m)	122.6 (m)	122.6 (m)	122.5 (m)	123.7 (overlapped with 8 and 16)
13	129.6 (dd, *J* = 30.0 and 3.6 Hz; partially hidden by 11)	129.3 (dd, *J* = 30.9 and 3.7 Hz;	129.6 (dd, *J* = 30.9 and 4.0 Hz)	129.5 (dd, *J* = 31.0 and 3.1, partially overlapped with 11)	129.3 (dd, *J* = 30.2 and 3.0 Hz)	130.6 (dd, *J* = 31.1 and 3.3 Hz)
14	123.4 (dd, *J* = 273.6 and 4.0 Hz)	123.3 (dd, *J* = 274.3 and 4.2 Hz)	123.5 (dd, *J* = 273.8 and 3.9 Hz)	123.5 (dd, *J* = 273.6 and 3.8 Hz)	123.5 (dd, *J* = 271.1 and 3.7 Hz)	125.1 (dd, *J* = 273.3 and 3.8 Hz)
15	20.1	20.1	17.9	17.8	17.8	18.1
16	/	/	121.5 (d, *J* = 13.6 Hz)	122.3 (d, *J* = 13.7 Hz) and 122.2 (d, *J* = 13.7 Hz) *	122.37 (d, *J* = 13.8 Hz) and 122.35 (d, *J* = 13.8 Hz)	123.8 (overlapped with 8 and 12)
17	/	/	149.9 (d, *J* = 246.4 Hz)	150.2 (d, *J* = 246.3 Hz) and 150.1 (d, *J* = 246.3 Hz) *	150.23 (d, *J* = 245.5) and 150.17 (d, *J* = 245.5)	151.8 (d, *J* = 245.4 Hz)
18	/	/	148.0 (d, *J* = 11.0 Hz)	148.0 (d, *J* = 11.0 Hz)	148.0 (d, *J* = 11.0 Hz)	149.5 (d, *J* = 11.2 Hz)
19	/	/	113.5	113.3	113.2	114.8
20	/	/	123.8	123.8 (overlapped with 21)	123.80 (s) and 123.76 (s) *	125.1 (s) and 125.0 (s) *
21	/	/	123.9 (d, *J* = 4.6 Hz)	123.9 and 123.8 (overlapped with 20) *	123.92 (s) and 123.85 (s) *	125.0 and 124.9 *
22	/	/	56.3	56.2	56.2	56.8
23	/	/	/	49.10 and 49.14 *	47.7 and 47.6 *	48.1 and 47.9 *
24	/	/	/	54.1 and 54.2 *	60.9 and 60.8 *	62.2 and 62.0 *
25	/	/	/	143.7 and 143.6 *	141.92 (s) and 141.89 (s) *	142.1 and 141.9 *
26 and 26’	/	/	/	126.4 and 126.3 *	127.2 and 127.1 (overlapped with 28) *	128.7
27 and 27’	/	/	/	128.5 and 128.4 *	128.4 and 128.3 *	129.4
28	/	/	/	127.3 and 127.2 *	127.2 (overlapped with 26 and 26′)	128.5
29	/	/	/	/	/	/
30	/	/	/	/	46.51 (s) and 46.47 (s) *	48.7
31	/	/		/	25.4	27.86 (s) and 27.83 (s) *
32	/	/	/	/	32.0	37.03 (s) and 37.00 (s) *
33	/	/	/	/	173.7 60.1	182.3
*C*H_2_CH_3_	/	/	/	/		/
CH_2_*C*H_3_	/	/	/	/	14.2	/

^a^ Assignments from HSQC and HMBC data in CDCl_3_ (**5** and **7**–**9**), CD_3_OD (**1**), and CDCl_3_/CD_3_OD 9:1 mixture (**6**) at 298K. ^b^ Coupling constants were obtained by direct inspection of the spectra. Experimental error in the measured ^1^H-^1^H coupling constants was ±0.5 Hz. * The two signals are due to the presence of two atropisomers.

**Table 3:** ^15^N NMR chemical shifts (ppm) ^a^ of compounds **5**–**9** and **1**.

^15^N	5	6	7	8	9	1
1	136.7	137.9	134.3	134.3	135.5	137.1
3	154.3 ^b^	153.4 ^b^	151.9 ^b^	160.1	161.3	160.5
29	/	/	/	31.2	43.6	46.3

^a^ Assignments from ^1^H-^15^N HMBC data in CDCl_3_ (**5** and **7**–**9**), CD_3_OD (**1**), and CDCl_3_/CD_3_OD 9:1 mixture (**6**) at 298K using nitromethane as external reference (neat nitromethane = 381.7 ppm as reported in Ref. [32]). ^b^ Assignments from ^1^H-^15^N HSQC data in CDCl_3_ (**5** and **7**), and CDCl_3_ + 3% of DMSO-d_6_ (**6**) at 298K using ammonia as external reference (ammonia = 0.0 ppm as reported in Ref. [32]).

**Table 4:** ^1^H-^13^C gs-HMBC long range couplings observed in the spectra of **1**.

^13^C	HMBC (^13^C→^1^H)
C-2	H-7 and H-23
C-4	H-23 and H-15 (weak)
C-5	H-21 and H-15
C-6	H-15
C-8	H-7, H-10, and H-12
C-9	H-7, H-10, and H-11
C-13	H-7 and H-11
C-14	H-12
C-16	H-20
C-17	H-19 and H-21
C-18	H-20 and H-22
C-25	H-23, H-24, H-27, and H-27’
C-33	H-31 and H-32

**Table 5:** Geometrical features, relative energy values and equilibrium percentages of the predicted conformations in the gas phase, as resulting after geometry optimization.

Conformers	τ_1_ (°)	τ_2_ (°)	τ_3_ (°)	τ_4_ (°)	Gas Phase ΔE (kcal/mol)	Gas Phase (%)
**1A**	−81.6	−48.5	−132.0	49.1	4.35	0.0
**1B**	−70.0	147.8	−130.0	49.7	6.84	0.0
**1C**	96.0	−54.0	−125.0	70.0	0.67	20.5
**1D**	111.8	134.3	−133.0	52.5	4.60	0.0
**1E**	−80.5	−47.0	−61.3	−52.4	4.67	0.0
**1F**	−69.4	150.3	−55.0	−55.5	7.09	0.0
**1G**	113.0	136.0	−60.5	−55.6	5.01	0.0
**1H**	93.2	−62.1	−64.1	−55.6	1.90	2.6
**1I**	94.5	−53.6	61.3	−142.6	2.26	1.4
**1J**	118.5	147.4	59.4	−140.8	4.86	0.0
**1K**	−74.0	−40.3	67.8	−141.2	4.78	0.0
**1L**	−69.1	148.9	60.5	−142.9	3.69	0.1
**1M**	92.6	−61.9	−110.0	−134.5	0.00	63.9
**1N**	113.6	146.5	−77.5	147.4	3.98	0.1
**1O**	−85.9	−51.6	−115.4	−136.4	3.58	0.2
**1P**	−70.9	148.7	−75.9	146.3	5.33	0.0
**1Q**	−81.0	−46.4	−75.4	142.8	3.64	0.1
**1R**	−71.1	148.9	−75.2	147.0	5.40	0.0
**1S**	92.7	−55.0	−76.1	139.9	1.09	10.1
**1T**	113.9	146.9	−77.6	147.5	3.93	0.1
**1U**	−86.3	−49.9	56.0	53.0	4.53	0.0
**1V**	−70.1	145.9	51.1	55.2	3.67	0.1
**1W**	93.5	−53.5	49.9	53.8	2.77	0.6
**1X**	114.2	142.7	51.3	53.6	5.32	0.0

**Table 6:** Geometrical features, relative energies and equilibrium percentages after geometry optimization of the most populated conformations of a simplified structure of **1**.

Code	τ_1_	τ_2_	τ_3_	τ_4_	τ_5_	Water ΔE (kcal/mol)	Water (%)
**7** **(crystal structure)**			−105.5	−144.7	110.4		
**1M−A**	87.7°	−63.3°	−104.7°	−133.9°	−111.8°	0.00	83.6
**1M−C**	91.3°	−60.2°	−106.7°	−132.6°	75.2°	2.07	2.6
**1M−D**	92.9°	−60.3°	−110.0°	−135.0°	126.3°	1.06	13.8

**Table 7:** Geometrical features, relative energies and equilibrium percentages after geometry optimization of the most populated conformations of **1M-A** and **1M-D**, created rotating the dihedral angle involving in the methoxy group (τ6).

Conformer	t_6_	Distance C_H22_ – H19 (Å)	Gas Phase ΔE (kcal/mol)	Gas Phase (%)	Methanol ΔE (kcal/mol)	Methanol (%)
**1M-A**	0	2.56	0.00	44.5	0	86.0
**1M-A (I)**	−66	3.60	0.29	27.5	1.62	5.6
**1M-A (II)**	+66	3.60	0.27	28.0	1.38	8.4
**1M-D**	0	2.56	0.22	30.5	0.00	84.5
**1M-D (I)**	−66	3.60	0.32	25.6	1.36	8.5
**1M-D (II)**	+66	3.60	0.00	43.9	1.48	7.0

**Table 8:** Crystal data and structural parameters of **7**.

Crystal Data	
Chemical formula	C_20_H_15_F_5_N_2_O_3_
*M*_r_	426.34
Crystal system, space group	Orthorhombic, P bca
*a*, *b*, *c* (Å)	11.165 (2), 11.073 (2), 30.367(6)
*V* (Å^3^)	3754.2(13))
*Z*	8
*F* (000)	1744
Density (g/cm^3^)	1.509
Temperature (K)	298
Radiation type	Mo-Kα (*λ* = 0.71073 Å)
*µ* (mm^−1^)	1.135
Crystal size (mm)	0.06 × 0.03 × 0.02
**Data collection**	
Diffractometer	Bruker-Axs Smart-Apex CCD
*T*_min_, *T*_max_	0.893, 1.000
No. of measured, independent and observed [*I* > 2σ(*I*)] reflections	29,138, 4175, 1715
*R*_int_	0.0298
**Structure refinement**	
*R*, *wR^2^*, *S*	0.0437 (I > 2σ(I)) and 0.1119 (all), 0.0973 (I > 2σ(I)) and 0.1126 (all), 0.760 (all)
No. of parameters	271
No. of restraints	0

## Data Availability

Not applicable.

## Supplementary Materials

The following supporting information can be downloaded at: https://www.mdpi.com/article/10.3390/molecules28093861/s1, Figure S1: ^1^H NMR of compound **5**, Figure S2: ^13^C NMR of compound **5**, Figure S3: COSY of compound **5**, Figure S4: ^1^H-^13^C HSQC of compound **5**, Figure S5: ^1^H-^13^C HMBC of compound **5**, Figure S6: ^1^H-^15^N HMBC of compound **5**, Figure S7: ^1^H-^15^N HSQC of compound **5**, Figure S8: ^1^H NMR of compound **6**, Figure S9: ^13^C NMR of compound **6**, Figure S10: COSY of compound **6**, Figure S11: ^1^H-^13^C HSQC of compound **6**, Figure S12: ^1^H-^13^C HMBC of compound **6**, Figure S13: ^1^H-^15^N HMBC of compound **6**, Figure S14: ^1^H-^15^N HSQC of compound **6**, Figure S15: ^1^H NMR of compound **7**, Figure S16: ^13^C NMR of compound **7**, Figure S17: COSY of compound **7**, Figure S18: ^1^H-^13^C HSQC of compound **7**, Figure S19: ^1^H-^13^C HMBC of compound **7**, Figure S20: ^1^H-^15^N HMBC of compound **7**, Figure S21: ^1^H-^15^N HSQC of compound **7**, Figure S22: ^1^H NMR of compound **8**, Figure S23: ^13^C NMR of compound **8**, Figure S24: COSY of compound **8**, Figure S25: ^1^H-^13^C HSQC of compound **8**, Figure S26: ^1^H-^13^C HMBC of compound **8**, Figure S27: ^1^H-^15^N HMBC of compound **8**, Figure S28: ^1^H NMR of compound **9**, Figure S29: ^13^C NMR of compound **9**, Figure S30: COSY of compound **9**, Figure S31: ^1^H-^13^C HSQC of compound **9**, Figure S32: ^1^H-^13^C HMBC of compound **9**, Figure S33: ^1^H-^15^N HMBC of compound **9**, Figure S34: ^1^H NMR of compound **1**, Figure S35: ^13^C NMR of compound **1**, Figure S36: COSY of compound **1**, Figure S37: ^1^H-^13^C HSQC of compound **1**, Figure S38: ^1^H-^13^C HMBC of compound **1**, Figure S39: ^1^H-^15^N HMBC of compound **1**, Figure S40: NOESY of compound **1**, Figure S41: ^1^H NMR of compound **1** in DMSO-d_6_, Figure S42: ^1^H NMR of compound **1** in D_2_O, Figure S43: ^1^H NMR of compound **11**, Figure S44: COSY of compound **11**, Table S1: Relative energies and equilibrium percentages of the optimized conformations endowed with ΔE < 5 kcal/mol in the gas phase, in water and methanol solvent models. Table S2: Internuclear H-15/H-21 distance estimated by 2D NOESY experiments.

## Sample Availability

Samples of all compounds mentioned in this manuscript are available from the authors.
