# Supplementary material for: Elagolix Sodium Salt and Its Synthetic Intermediates: A Spectroscopic, Crystallographic, and Conformational Study
Source: Molecules. 2023 May 3;28(9):3861. doi: 10.3390/molecules28093861 (PMC10180344; doi:10.3390/molecules28093861)
Supplement: Supplementary file 1 [file molecules-28-03861-s001.zip › molecules-2353843-supplementary.pdf]

Elagolix Sodium Salt and Its Synthetic Intermediates: A Spec-troscopic, Crystallographic, and Conformational Study

Samuele Ciceri [1,2], Diego Colombo [2], Enrico M. A. Fassi [1], Patrizia Ferraboschi [2], Giovanni Grazioso [1], Paride Grisenti [3], Marco Iannone [4], Carlo Castellano [5] and Fiorella Meneghetti [1,*]

1. Department of Pharmaceutical Sciences, University of Milan, Via L. Mangiagalli 25, 20133 Milano, Italy; samuele.ciceri@unimi.it (S.C.); enrico.fassi@unimi.it (E.M.A.F.); giovanni.grazioso@unimi.it (G.G.)
2. Department of Medical Biotechnology and Translational Medicine, University of Milan, Via C. Saldini 50, 20133 Milano, Italy; diego.colombo@unimi.it (D.C.); patrizia.ferraboschi@unimi.it (P.F.)
3. Chemical-Pharmaceutical Consulting and IP Management, Viale G. da Cermenate 58, 20141 Milano, Italy; grisenti.paride60@gmail.com
4. Tecnomed Foundation, University of Milano-Bicocca, Via Pergolesi 33, 20900 Monza, Italy; marco.iannone@unimib.it
5. Department of Chemistry, Università degli Studi di Milano, via Golgi 19, 20133 Milano, Italy; carlo.castellano@unimi.it

* Correspondence: fiorella.meneghetti@unimi.it; Tel.: +39-02-50319306

INDEX

**Figure S1**	^{1}H NMR of compound **5**
**Figure S2**	^{13}C NMR of compound **5**
**Figure S3**	COSY of compound **5**
**Figure S4**	^{1}H-^{13}C HSQC of compound **5**
**Figure S5**	^{1}H-^{13}C HMBC of compound **5**
**Figure S6**	^{1}H-^{15}N HMBC of compound **5**
**Figure S7**	^{1}H-^{15}N HSQC of compound **5**
**Figure S8**	^{1}H NMR of compound **6**
**Figure S9**	^{13}C NMR of compound **6**
**Figure S10**	COSY of compound **6**
**Figure S11**	^{1}H-^{13}C HSQC of compound **6**
**Figure S12**	^{1}H-^{13}C HMBC of compound **6**
**Figure S13**	^{1}H-^{15}N HMBC of compound **6**
**Figure S14**	^{1}H-^{15}N HSQC of compound **6**
**Figure S15**	^{1}H NMR of compound **7**
**Figure S16**	^{13}C NMR of compound **7**
**Figure S17**	COSY of compound **7**
**Figure S18**	^{1}H-^{13}C HSQC of compound **7**
**Figure S19**	^{1}H-^{13}C HMBC of compound **7**
**Figure S20**	^{1}H-^{15}N HMBC of compound **7**
**Figure S21**	^{1}H-^{15}N HSQC of compound **7**
**Figure S22**	^{1}H NMR of compound **8**
**Figure S23**	^{13}C NMR of compound **8**
**Figure S24**	COSY of compound **8**
**Figure S25**	^{1}H-^{13}C HSQC of compound **8**
**Figure S26**	^{1}H-^{13}C HMBC of compound **8**
**Figure S27**	^{1}H-^{15}N HMBC of compound **8**
**Figure S28**	^{1}H NMR of compound **9**
**Figure S29**	^{13}C NMR of compound **9**
**Figure S30**	COSY of compound **9**
**Figure S31**	^{1}H-^{13}C HSQC of compound **9**
**Figure S32**	^{1}H-^{13}C HMBC of compound **9**
**Figure S33**	^{1}H-^{15}N HMBC of compound **9**
**Figure S34**	^{1}H NMR of compound **1**
**Figure S35**	^{13}C NMR of compound **1**
**Figure S36**	COSY of compound **1**
**Figure S37**	^{1}H-^{13}C HSQC of compound **1**
**Figure S38**	^{1}H-^{13}C HMBC of compound **1**
**Figure S39**	^{1}H-^{15}N HMBC of compound **1**
**Figure S40**	NOESY of compound **1**
**Figure S41**	^{1}H NMR of compound **1** in DMSO-d_6
**Figure S42**	^{1}H NMR of compound **1** in D_2O
**Figure S43**	^{1}H NMR of compound **11**
**Figure S44**	COSY of compound **11**
**Table S1**	Relative energies and equilibrium percentages of the optimized conformations endowed with $\Delta E < 5$ kcal/mol in gas phase, in water and methanol solvent models.
**Table S2**	Internuclear H-15/H-21 distance estimated by 2D NOESY experiment.

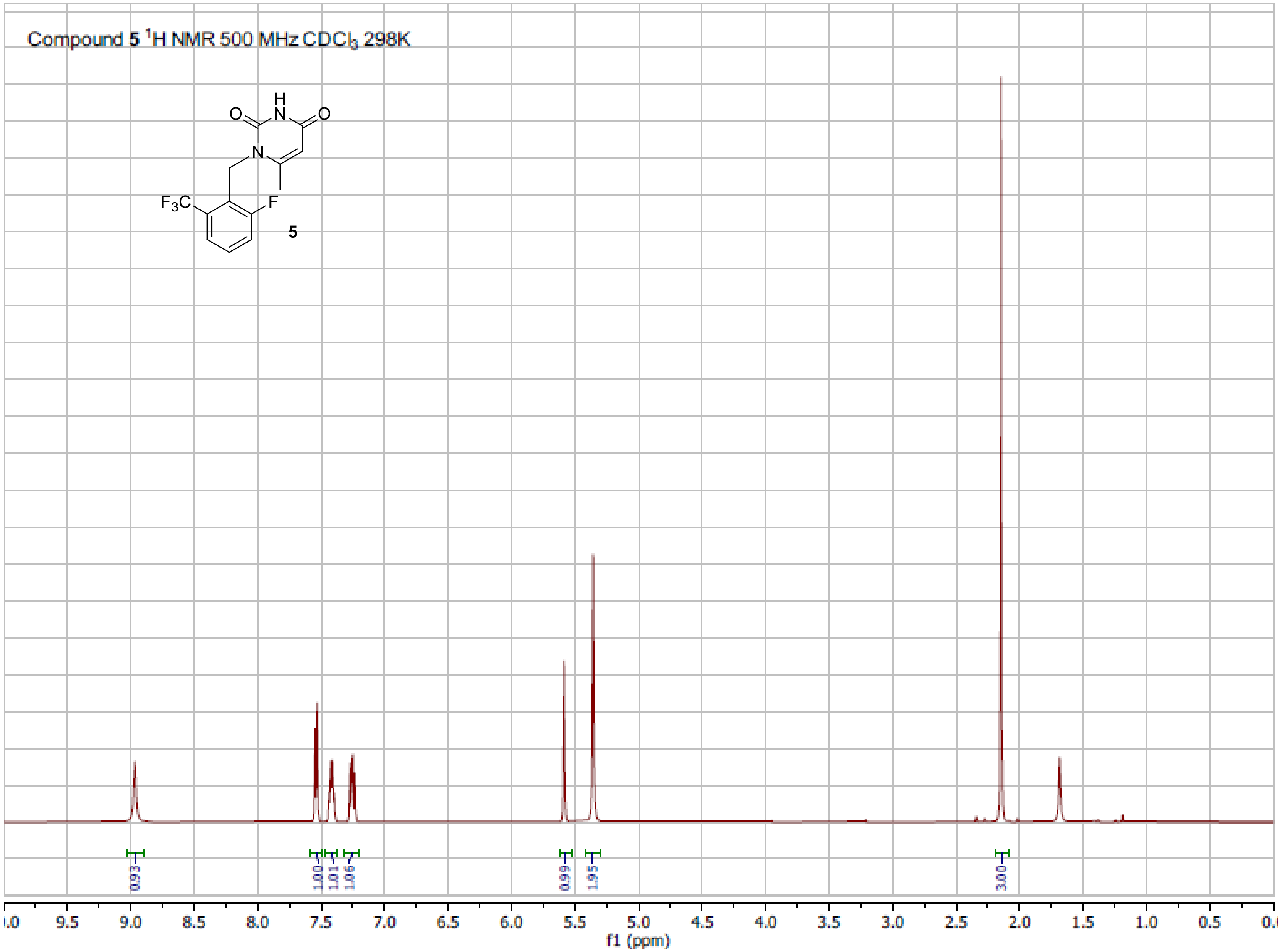

Figure S1. ¹H NMR of compound **5**

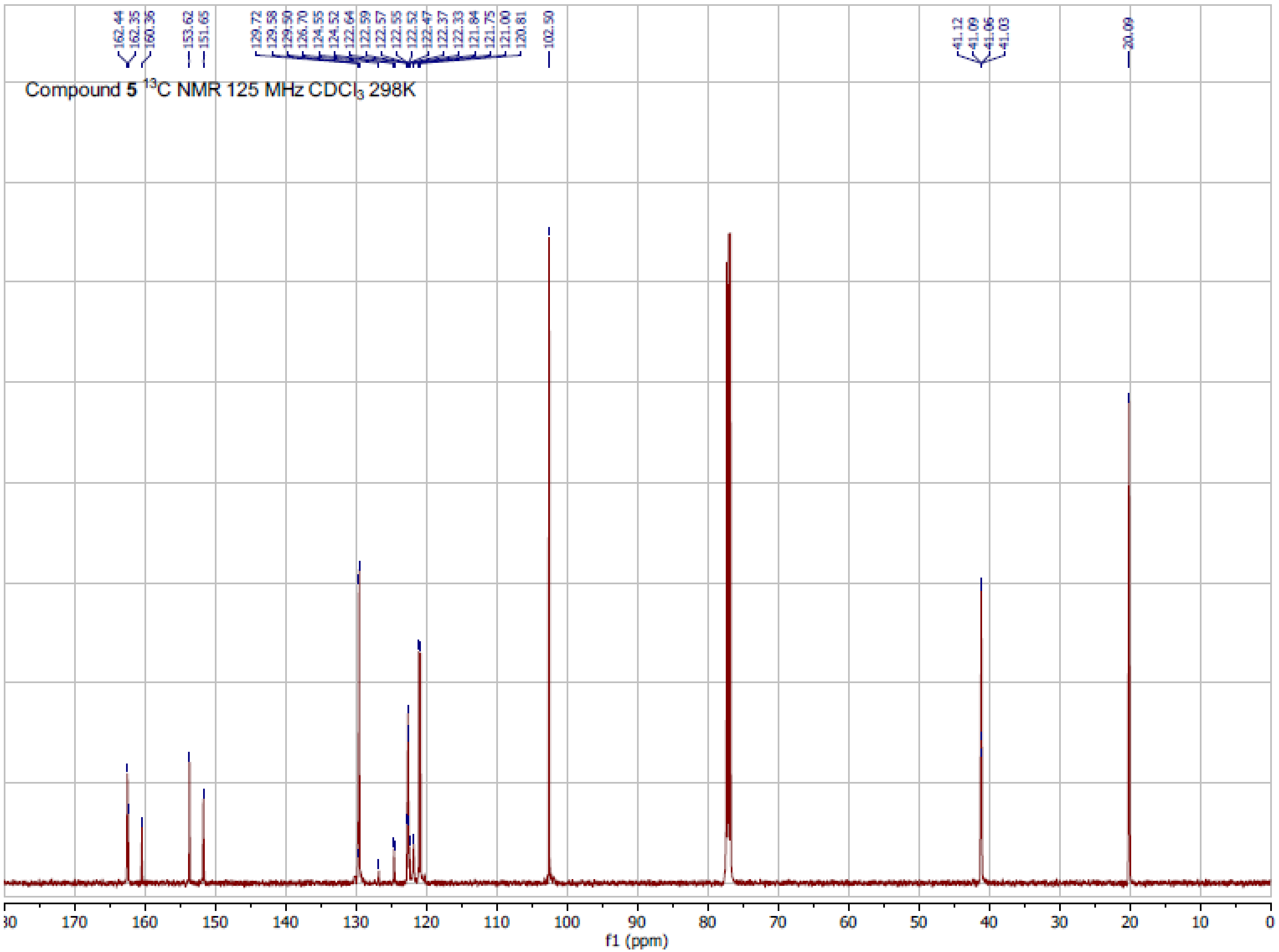

Figure S2. ^{13}C NMR of compound **5**

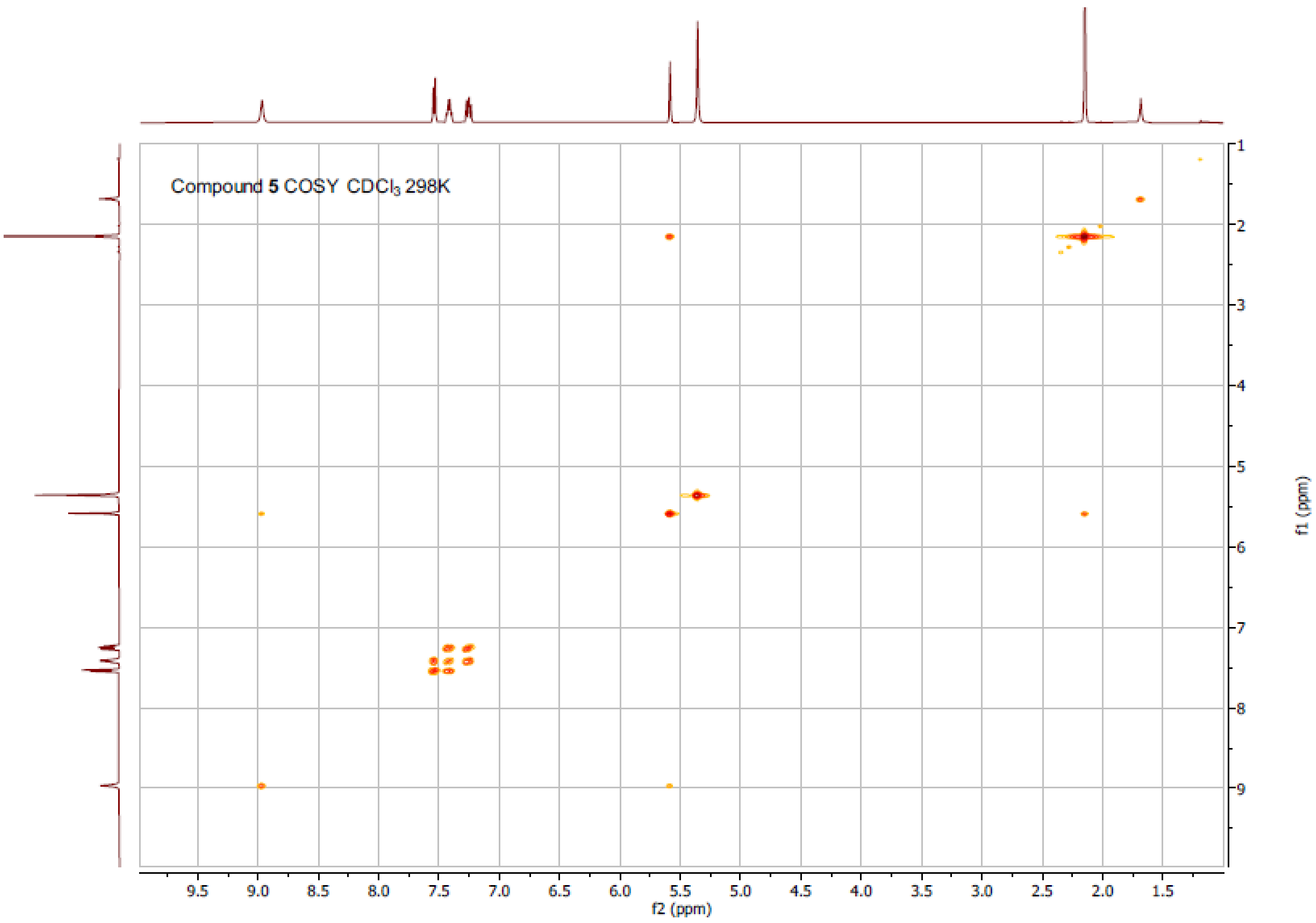

Figure S3. COSY of compound **5**

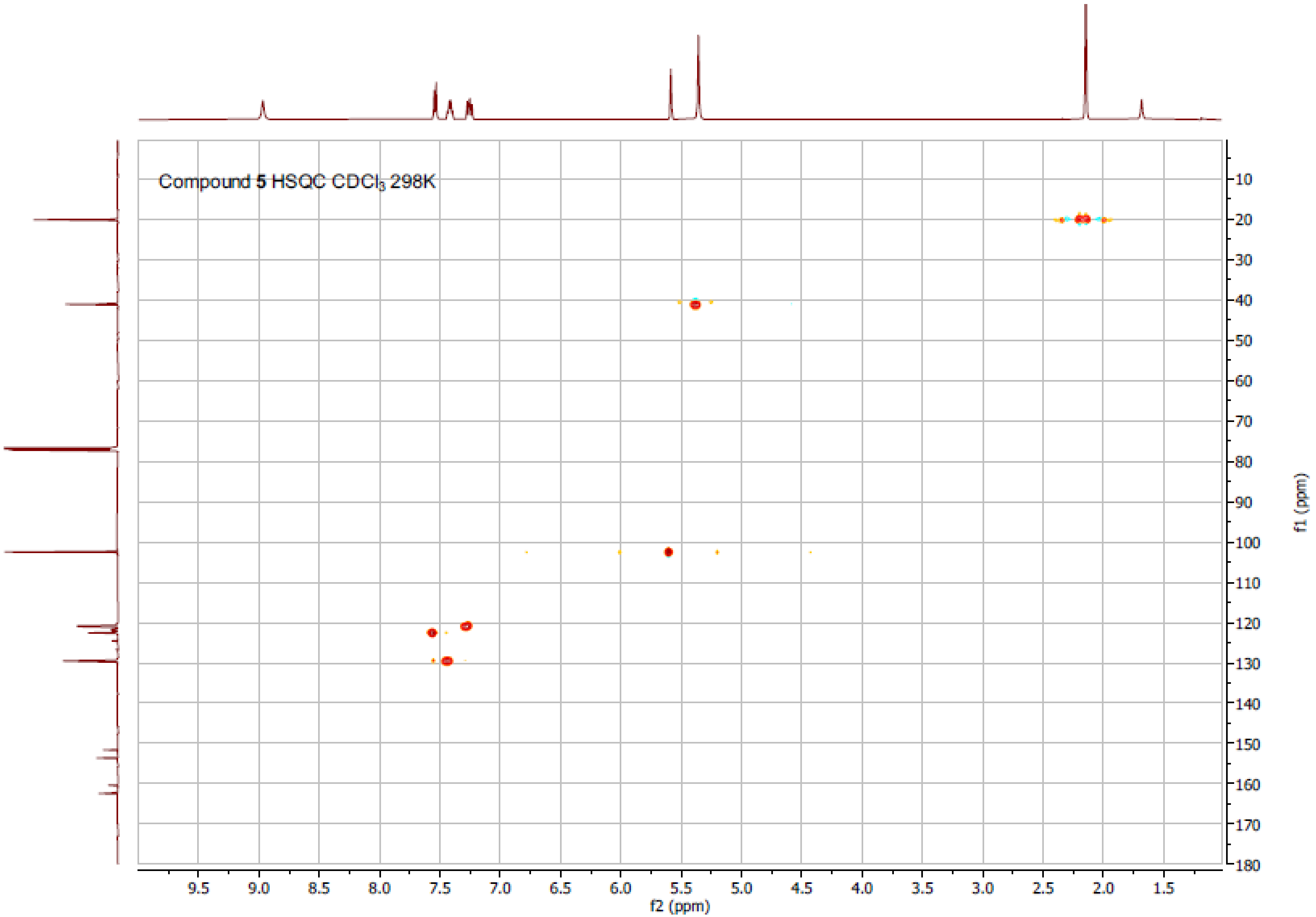

Figure S4. ^{1}H-^{13}C HSQC of compound **5**

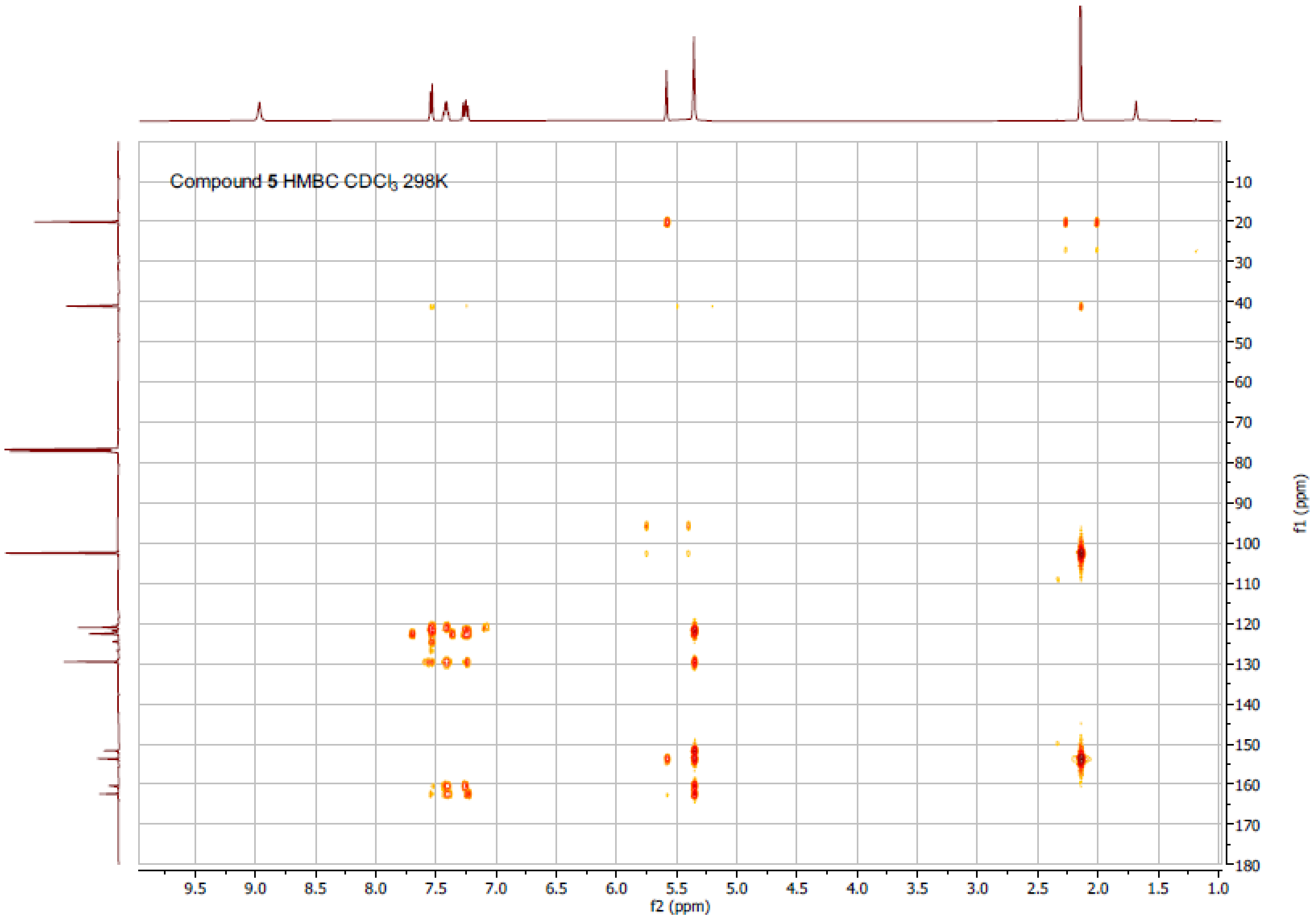

Figure S5. ^{1}H-^{13}C HMBC of compound **5**

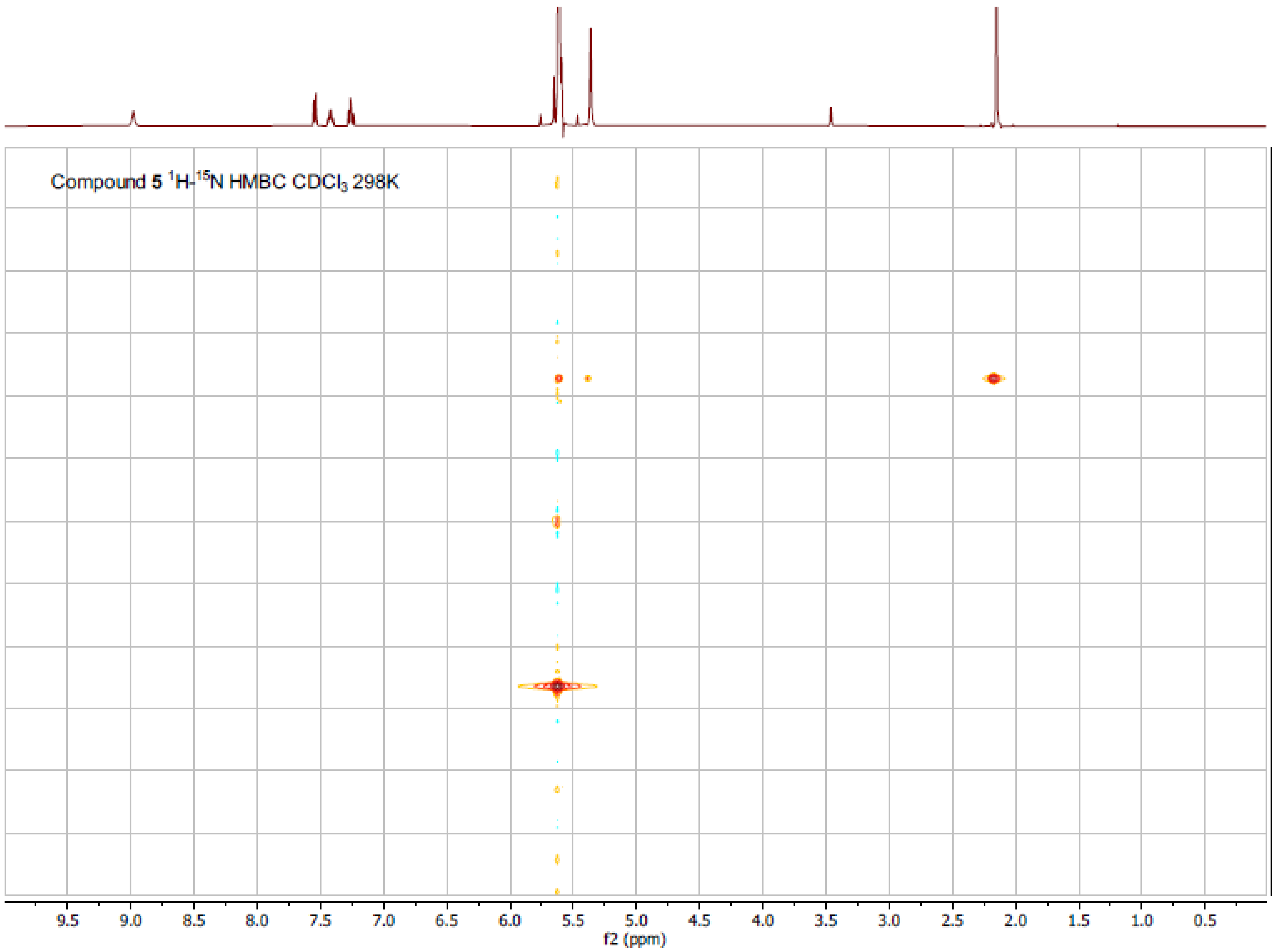

Figure S6. ^{1}H-^{15}N HMBC of compound **5**

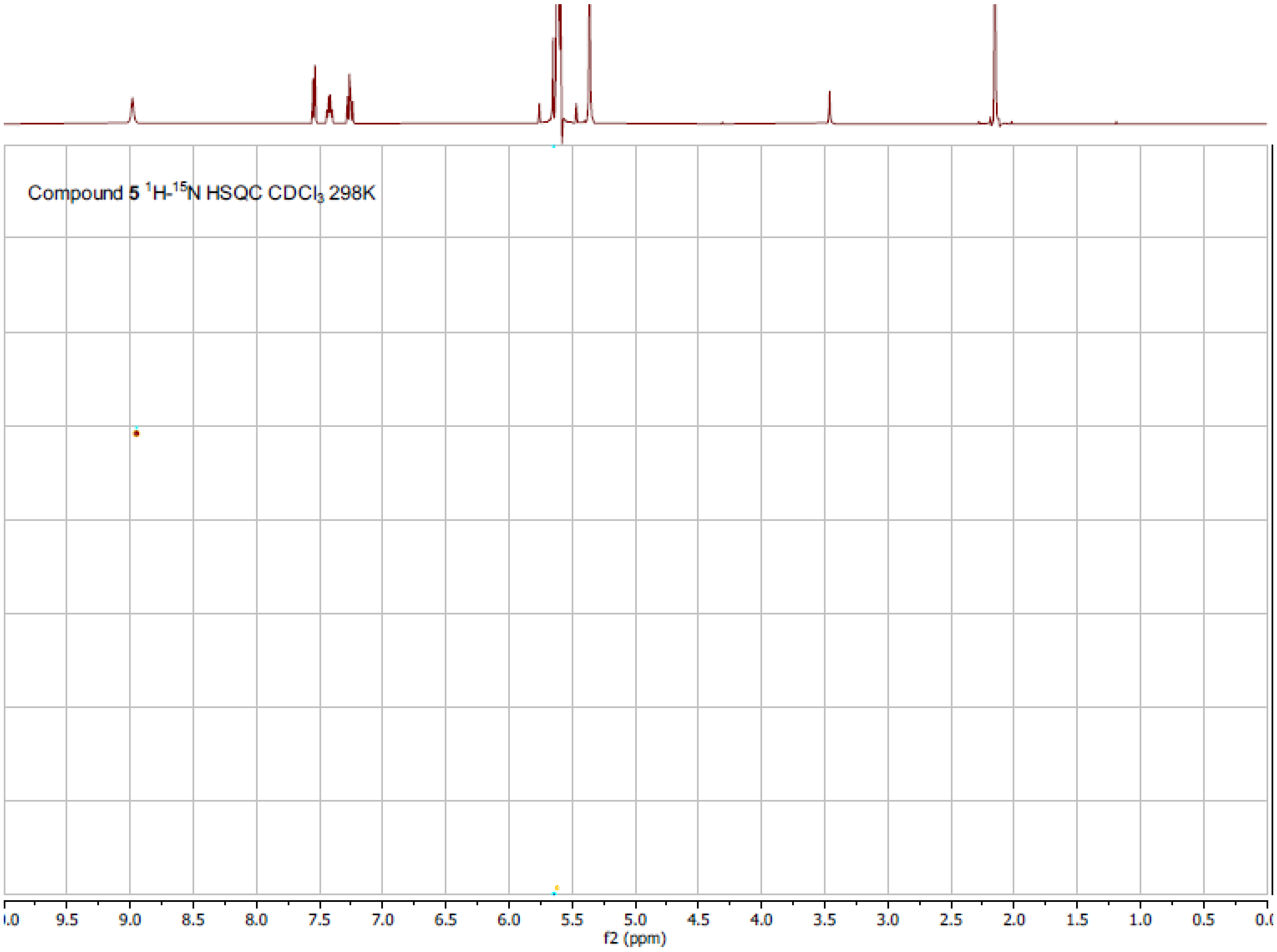

Figure S7. ^{1}H-^{15}N HSQC of compound **5**

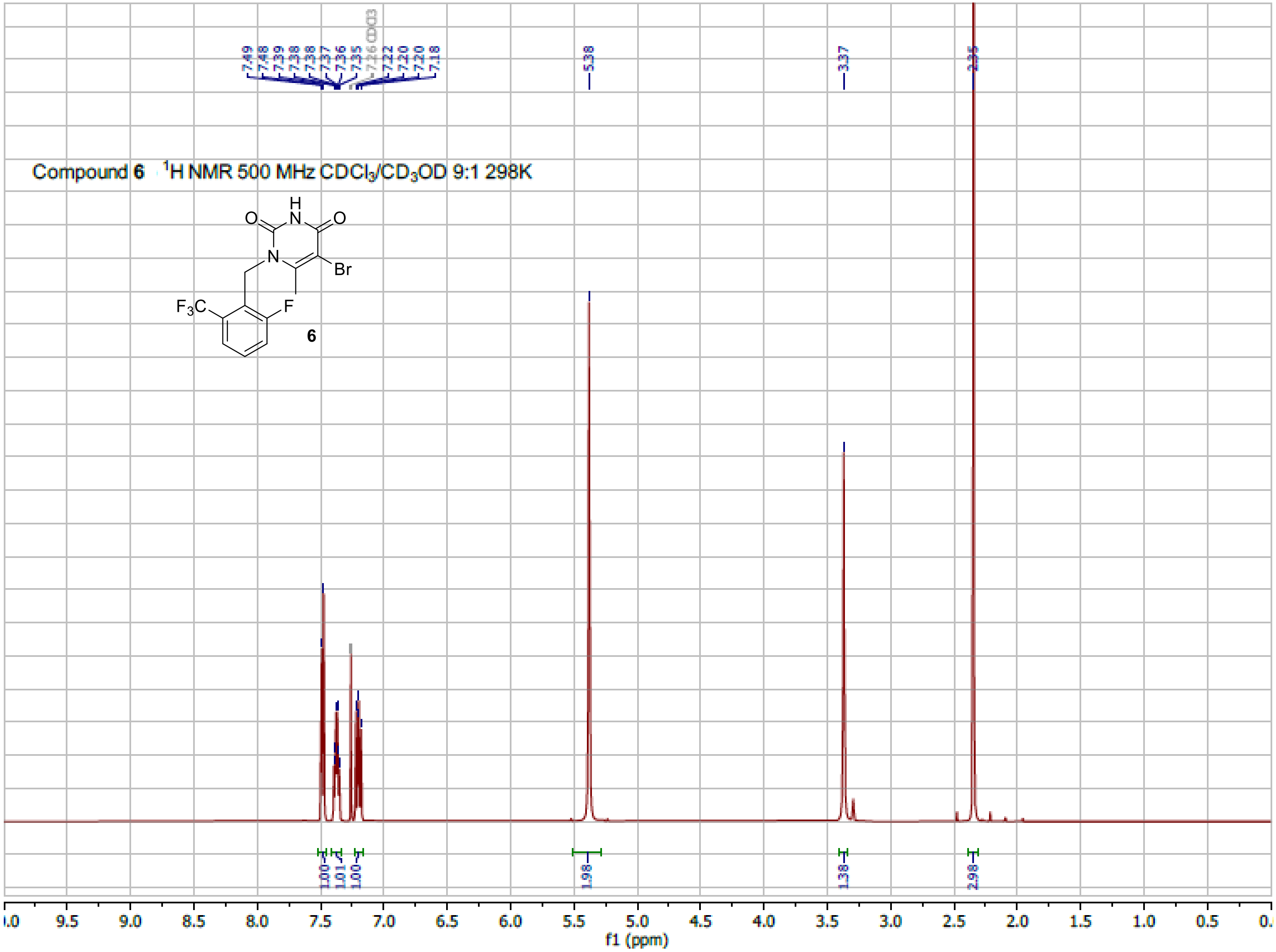

Figure S8. ¹H NMR of compound **6**

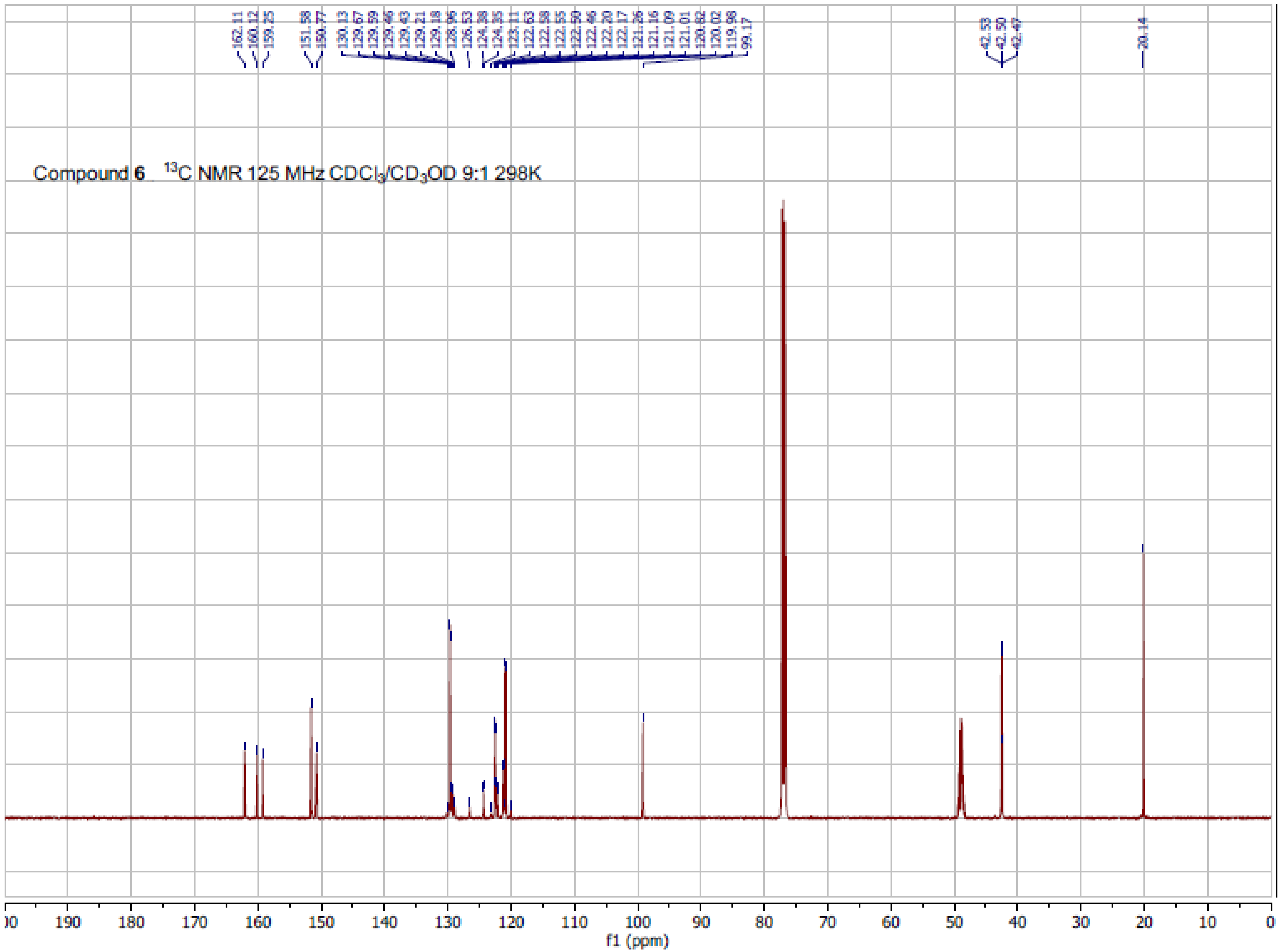

Figure S9. ^{13}C NMR of compound **6**

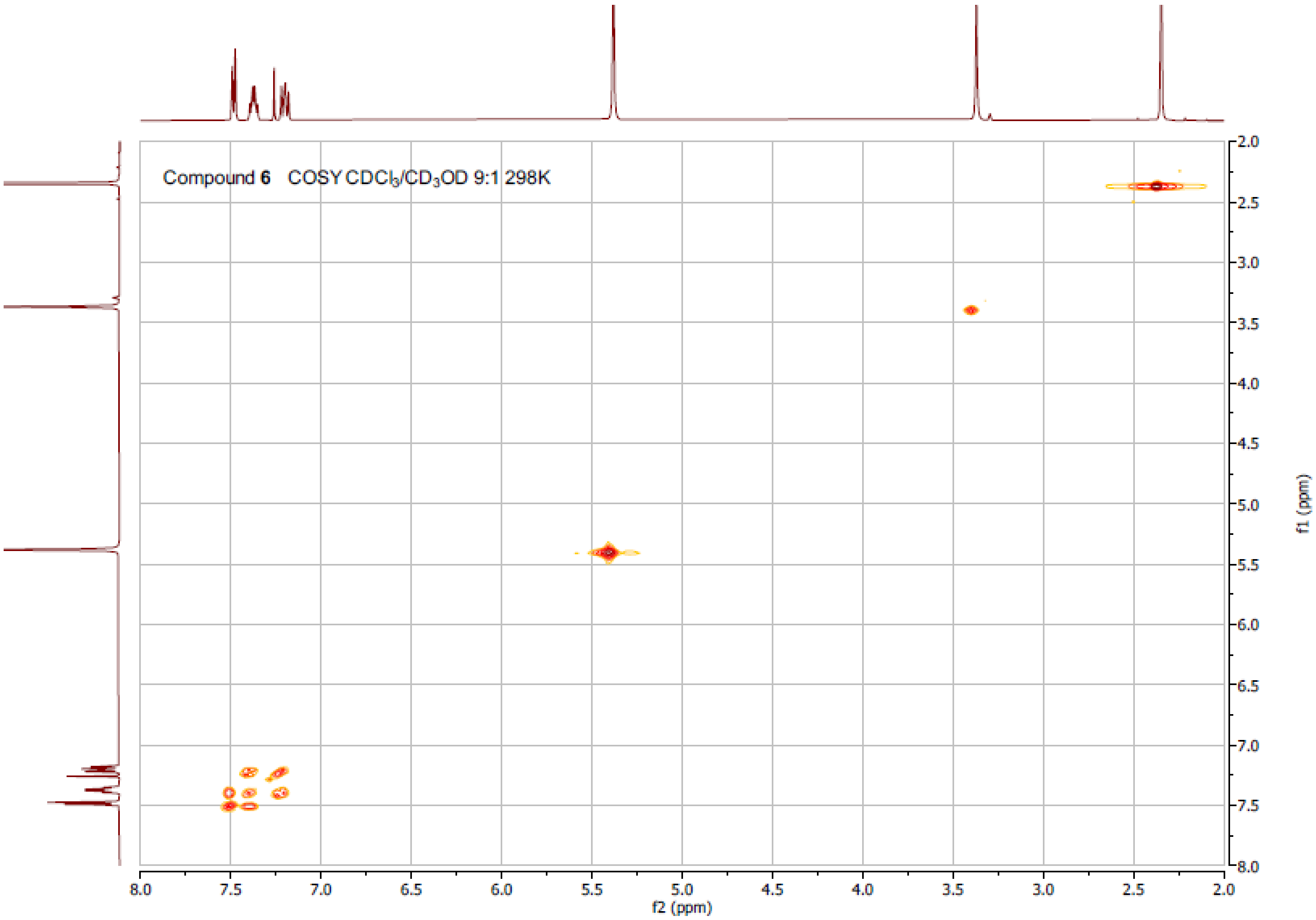

Figure S10. COSY of compound **6**

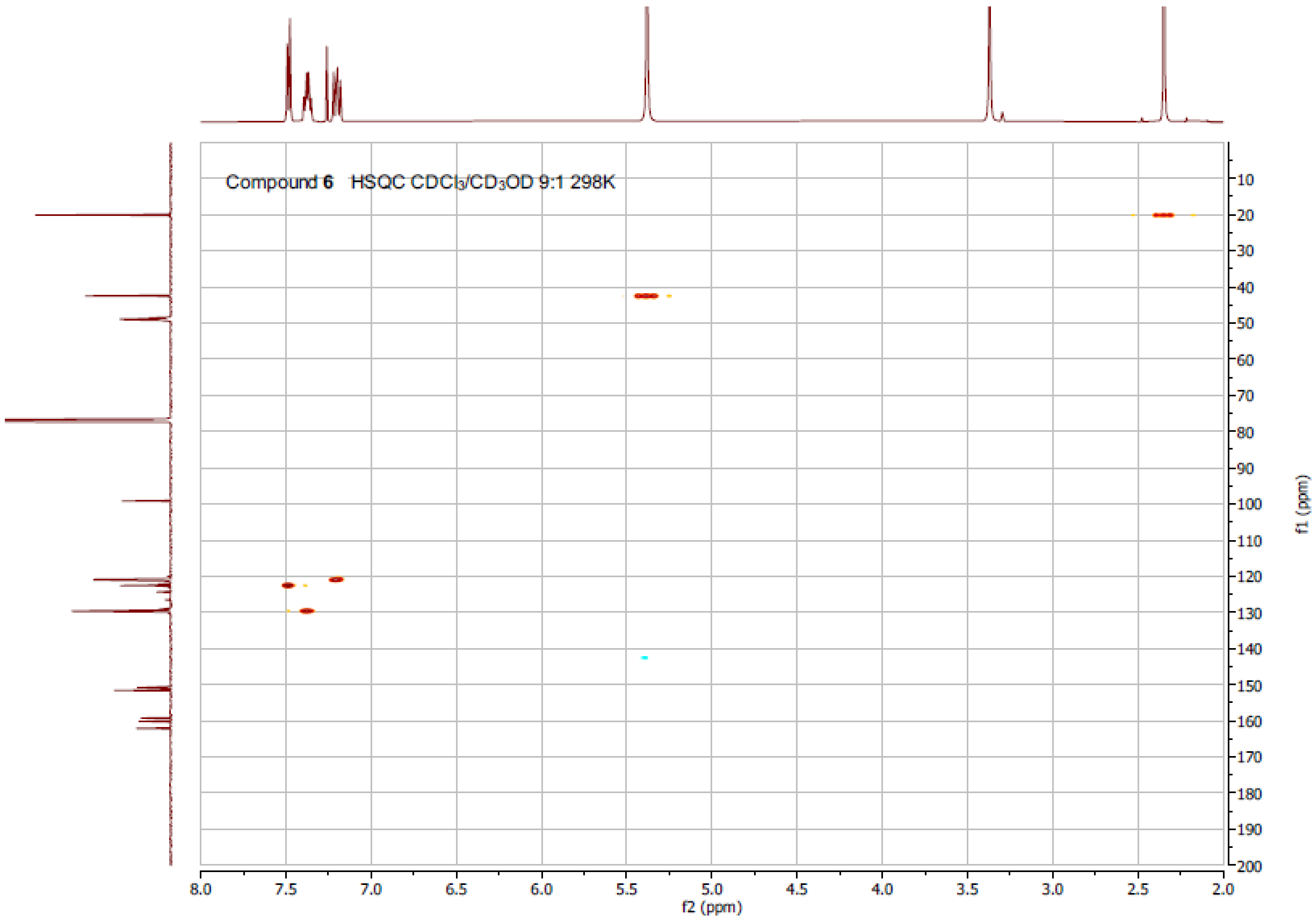

Figure S11. ^{1}H-^{13}C HSQC of compound **6**

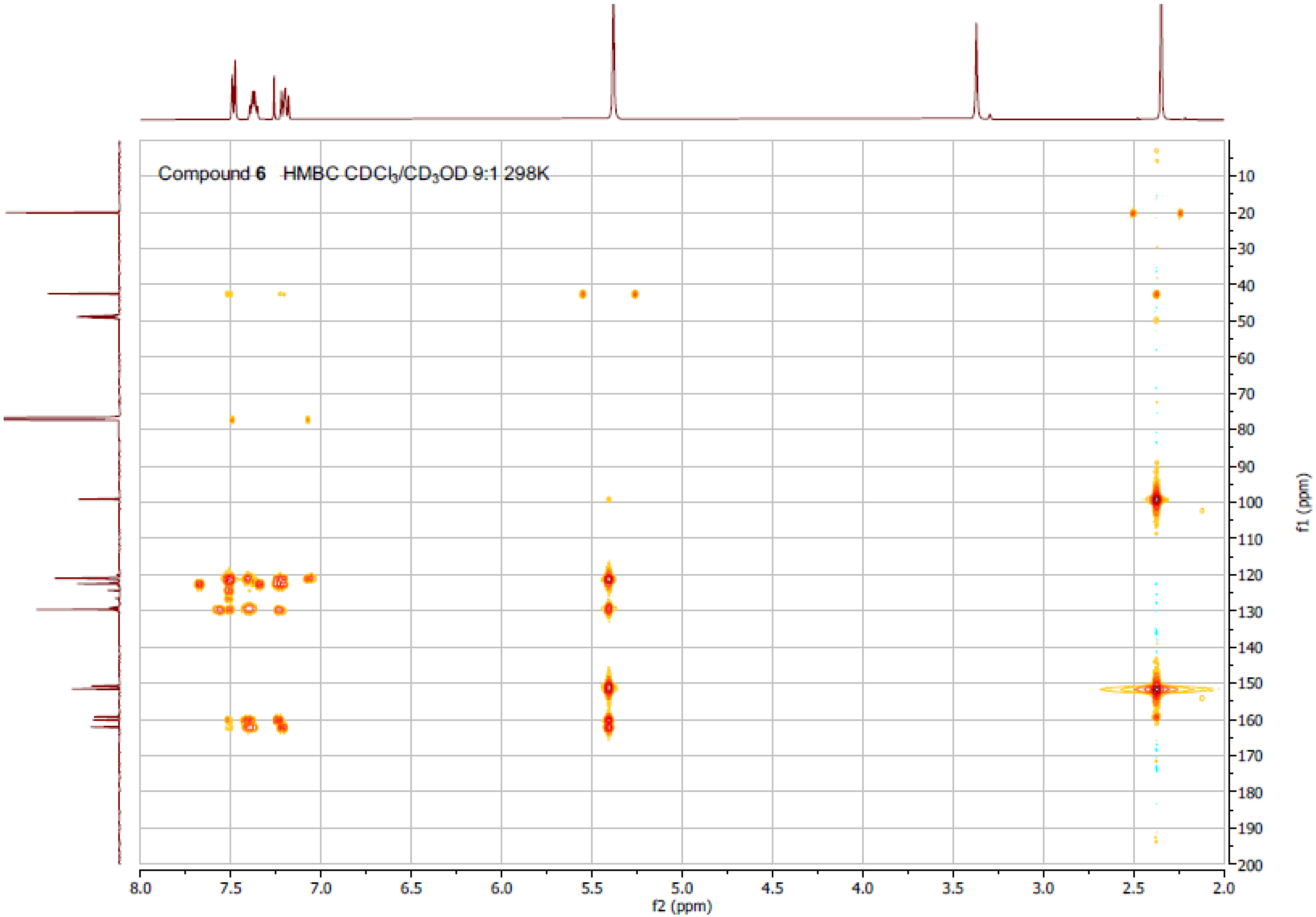

Figure S12. ^{1}H-^{13}C HMBC of compound **6**

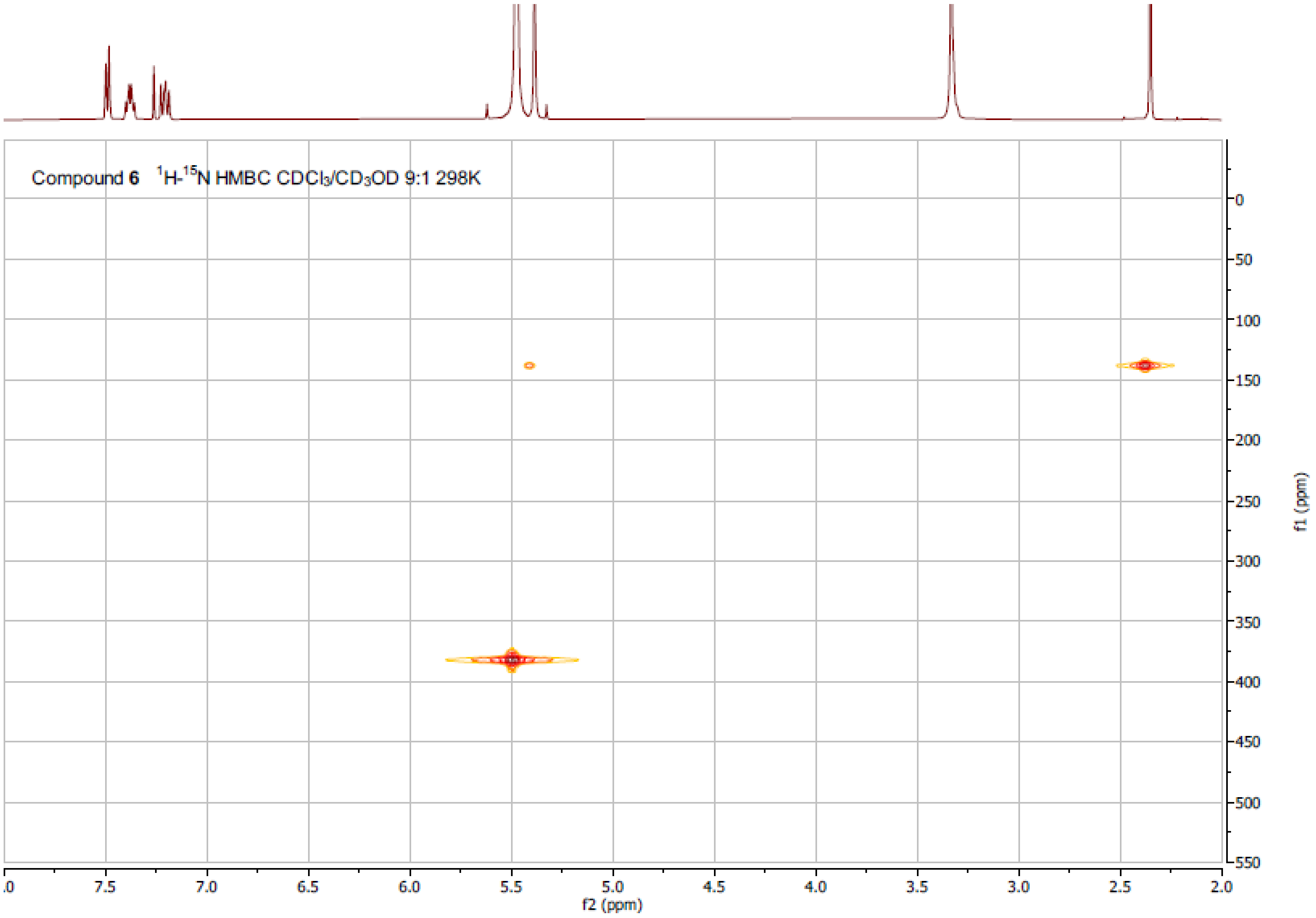

Figure S13. ^{1}H-^{15}N HMBC of compound **6**

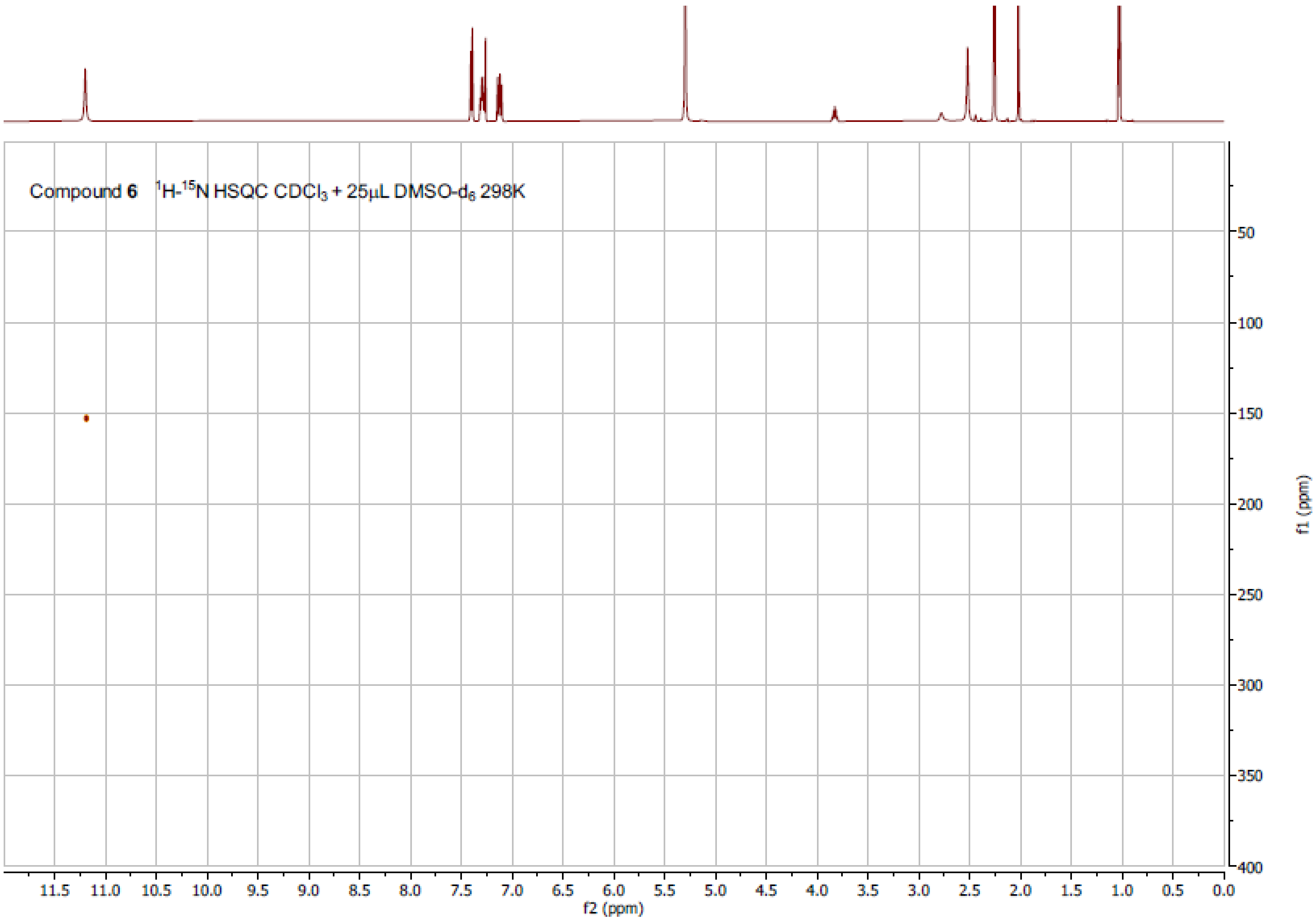

Figure S14. ^{1}H-^{15}N HSQC of compound **6**

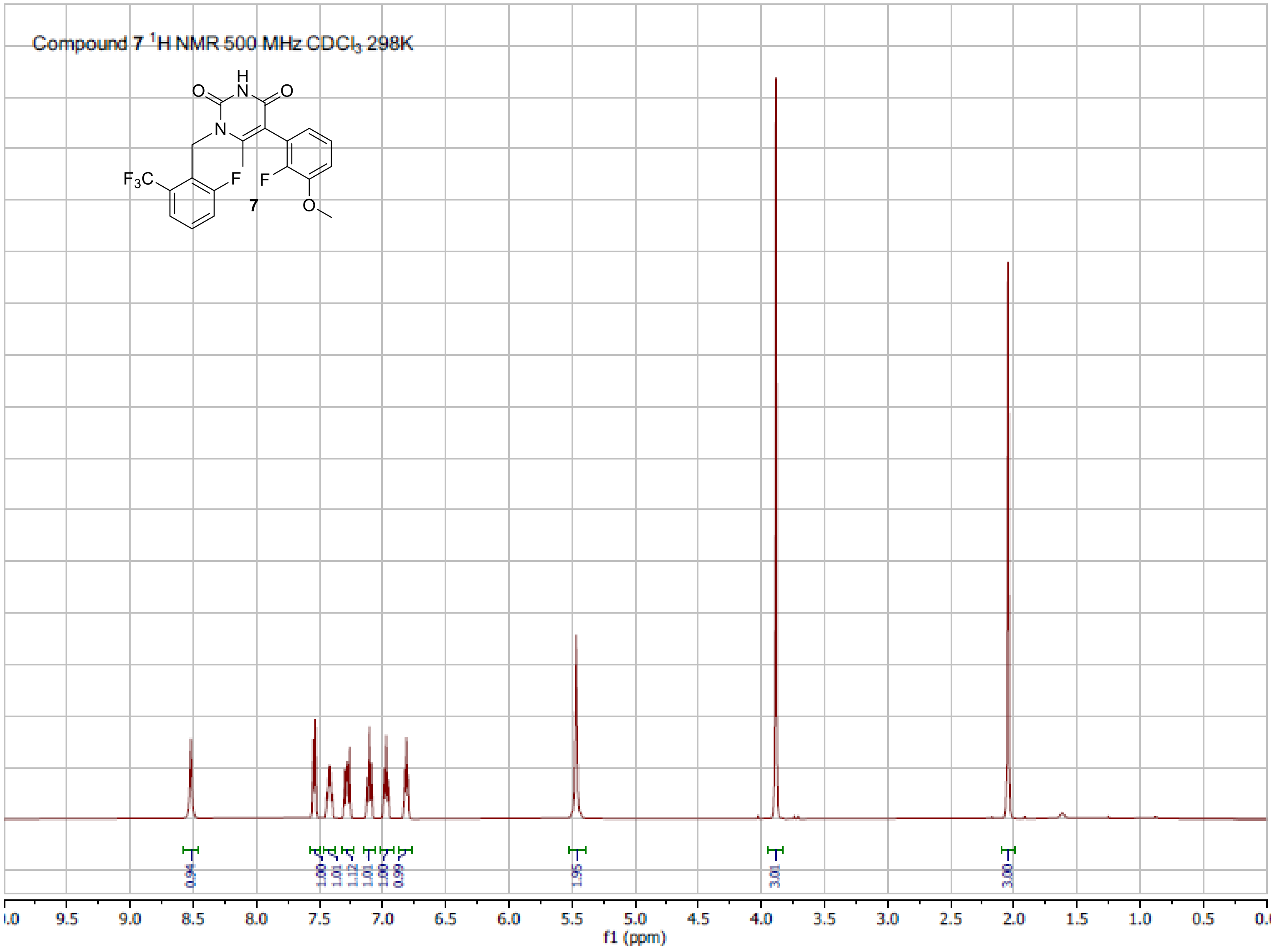

Figure S15. ^{1}H NMR of compound **7**

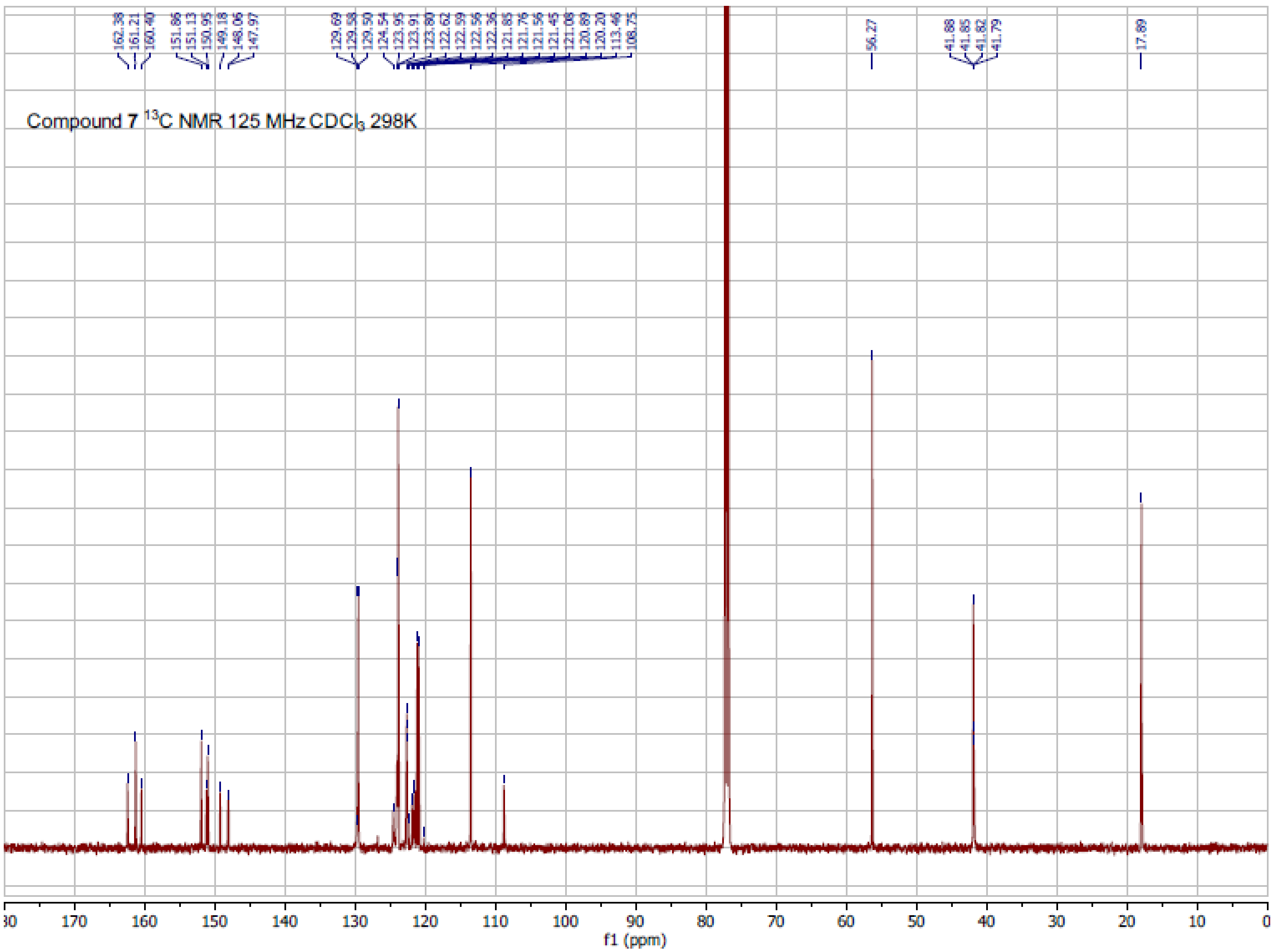

Figure S16. ^{13}C NMR of compound **7**

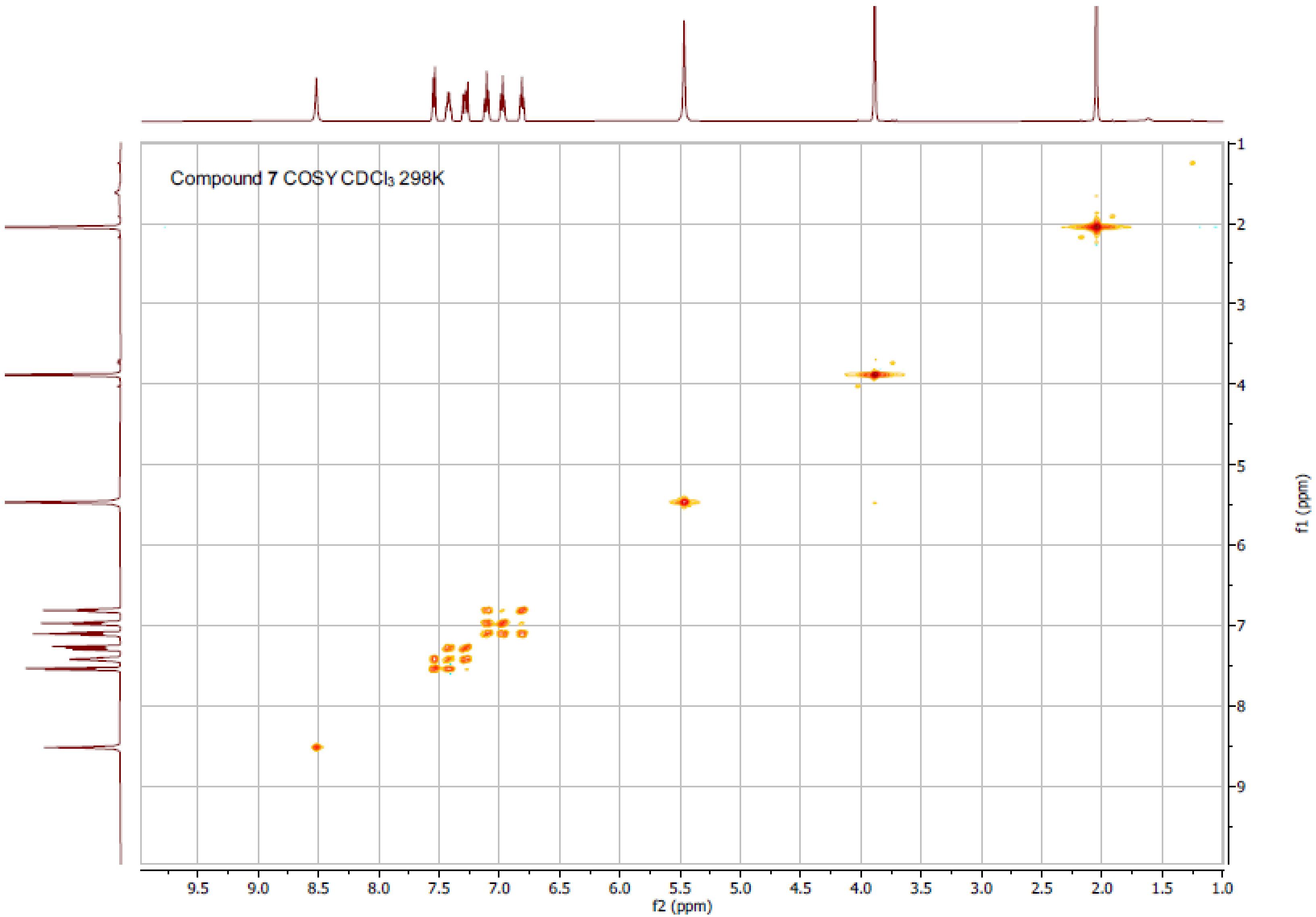

Figure S17. COSY of compound **7**

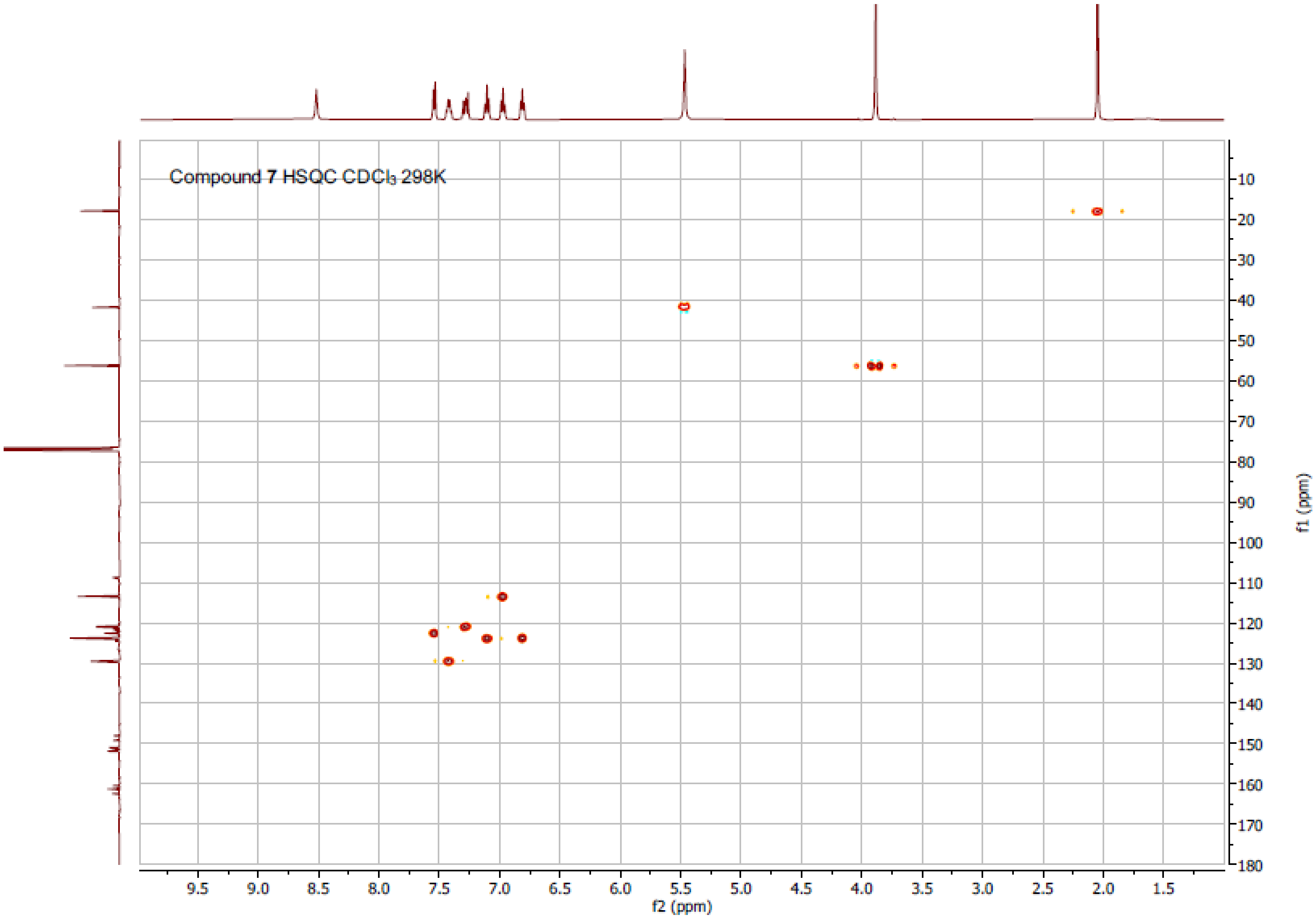

Figure S18. ^{1}H-^{13}C HSQC of compound **7**

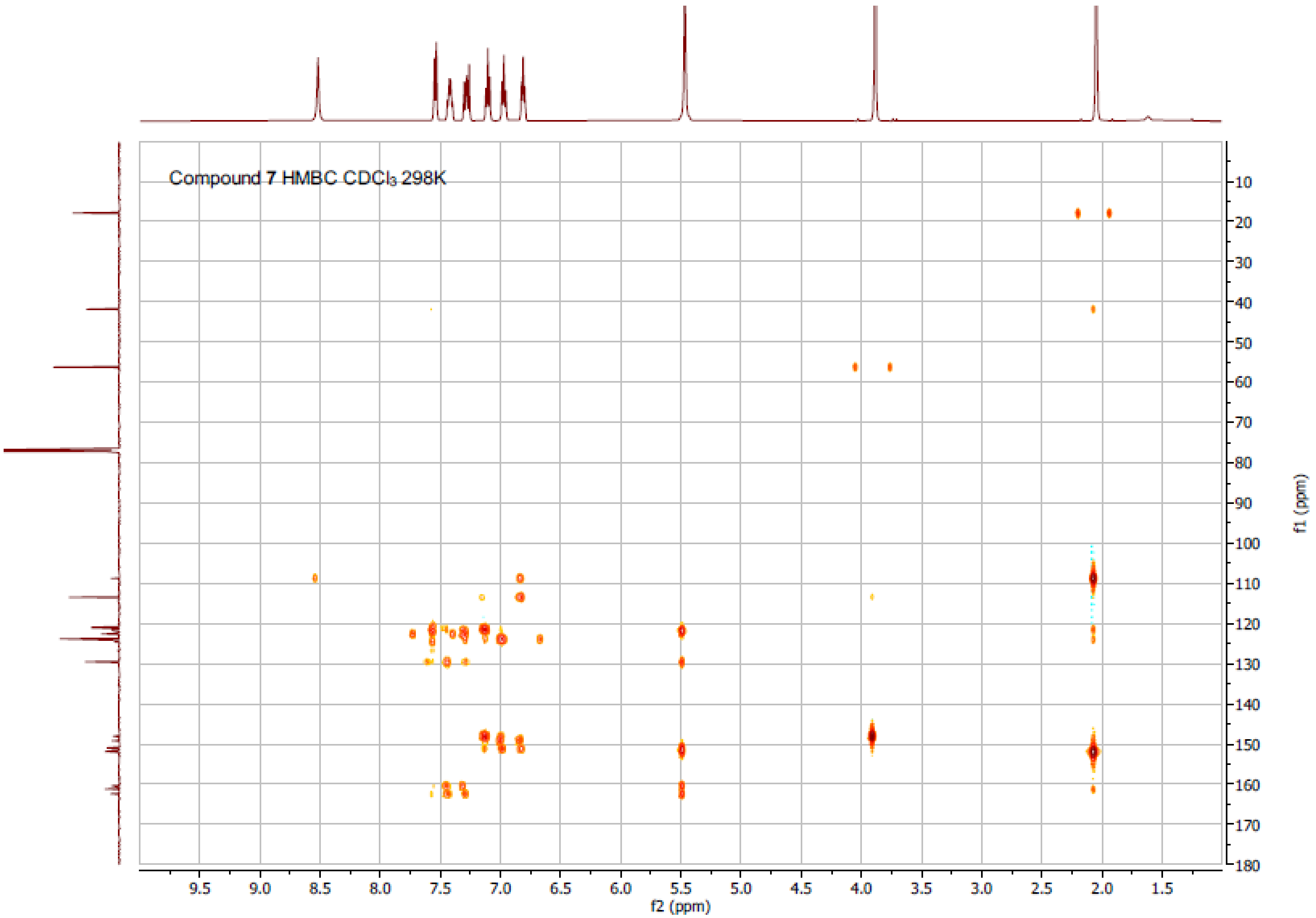

Figure S19. ^{1}H-^{13}C HMBC of compound **7**

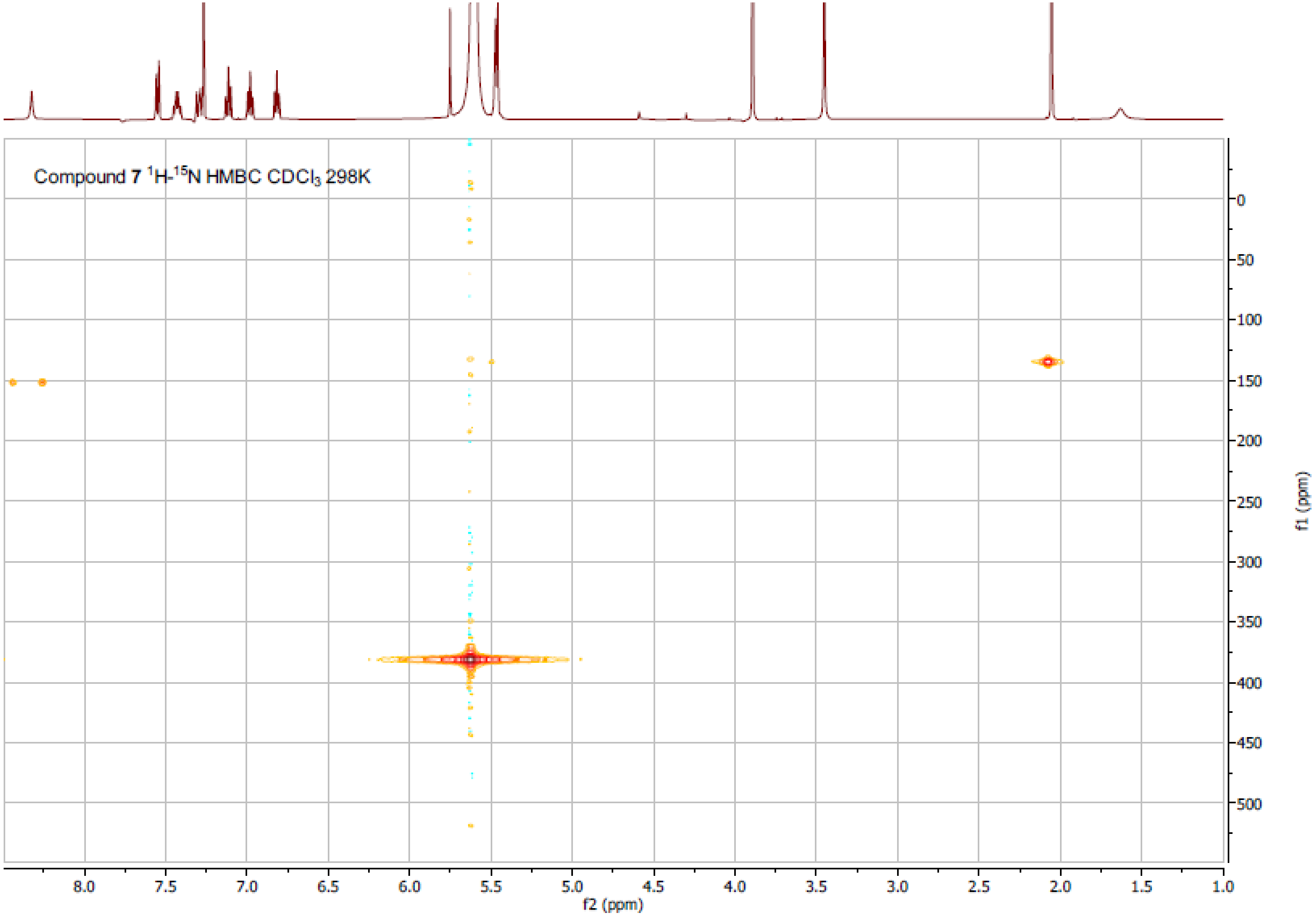

Figure S20. ^{1}H-^{15}N HMBC of compound **7**

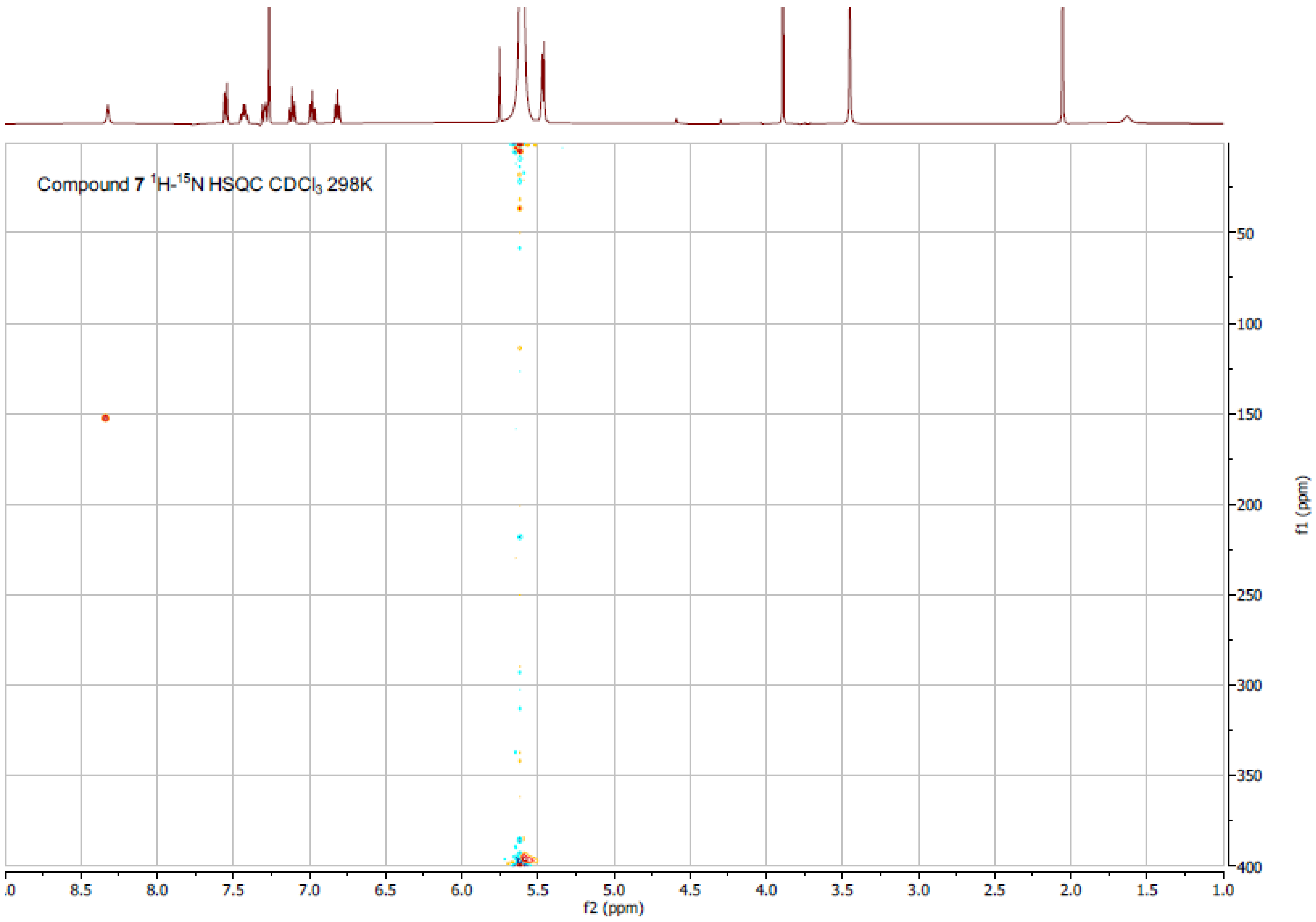

Figure S21. ^{1}H-^{15}N HSQC of compound **7**

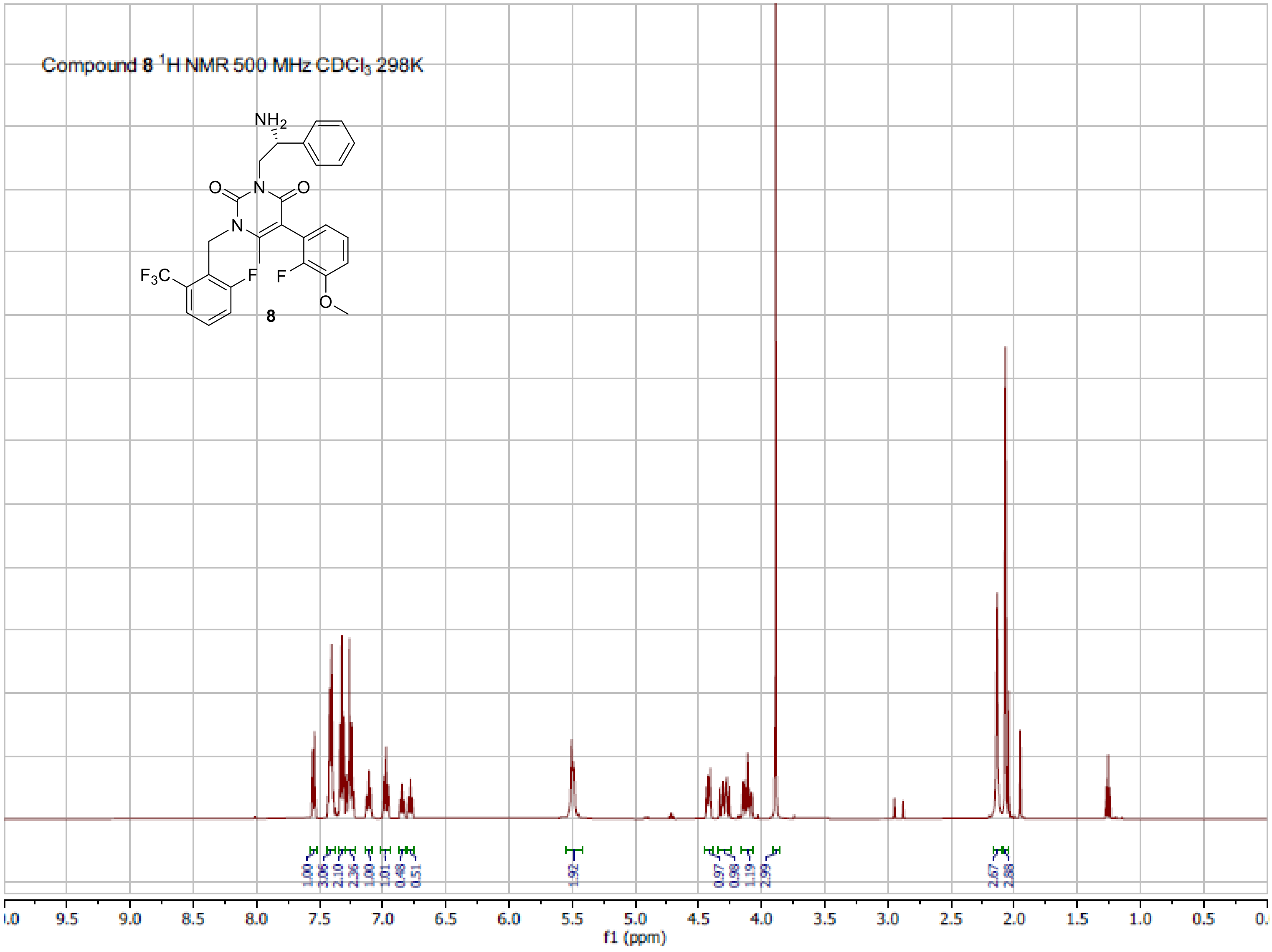

Figure S22. ¹H NMR of compound **8**

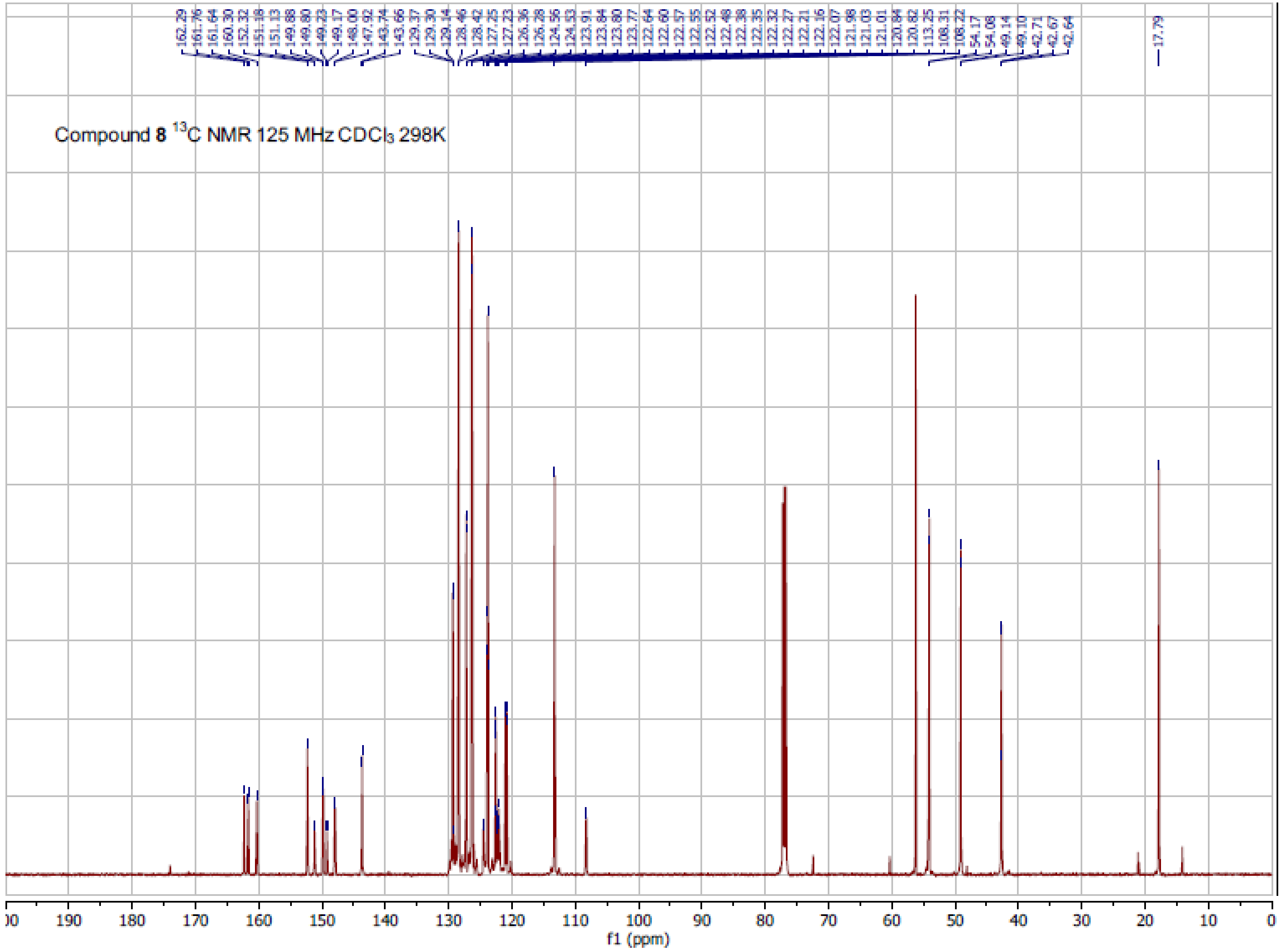

Figure S23. ^{13}C NMR of compound **8**

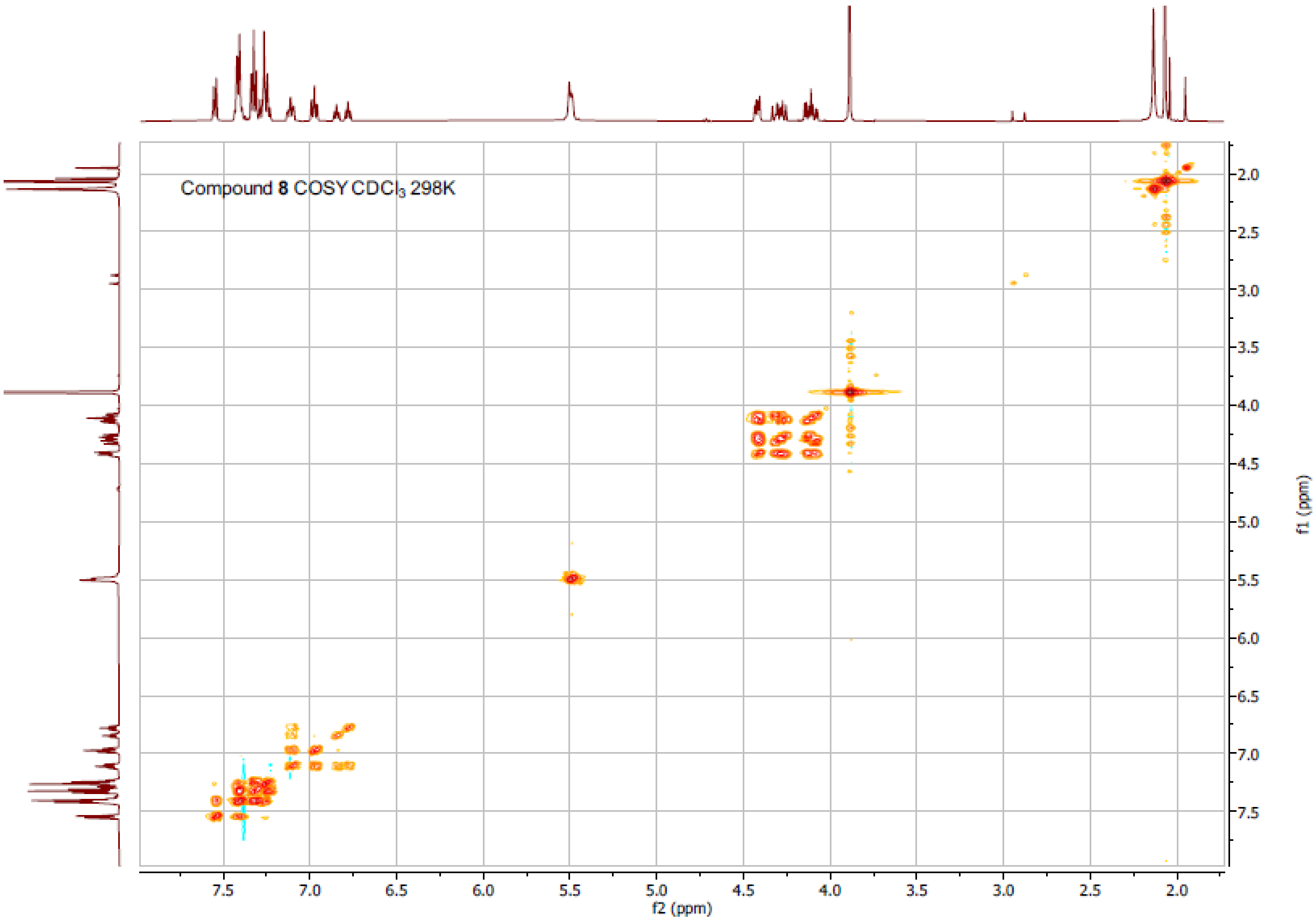

Figure S24. COSY of compound **8**

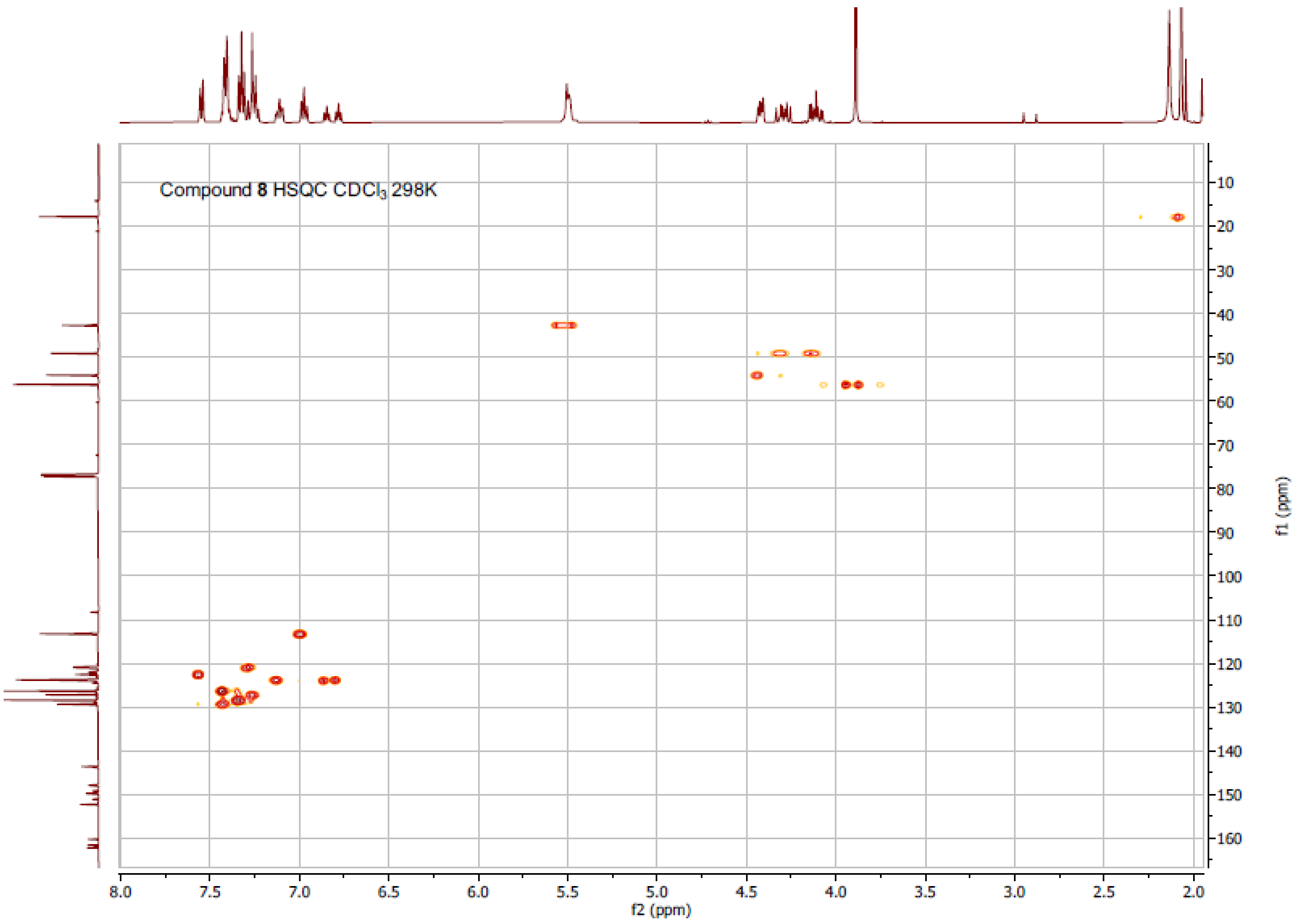

Figure S25. ^{1}H-^{13}C HSQC of compound **8**

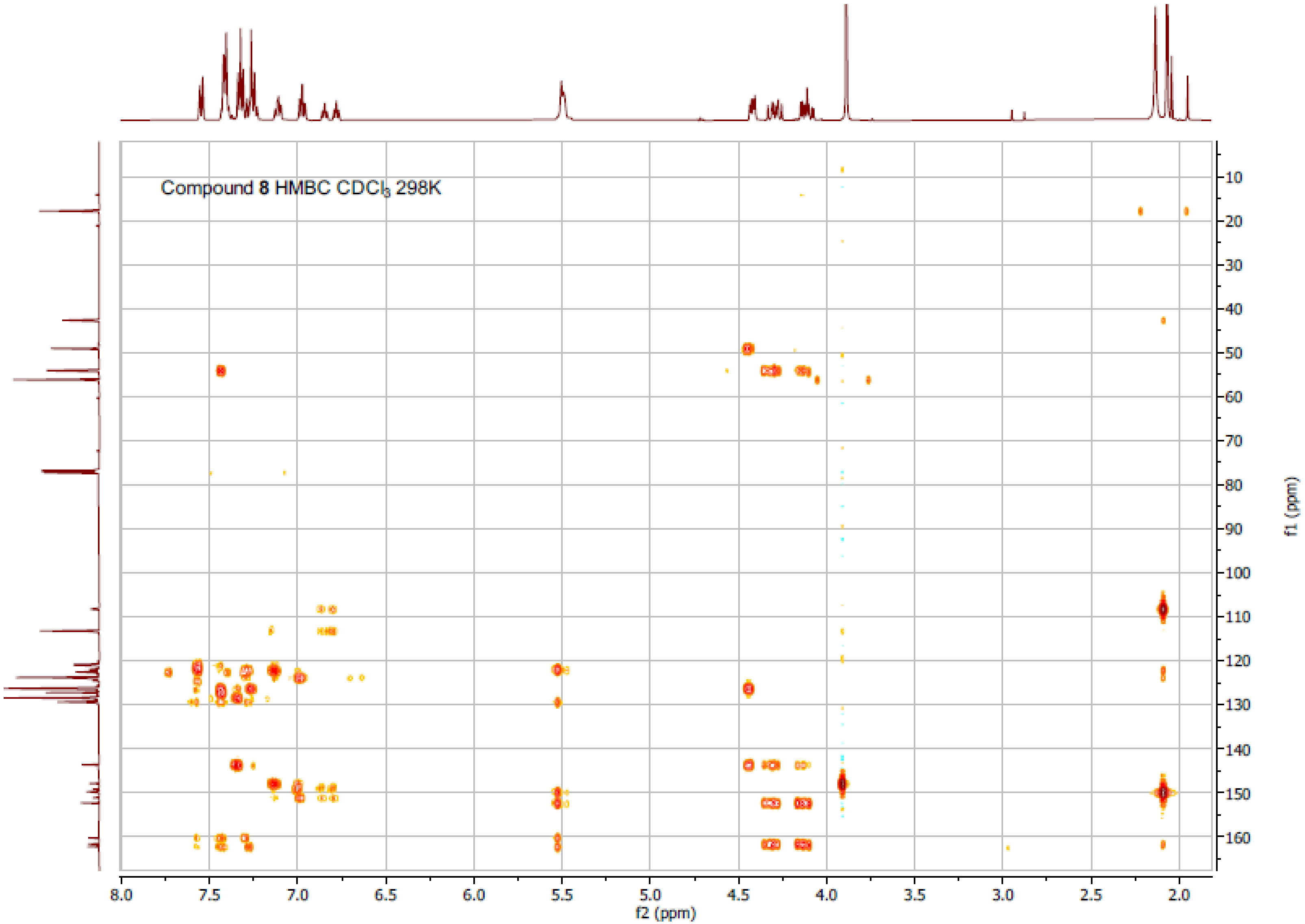

Figure S26. ^{1}H-^{13}C HMBC of compound **8**

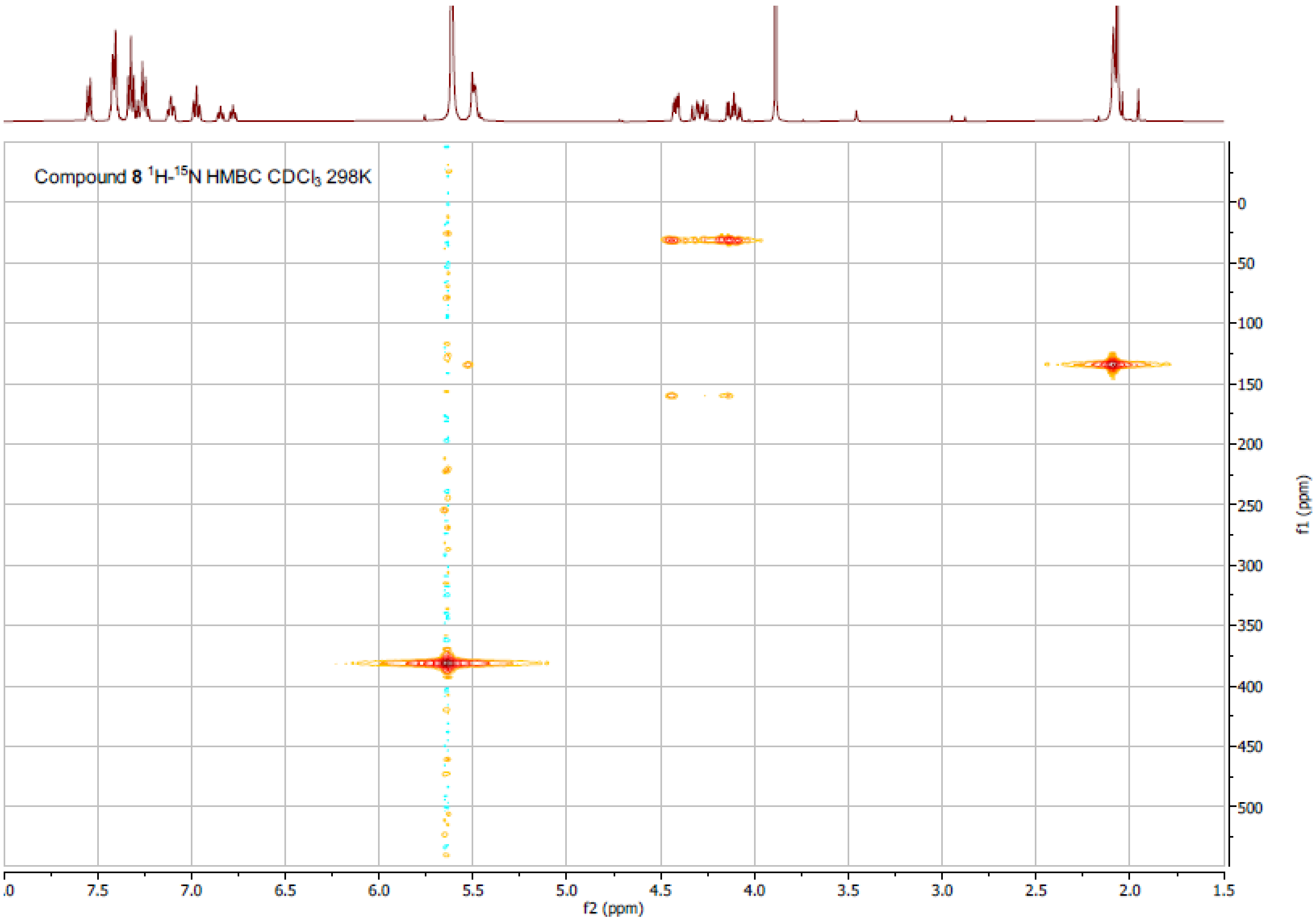

Figure S27. ^{1}H-^{15}N HMBC of compound **8**

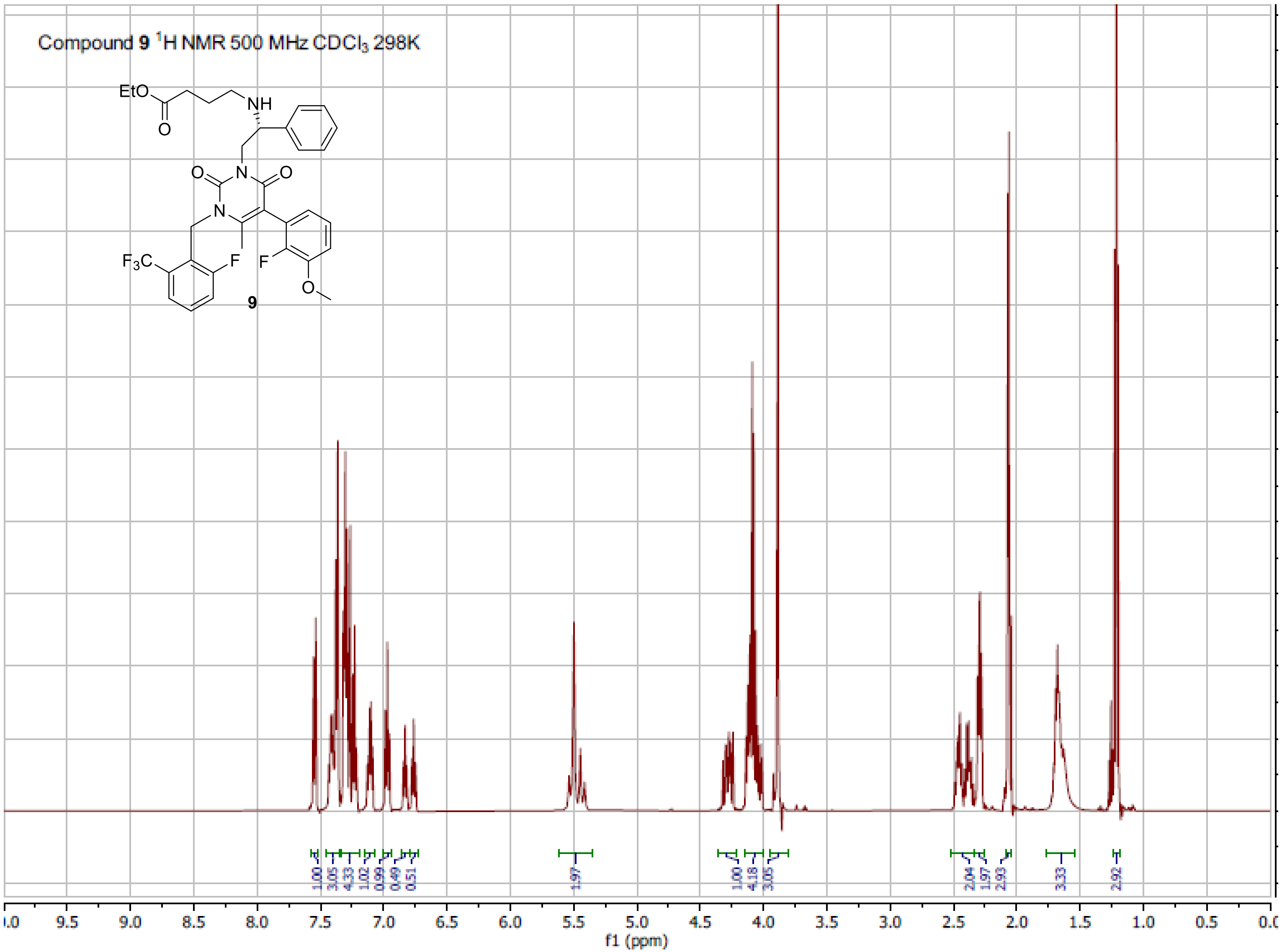

Figure S28. ¹H NMR of compound **9**

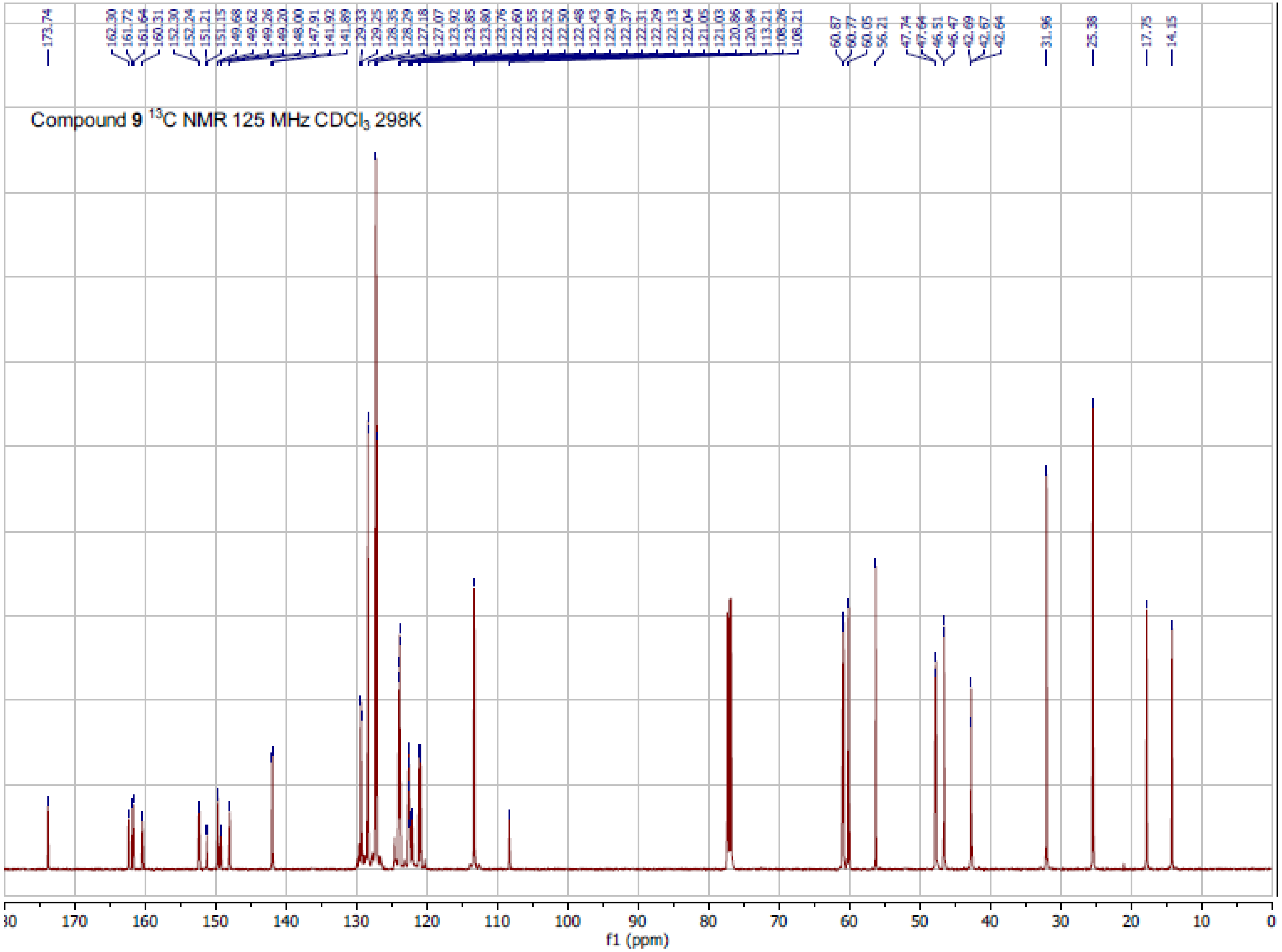

Figure S29. ^{13}C NMR of compound **9**

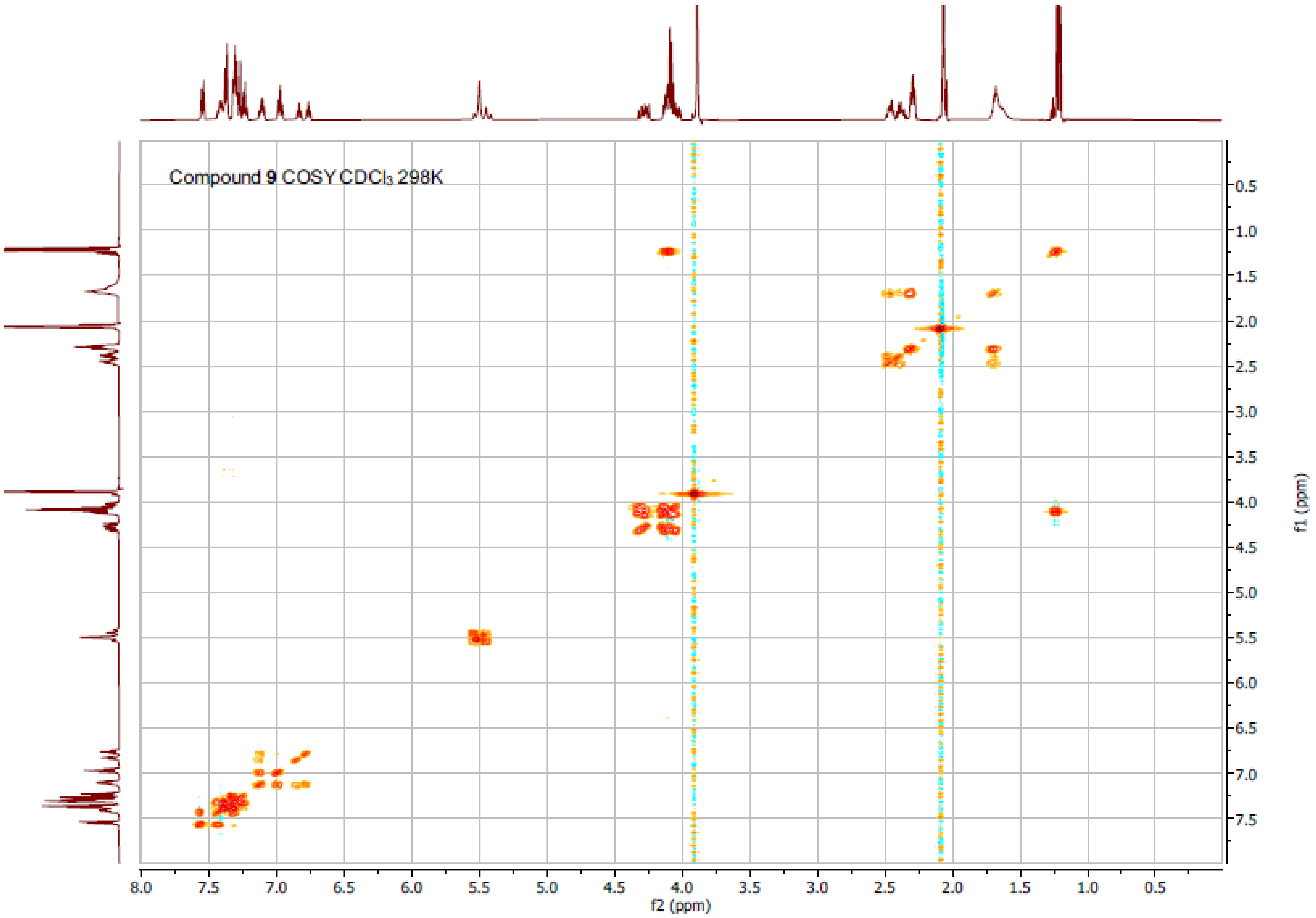

Figure S30. COSY of compound **9**

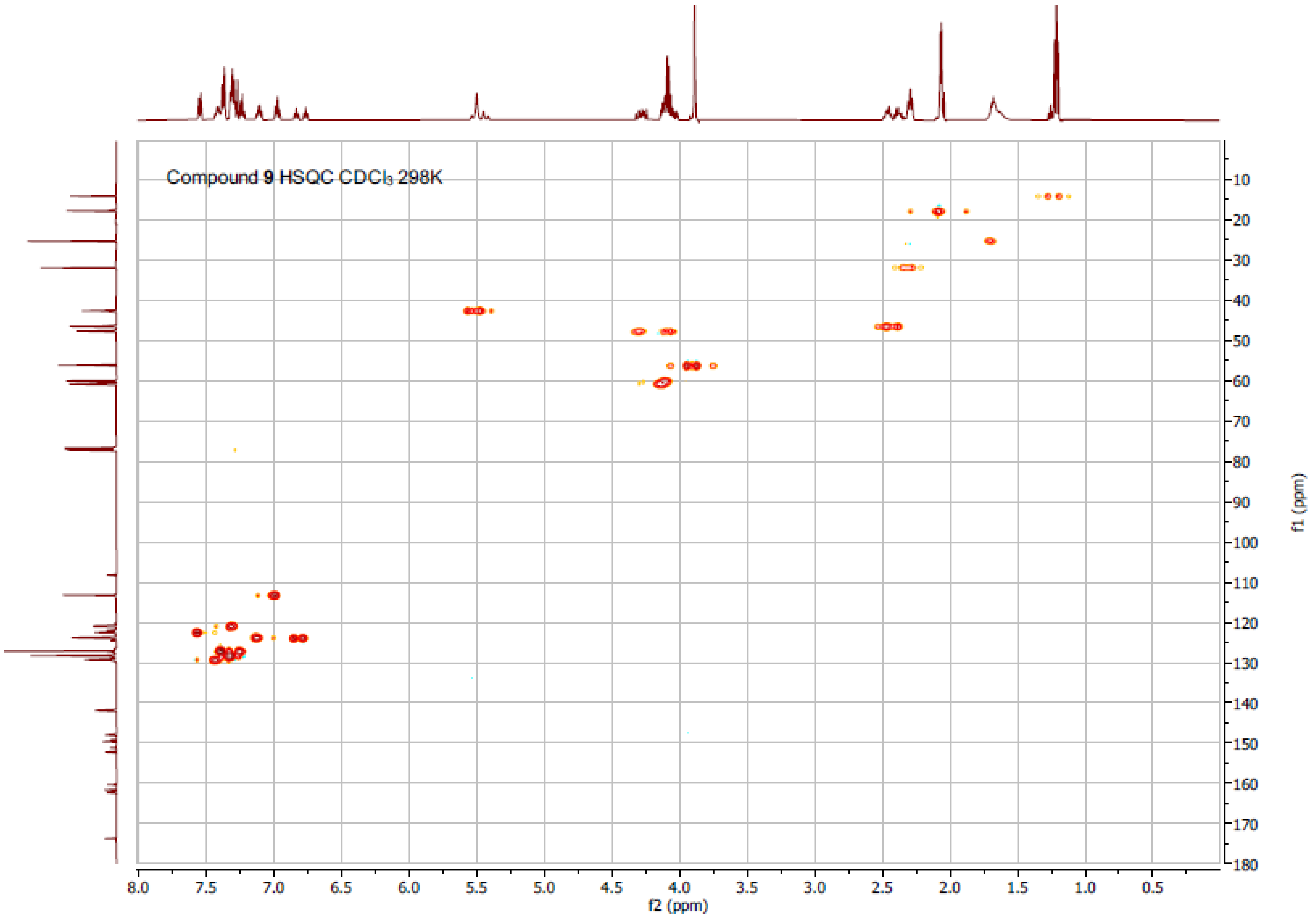

Figure S31. ^{1}H-^{13}C HSQC of compound **9**

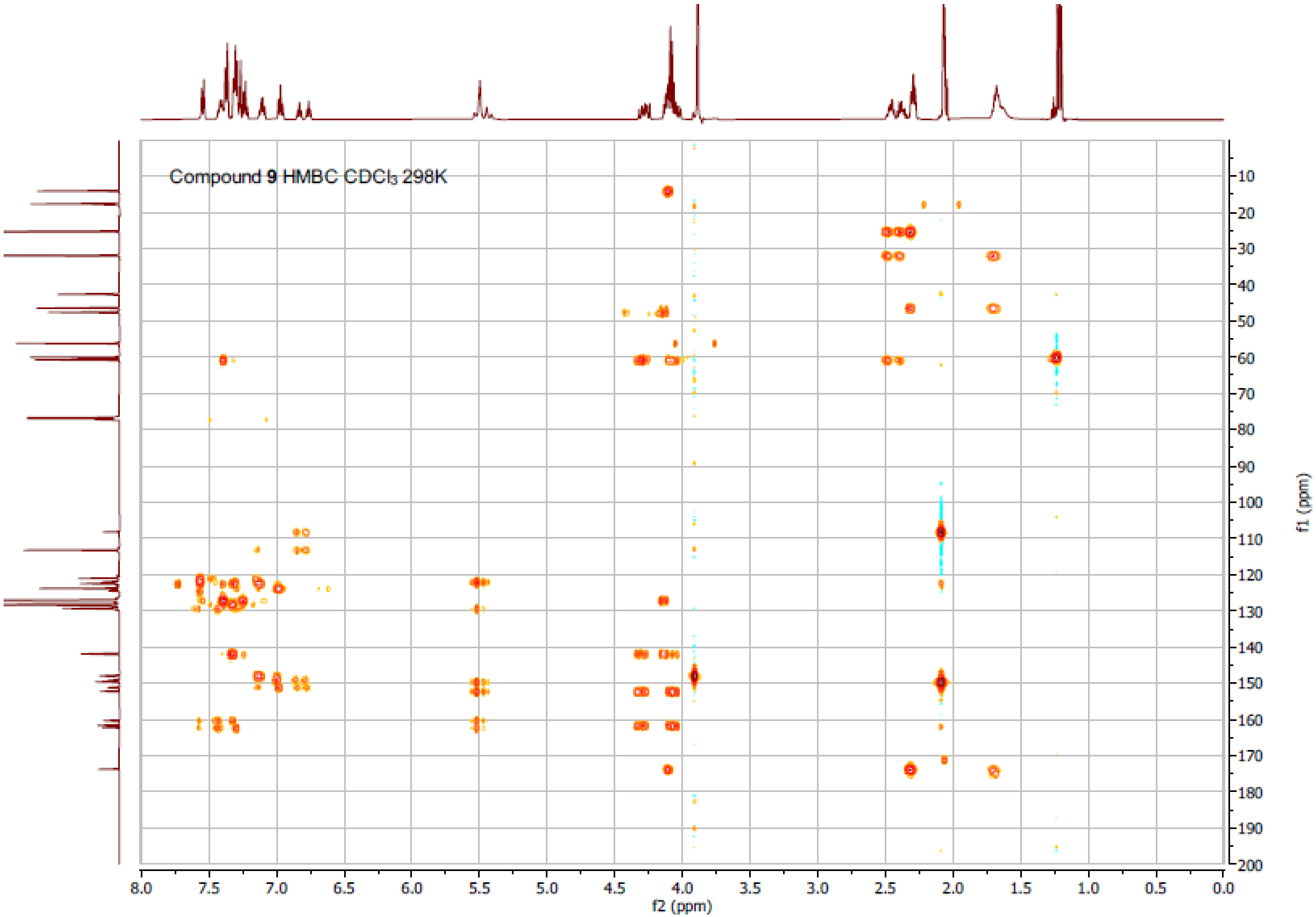

Figure S32. ^{1}H-^{13}C HMBC of compound **9**

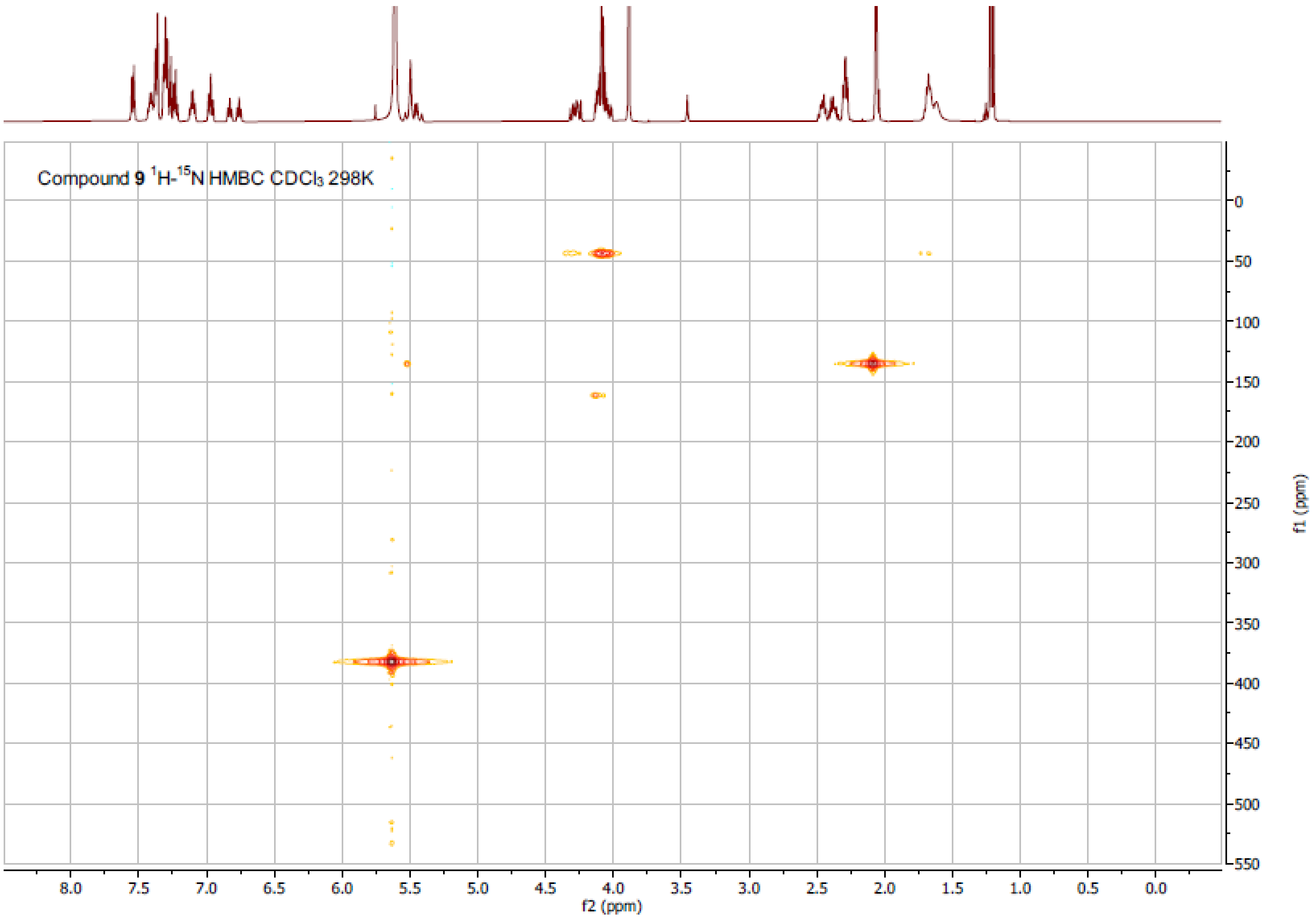

Figure S33. ^{1}H-^{15}N HMBC of compound **9**

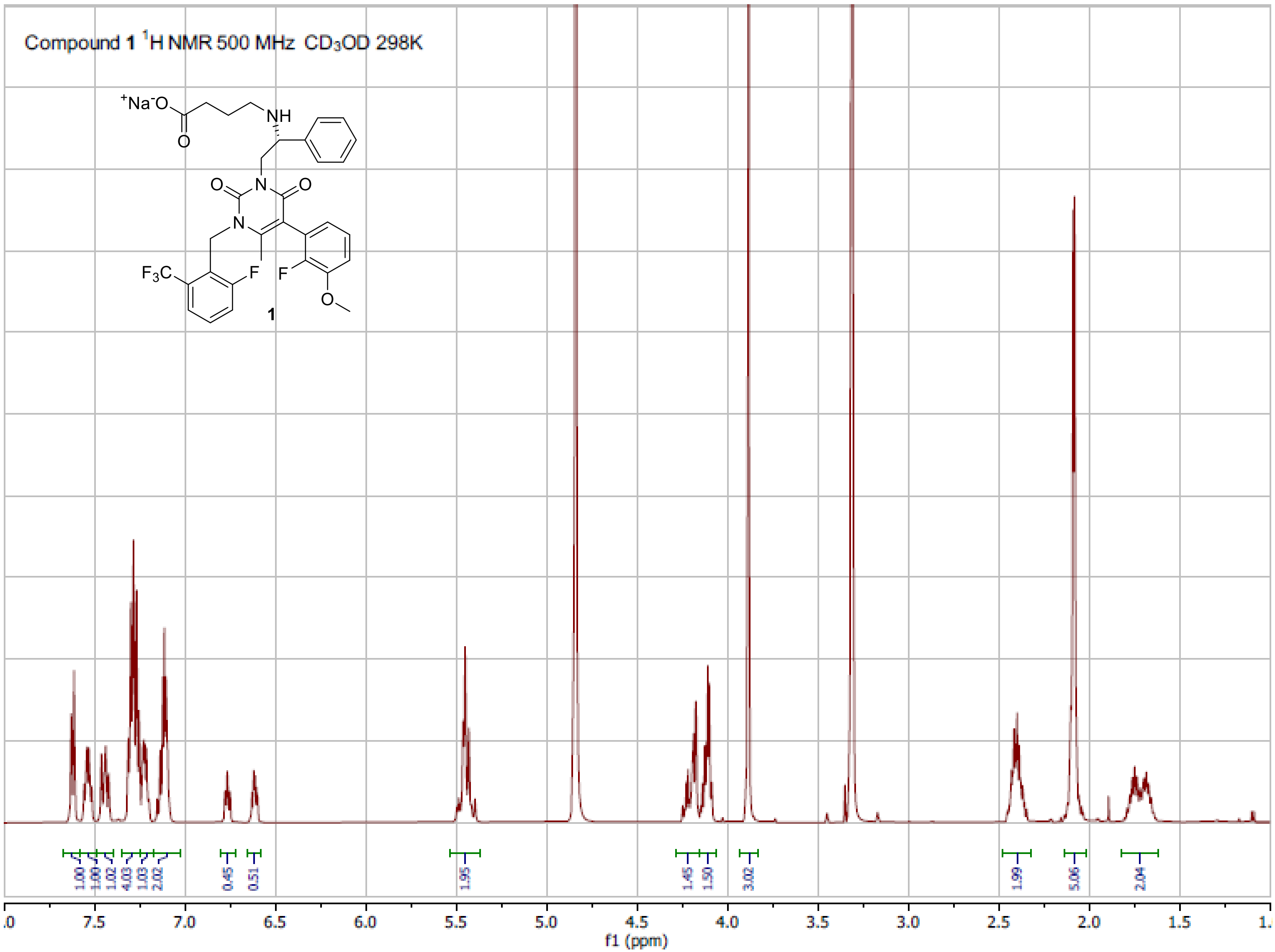

Figure S34. ¹H NMR of compound **1**

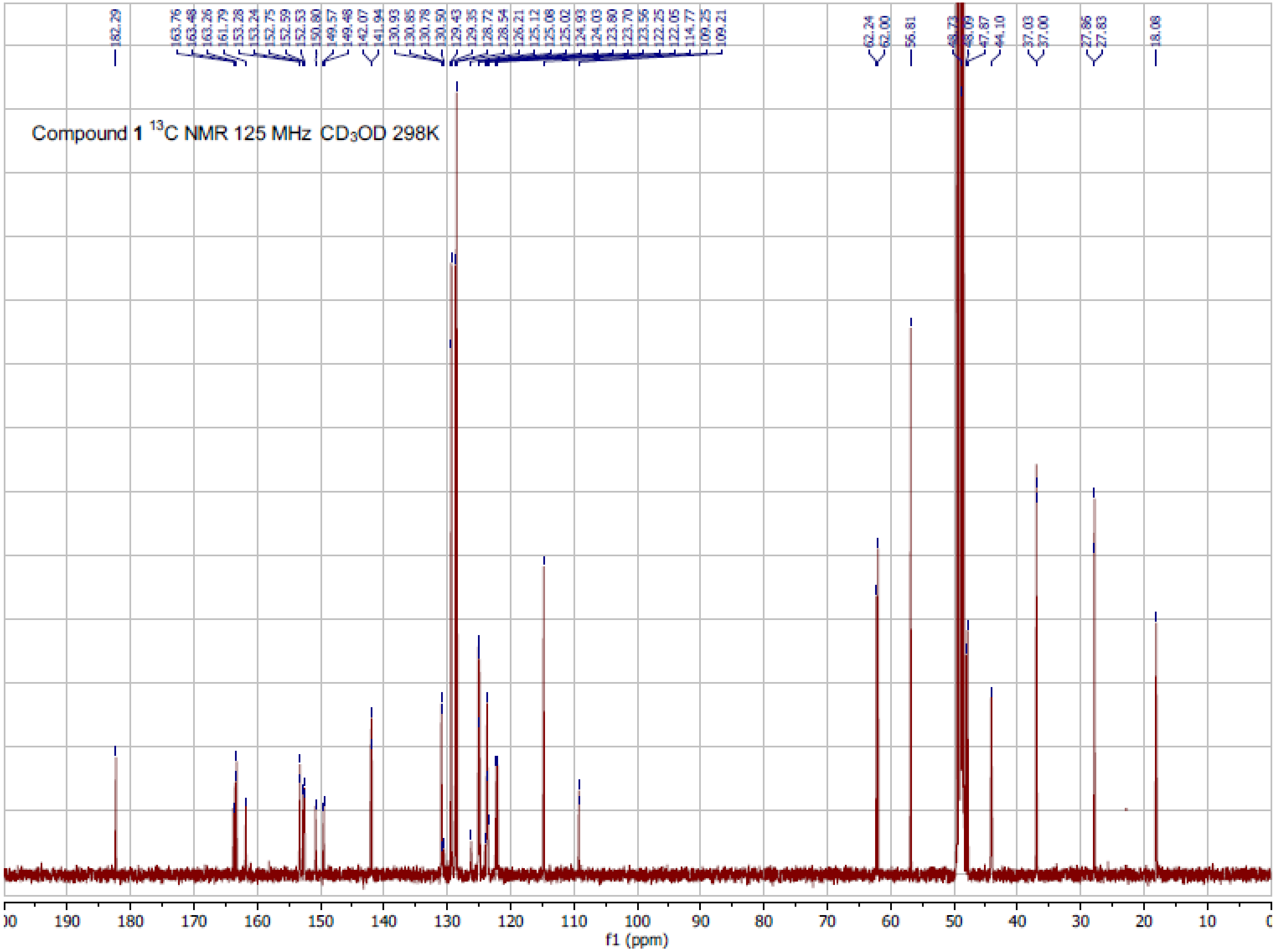

Figure S35. ^{13}C NMR of compound **1**

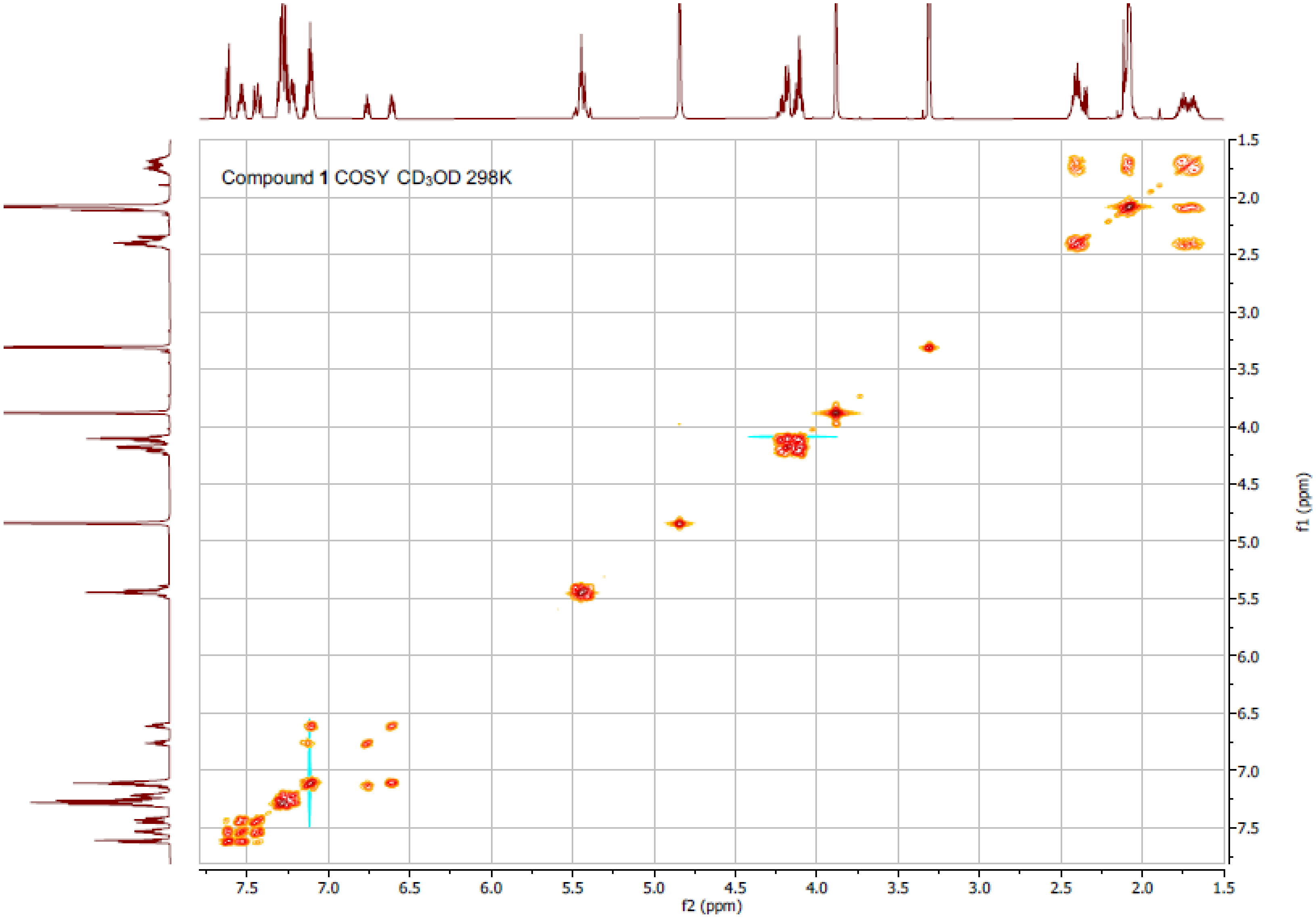

Figure S36. COSY of compound **1**

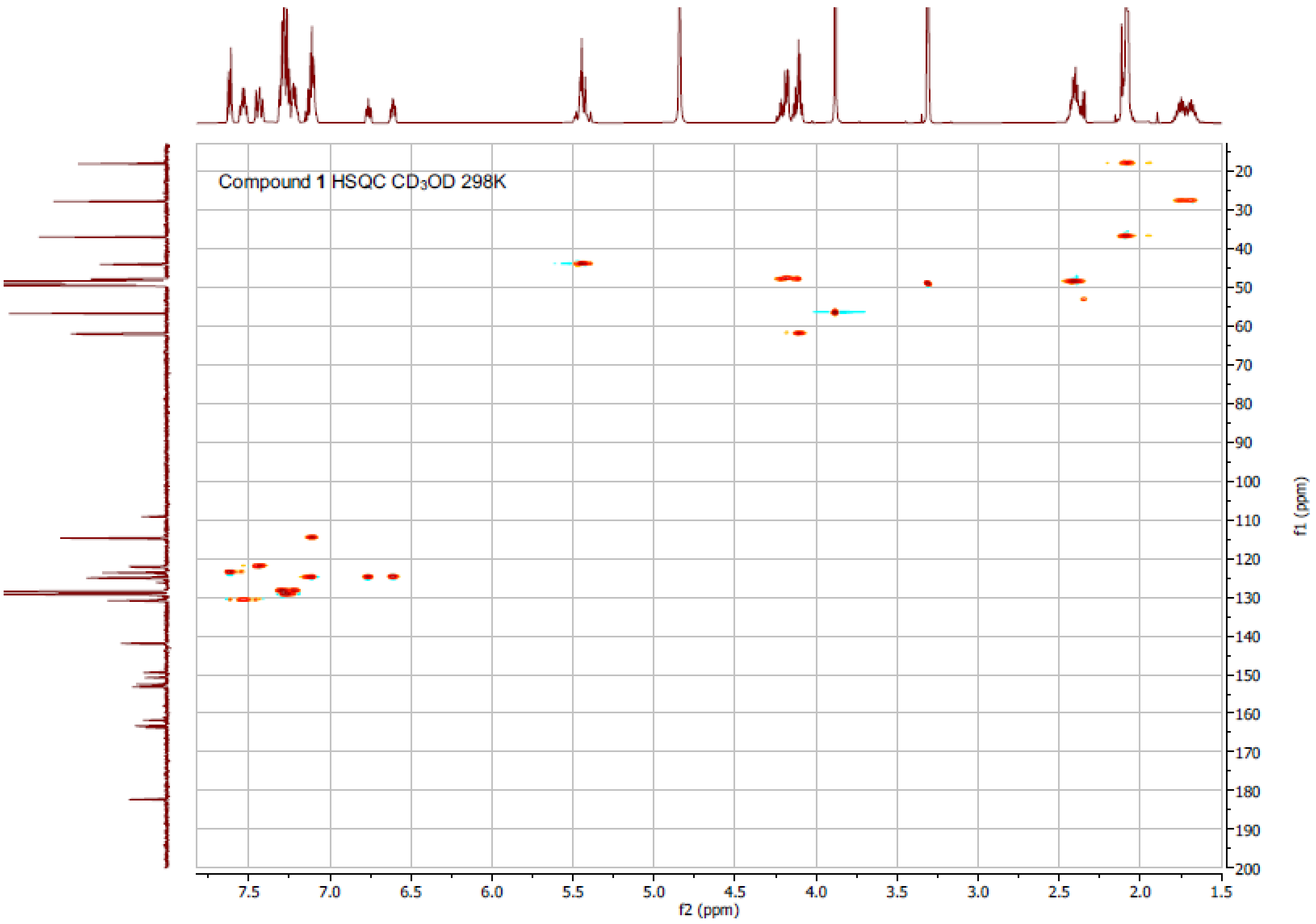

Figure S37. ^{1}H-^{13}C HSQC of compound **1**

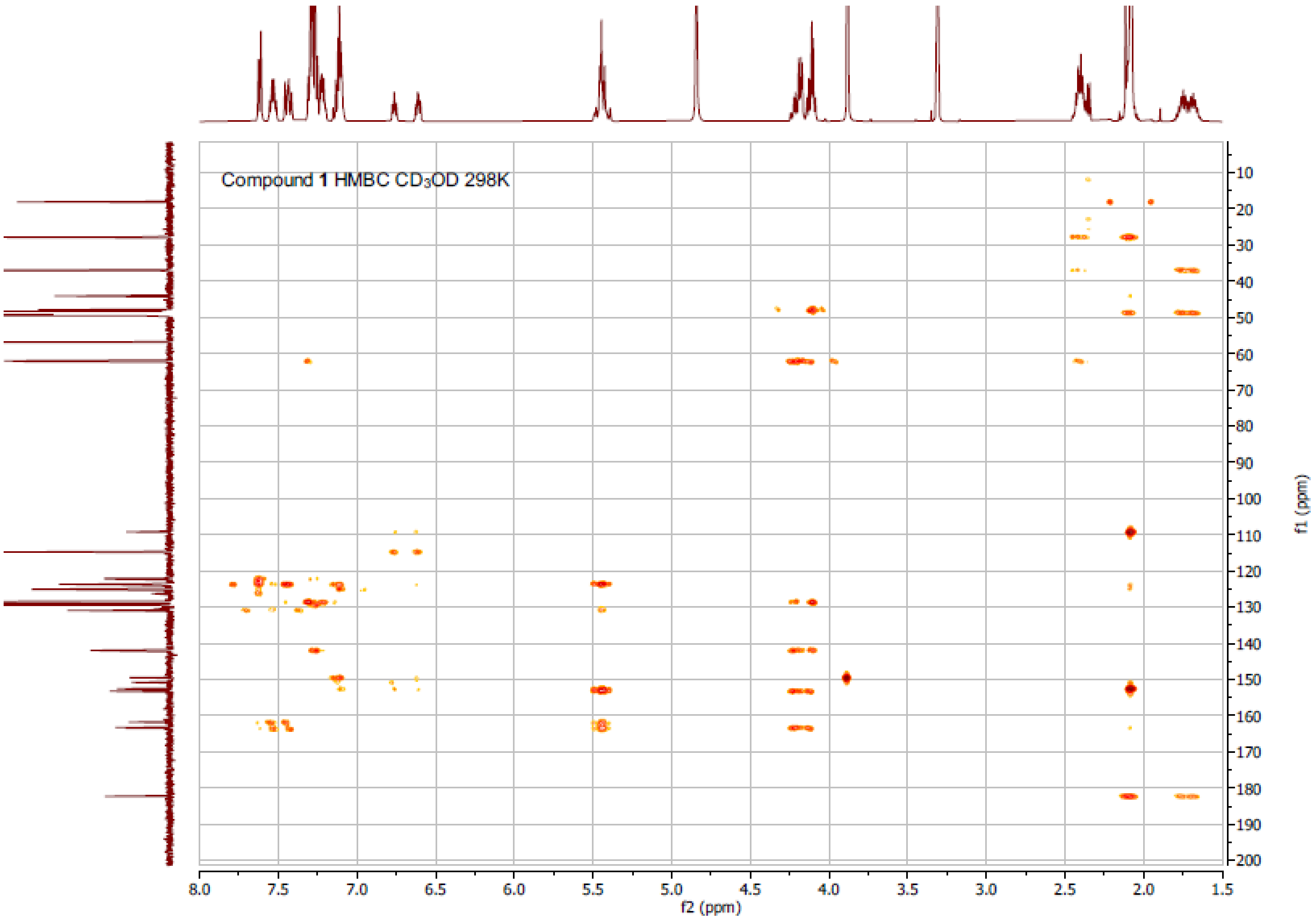

Figure S38. ^{1}H-^{13}C HMBC of compound **1**

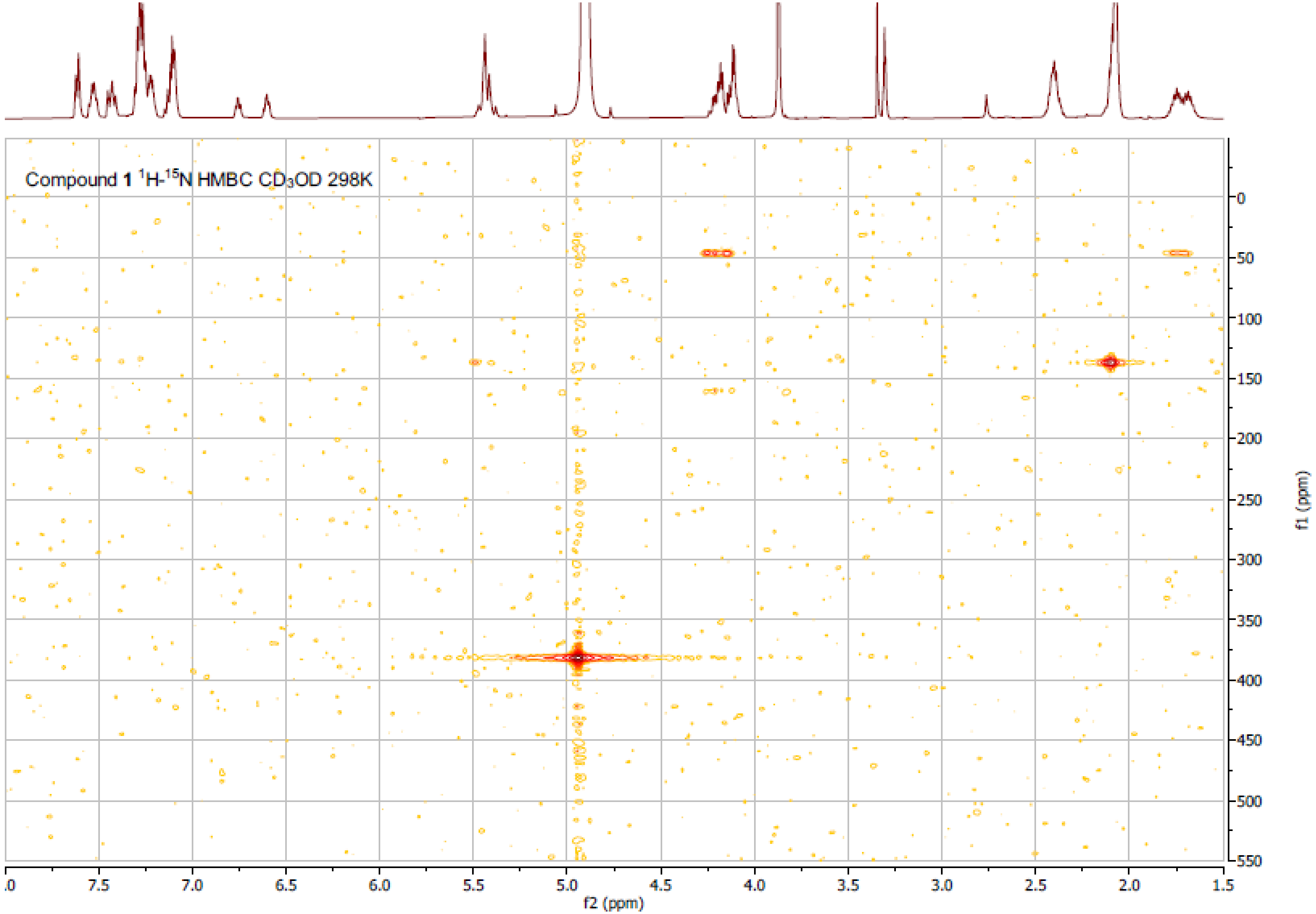

Figure S39. ^{1}H-^{15}N HMBC of compound **1**

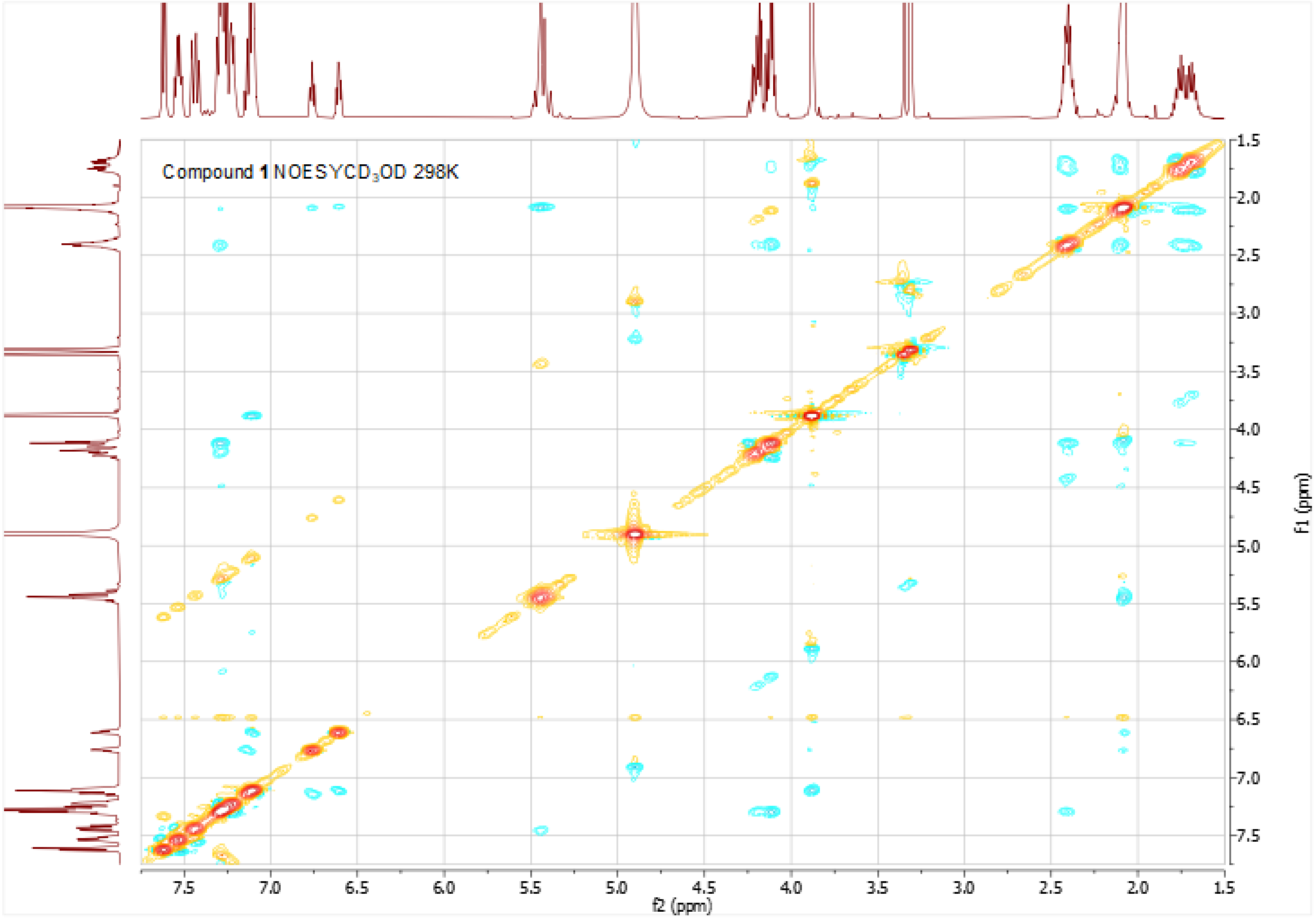

Figure S40. NOESY of compound **1**

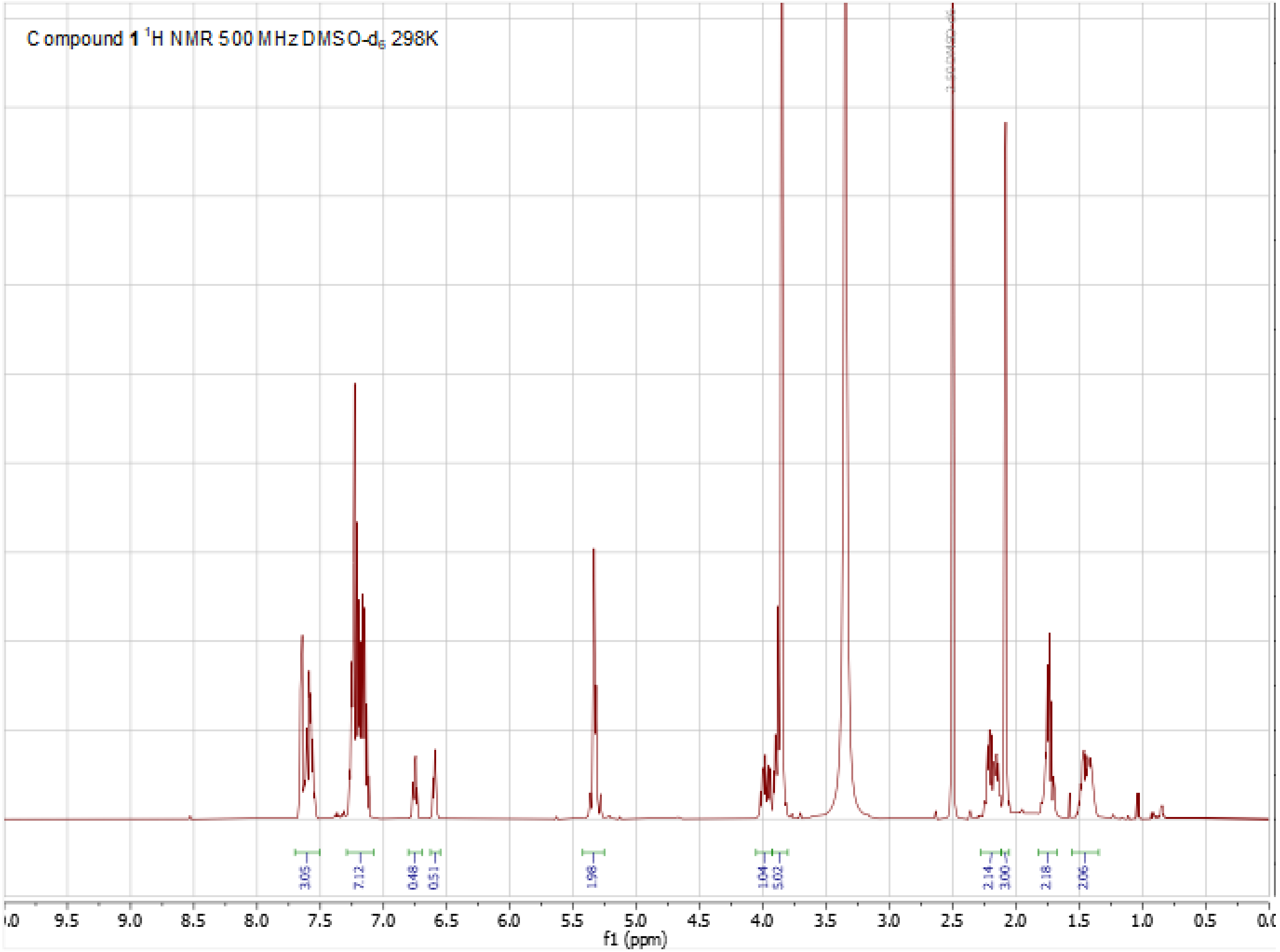

Figure S41. ^{1}H NMR of compound **1** in DMSO-d_6

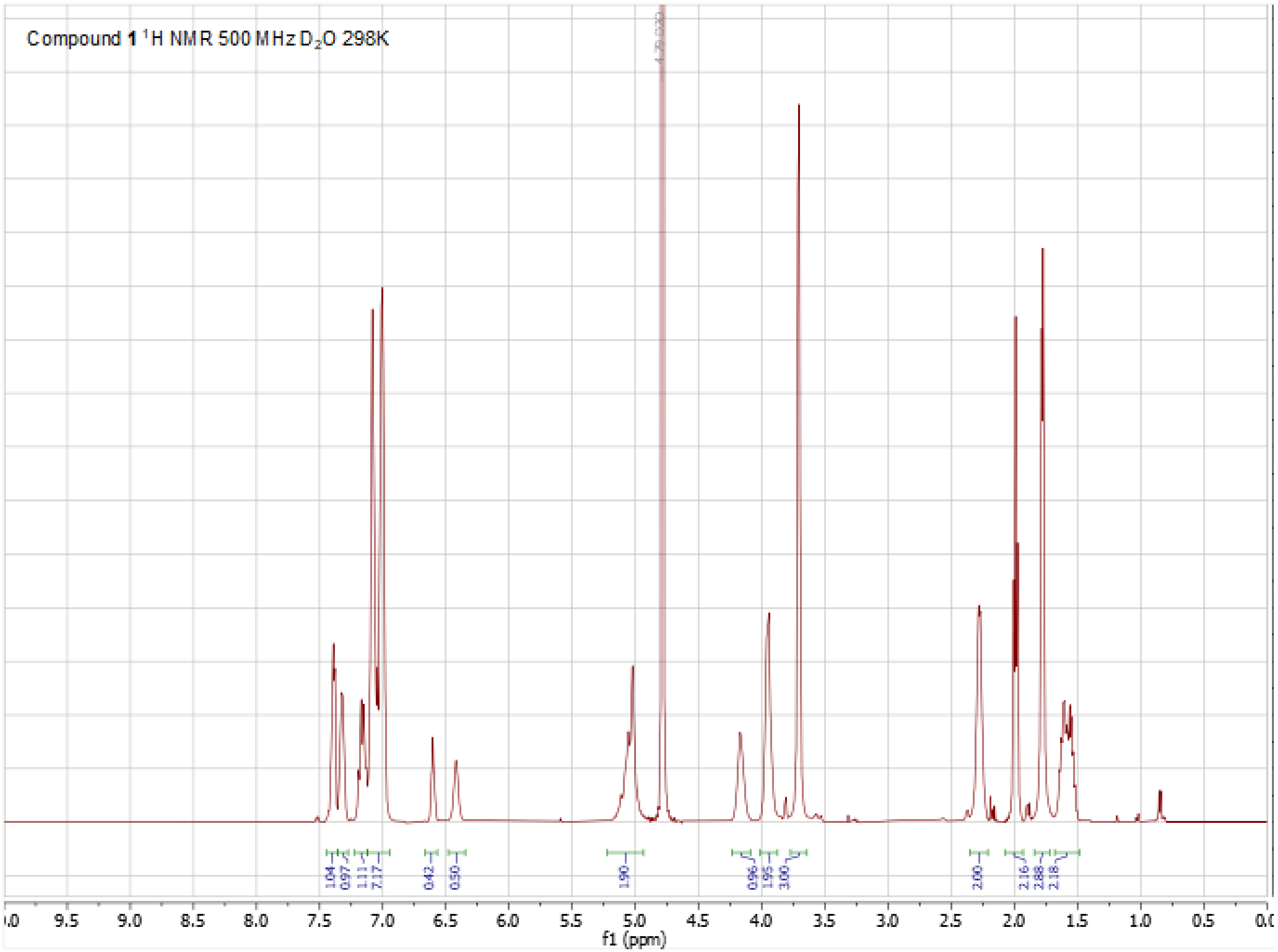

Figure S42. ^{1}H NMR of compound **1** in D_2O

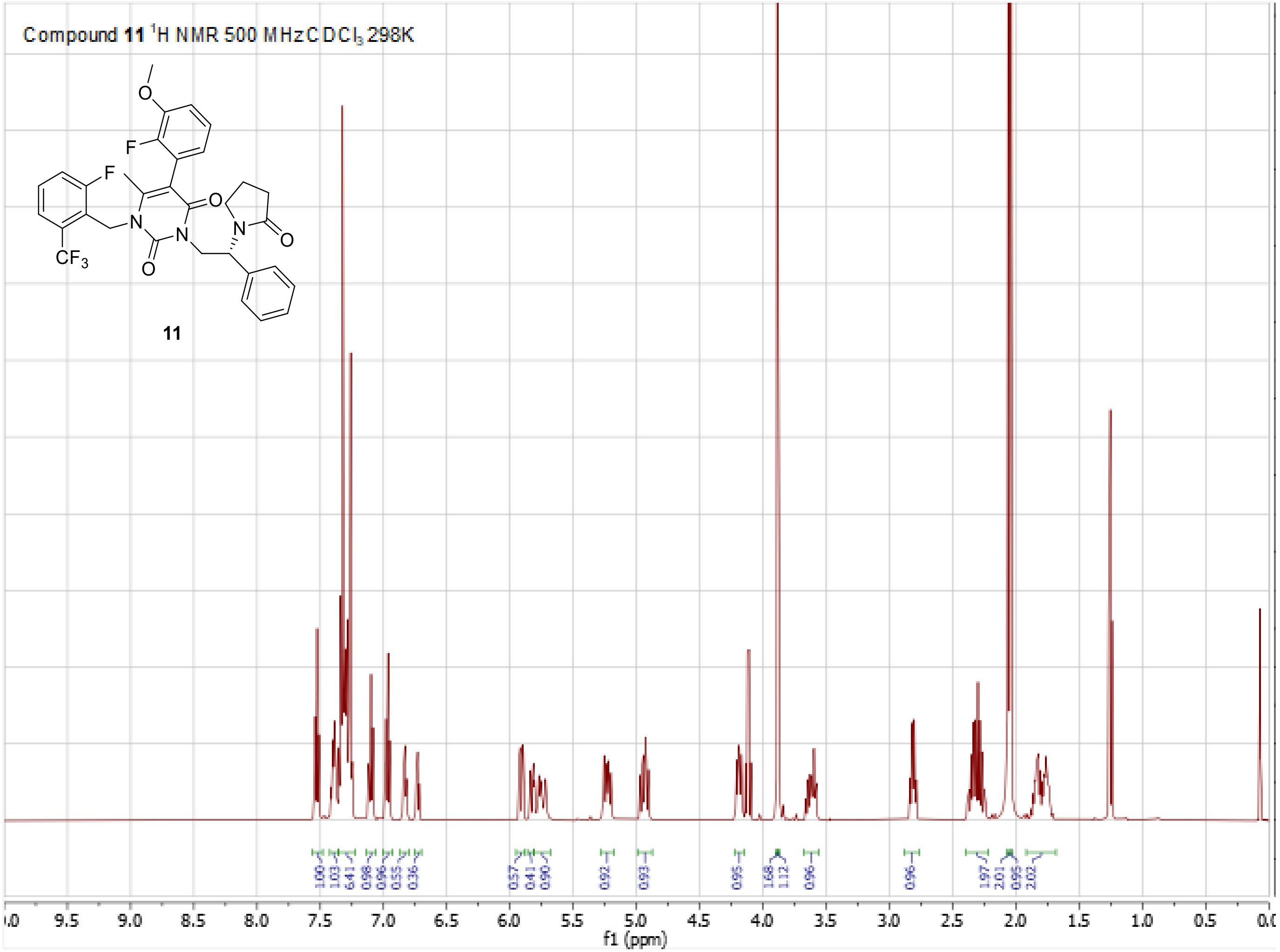

Figure S43. ¹H NMR of compound **11**

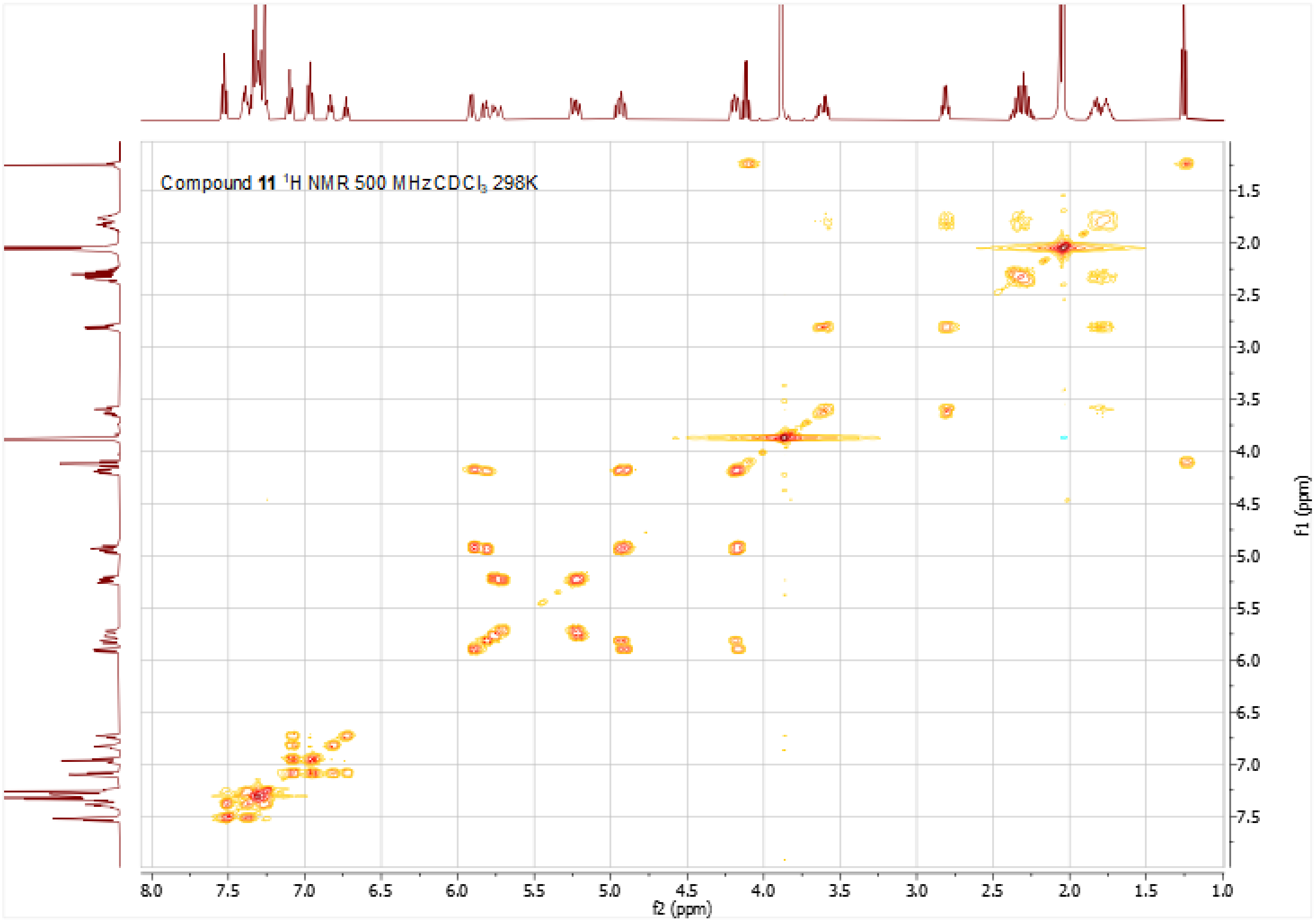

Figure S44. COSY of compound **11**

Conformers	Water ΔE (kcal/mol)	Water (%)	Methanol ΔE (kcal/mol)	Methanol (%)
**1A**	4.65	0.0	4.68	0.0
**1C**	0.84	18.4	0.73	21.2
**1D**	4.74	0.0	4.72	0.0
**1E**	7.88	0.0	n.c.	n.c.
**1H**	2.54	1.0	2.52	1.0
**1I**	2.66	0.9	2.64	0.8
**1J**	5.68	0.0	n.c.	n.c.
**1K**	5.42	0.0	n.c.	n.c.
**1L**	4.61	0.0	4.58	0.0
**1M**	0.00	75.6	0.00	73.0
**1O**	4.22	0.1	4.20	0.1
**1Q**	4.45	0.0	4.43	0.0
**1S**	1.93	2.9	1.92	2.9
**1T**	4.30	0.1	4.28	0.1
**1U**	4.59	0.0	4.58	0.0
**1V**	4.27	0.1	4.32	0.0
**1W**	2.75	0.7	2.74	0.7

[1]n.c. = not calculated.

Table S1. Relative energies and equilibrium percentages of the optimized conformations endowed with ΔE < 5 kcal/mol in the gas phase, in water and methanol solvent models.

Internuclear H-21/H-15 distance estimated by 2D NOESY				Internuclear distances of each H-15 proton from H-21 estimated by conformational analyses [3]
Atropisomer	H-21/H-15 cross peak intensity [1]	H-21/H-20 cross peak intensity	H-21/H15 distance [2]	
1-a*R*	-174489.7	-2699647.4	3.9 Å	3.89 Å - 4.90 Å - 5.30 Å (Average value = 4.70 Å)
1-a*S*	-161152.2	-2364674.9	3.9 Å	3.95 Å - 5.33 Å - 4.91 Å (Average value = 4.73 Å)

[1] One-third of the intensity of the cross peak; [2] The following general formula for quantitative distance information by measurement of the cross-peak intensities, within the initial rate approximation, was used: $r_{H-21/H-20}/r_{H-21/H-15}=(a_{H-21/H-15}/a_{H-21/H-20})^{1/6}$; r= internuclear distance, a= intensity (volume). The internuclear distance between the aromatic protons H-21 and H-20 was fixed to 2.47 Å as reported in *Journal of Molecular Liquids* **2022**, *367*,120481. [3] see experimental section of the manuscript.

Table S2. Internuclear H-15/H-21 distance estimated by 2D NOESY experiment.
